# Supplementary material for: Community-based fact-checking reduces the spread of misleading posts on X (formerly Twitter)
Source: Nat Commun. 2026 May 5;17:4070. doi: 10.1038/s41467-026-72597-0 (PMC13144318; doi:10.1038/s41467-026-72597-0)
Supplement: Supplementary file 1 — Supplementary Information [file 41467_2026_72597_MOESM1_ESM.pdf]

Supplementary Information for "Community-based fact-checking reduces the spread of misleading posts on X (formerly Twitter)"

Yuwei Chuai[†1], Moritz Pilarski[†2], Thomas Renault[†3], David Restrepo-Amariles[4], Aurore Troussel-Clément[4], Gabriele Lenzini[1], and Nicolas Pröllochs[⋆2]

[1]University of Luxembourg, Luxembourg, Luxembourg

[2]JLU Giessen, Giessen, Germany

[3]Université Paris-Saclay, Paris, France

[4]HEC Paris, Paris, France

[†]These authors contributed equally to this work.

[⋆]Corresponding author: nicolas.proellochs@wi.jlug.de.

Contents

Supplementary Note 1: Data Overview **4**

Supplementary Note 2: Descriptive Statistics **7**

2.1 Fact-Checking Activity Over Time 9

2.2 Stability of Note Status 10

Supplementary Note 3: Identification of Topics **11**

Supplementary Note 4: Propensity Score Matching **13**

Supplementary Note 5: Estimation Results **17**

5.1 Two-Period ATTs 17

5.2 Parallel Test and Multi-Period ATTs 20

5.2.1 Estimation results and parallel trends 20

5.2.2 Sensitivity analyses for DiD estimates 27

Supplementary Note 6: Placebo Analyses **33**

Supplementary Note 7: Sensitivity Analyses **39**

7.1 Sensitivity Across Response Time 39

7.2 Sensitivity Across Months From Roll-Out 41

7.3 Sensitivity Across Rating Thresholds and Note Helpfulness Scores 44

7.4 Sensitivity Across Poster and Post Characteristics 49

Supplementary Note 8: Analysis of Overall Reduction **57**

Supplementary Note 9: Analysis of Reposting Mechanisms **58**

9.1 Collection of Reposter Information 58

9.2 Reposter Dataset 60
9.3 Parallel Trend and Treatment Effect 61
9.4 ATTs Across Reposter Characteristics 66
9.5 Sensitivity Across Poster and Reposter Characteristics 68

Supplementary Note 10: Robustness Checks **100**

10.1 ATT Estimation With Post-Level Fixed Effects 100
10.2 ATT Estimation With Zero-Inflated Negative Binomial Regression 100
10.3 Analysis With Restrictions of Note Scores and Pre-Display Engagement 103
10.4 Comparison to Previous Work Analyzing Aggregated Repost Counts 106
10.5 Alternative Before-Display Periods 110
10.6 Analysis With HonestDiD 113

Supplementary Note 11: Analysis of Deleted and Suspended/Protected Posts **117**

11.1 Analysis of Deleted Posts 117
11.2 Analysis of Protected/Suspended Posts 120

Supplementary Note 12: Changes in X (formerly Twitter)'s Algorithms and Policies on Misinformation **122**

12.1 Timeline of Changes 122
12.2 Accounting for Algorithmic Effects in Our Study 124

Supplementary Note 1: Data Overview

The "Community Notes" program on X (formerly Twitter) enables users to flag posts that they believe are misleading and contribute textual notes that provide context to the source post. After a community note is submitted, the fact-check becomes available for other enrolled contributors to rate while remaining hidden from the public users. To surface helpful community notes that appeal broadly across heterogeneous user groups, the program features a bridging-based rating system [1]. This system calculates the helpfulness score for each community note based on the ratings made by the contributors. Only notes that are rated as helpful by multiple contributors with heterogeneous rating histories are displayed to public users on X (formerly Twitter) [2]. Importantly, X (formerly Twitter)'s recommendation algorithm (available as open source [3]) does not impose any penalties (e.g., visibility reduction) on posts flagged with community notes [4]. This implies that any observed changes in resharing behavior after a community note becomes visible must be attributed to shifts in user behavior, rather than algorithmic intervention (see Supplementary Note 12 for details).

To evaluate the efficacy of community notes in reducing the spread of misleading posts on X (formerly Twitter), we gathered time series data on repost counts and fact-checking histories for 237,180 posts that have been fact-checked on X (formerly Twitter)'s "Community Notes" platform (see Methods). Our dataset covers all community fact-checked posts written in English between the roll-out of "Community Notes" to the general public on October 6, 2022, and June 11, 2024, i.e., for an observation period of over 20 months. For each post, we used the X (formerly Twitter) API v2 to retrieve the time series of repost histories at minute level over 36 hours after its creation, during which the vast majority of all reposts occured [5,6]. Throughout our observation period, the posts in our dataset had been reposted more than 431 million times. Subsequently, we combined the time series data on repost counts with fact-checking histories indicating at which time point the community notes were rated helpful and displayed to the public users. The data collection process

resulted in a longitudinal dataset comprising information about the repost counts at minute level and the current note status for each fact-checked post (see Supplementary Note 2 for summary statistics). This allows us to scrutinize the efficacy of community notes in reducing the spread of misleading posts on X (formerly Twitter).

Figure S1a shows that the "Community Notes" feature gained significant traction after its roll-out to the public on October 6, 2022. Both the number of community notes and fact-checked source posts increased significantly. On average, community note contributors fact-checked 386 posts in total per day. Here, multiple users can write notes for the same post. Therefore, the data sometimes includes multiple fact-checks for the same post. The average number of notes per fact-checked post was 1.867. Only a relatively small fraction of 10.1% of all notes was actually displayed to users, i.e., were surfaced as helpful by the rating system of "Community Notes." However, once community notes received the helpful status, they tended to remain displayed stably on the fact-checked posts (see Supplementary Note 2.2).

Compared to the rapid dissemination of posts on X (formerly Twitter), fact-checking via community notes was relatively slow. While 75.7% of all notes deemed helpful were displayed to general users within 36 hours after their creation, the time lag between post creation and note display (i.e., the response time) averaged at 62.9 hours (median of 18.1 hours; see Figure S1b). In contrast, posts on X (formerly Twitter) spread considerably faster: the average half-life of posts (i.e., the post age at which the cumulative ratio of reposts reaches to 50% of 36-hour reposts) amounted to merely 6.25 hours for posts with displayed notes (Figure S1c). For posts without displayed notes, the average half-life of posts is 5.75 hours, which is slightly but significantly ($t = 13.135, p < 0.001$) smaller than for posts with displayed notes. Figure S1d further shows that source posts with displayed notes tended to receive a higher cumulative number of 36-hour reposts (median of 432) on average, compared to those that did not have any displayed notes (median of 259, $KS = 0.117, p < 0.001$). Overall, these observations support earlier findings [7], indicating that community notes might be too slow to curb overall engagement with misleading

posts on X (formerly Twitter). Notwithstanding, even if community notes might be too slow to have a meaningful effect at the cumulative level, it is still entirely possible (and plausible) that they reduced reposts for posts with displayed notes relative to similar posts without displayed notes. Quantifying this intervention effect presents the main objective of our study.

The data further suggests that posts with displayed notes were more likely to be deleted on X (formerly Twitter) (see Figure S1d). On average, the deletion ratio was 17.3% for posts with displayed notes and 9.7% for posts without displayed notes ($t = 26.809, p < 0.001$).

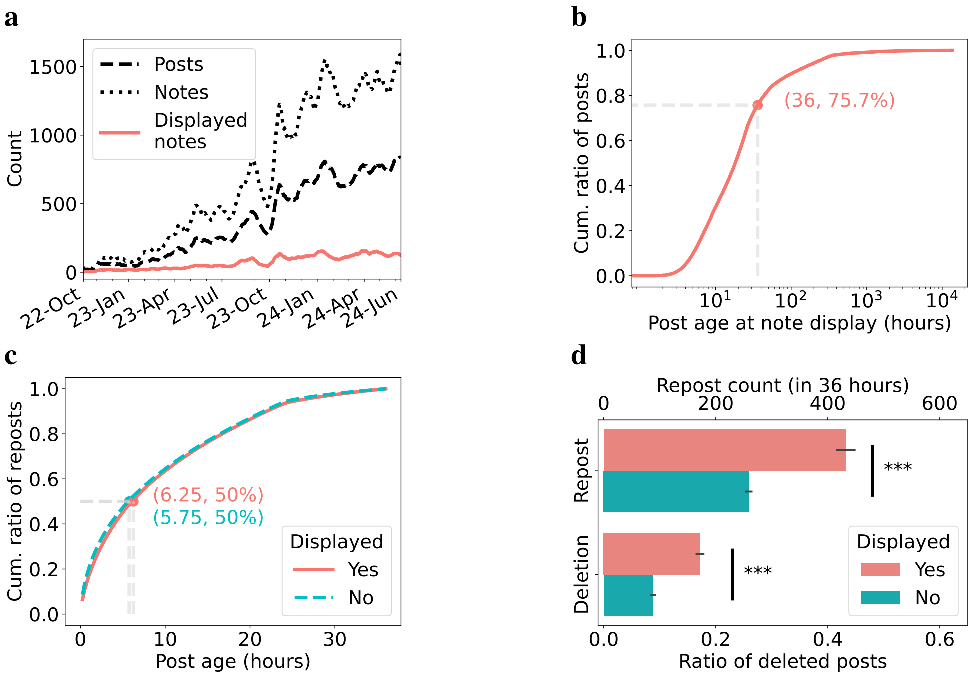

Figure S1: **Data overview.** **(a)** Two-week rolling averages of the daily counts of fact-checked source posts, community notes, and displayed notes in our observation period from October 6, 2022 to June 11, 2024. **(b)** The cumulative distribution of the ratios of posts that received displayed notes at different post ages relative to all posts with displayed notes. **(c)** The cumulative distribution of the ratios of reposts at different post ages relative to all reposts within 36 hours. **(d)** The repost counts of retrieved posts (upper half plot, shown are median values) and the ratios of deleted posts (bottom half plot, shown are mean values) within groups of posts with displayed notes and posts without displayed notes. The error bars represent 99% Confidence Intervals (CIs).

Supplementary Note 2: Descriptive Statistics

An overview of our dataset is reported in Table S1. In total, the data consists of 237,180 English source posts that were fact-checked between October 6, 2022 and June 11, 2024, i.e., during an observation period of approximately 20 months. These source posts were authored by 60,815 unique users and reposted more than 431 million times at the time of data collection.

We collected several variables to control for user characteristics that could potentially affect engagement with the posts. These variables include $\boldsymbol{Verified}$, which is a binary indicator showing whether the author is verified ($= 1$) or not ($= 0$). Additionally, $\boldsymbol{AccountAge}$ represents the age of the post account in days at the time of data collection. Furthermore, $\boldsymbol{Followers}$ denotes the number of followers for the post author, while $\boldsymbol{Followees}$ indicates the number of followees for the post author.

Given that the engagement with posts might change following the development of "Community Notes" program, we controlled for the time length from the months in which the posts were created to the roll-out of the program, i.e., Months From the Roll-Out ($\boldsymbol{MFRO}$). Additionally, we extracted a wide range of content characteristics that may affect engagement with the source posts. These characteristics include $\boldsymbol{Media}$, a binary variable indicating whether the post contains media elements ($= 1$) or not ($= 0$). Moreover, $\boldsymbol{Words}$ represents the word count in the source post. To determine the number of words in the source post, we performed text preprocessing by removing user mentions from the beginning of each text and eliminating all URLs. Furthermore, we converted HTML entities to Unicode characters and applied general International Components for Unicode (ICU) transforms [8] to further normalize the character set. We then used ICU BreakIterators to identify word boundaries for splitting the texts before counting the number of tokens containing alphanumeric characters. Additionally, we computed sentiment scores for the posts in our dataset using the Twitter-RoBERTa-base model, which has been fine-tuned for sentiment analysis on tweets and achieved a high performance compared to other models [9]. We use this

pre-trained machine learning model to calculate the probabilities that a given post conveys positive emotion (***Positive***) and negative emotion (***Negative***).

Ultimately, we performed topic modeling to study heterogeneity across topics. Specifically, we employed (and validated) a supervised machine learning framework (see details in Supplementary Note 3) to categorize the source tweets into predefined topics: (i) ***Politics***, (ii) ***Health***, (iii) ***Economy***, (iv) ***Science***. Posts that did do not fall into one of these topic categories were categorized as OTHER.

Table S1: Dataset overview. Descriptive statistics are reported for the whole dataset (column (1)) and the subsets of posts that have received (column (2)) or have not received a displayed helpful note during our observation period (column (3)). Continuous values are reported as means with standard deviations in parentheses, unless otherwise stated.

		Posts with displayed notes	
	All	Yes	No
#Reposts	431,360,739	64,661,218	366,699,521
#Posts	237,180	36,082	201,098
#Posters	60,815	13,858	55,221
Post date	10/06/2022 – 06/11/2024	10/06/2022 – 06/10/2024	10/06/2022 – 06/11/2024
Note date	10/06/2022 – 07/13/2024	10/06/2022 – 07/11/2024	10/06/2022 – 07/13/2024
Poster characteristics			
Verified	75.5 %	76.3 %	75.3 %
AccountAge (days)	2,957.457 (1,888.851)	2,730.113 (1,845.222)	2,998.249 (1,893.691)
Followers	2,324,391 (15,269,601)	1,514,229 (10,906,328)	2,469,754 (15,922,158)
Followees	6,858 (34,154)	7,269 (29,461)	6,784 (34,928)
Post characteristics			
Words	26.030 (14.888)	23.370 (14.585)	26.508 (14.891)
Media	63.6 %	72.0 %	62.1 %
Sentiment: *Positive*	0.155 (0.267)	0.167 (0.275)	0.152 (0.265)
Sentiment: *Negative*	0.429 (0.341)	0.396 (0.338)	0.435 (0.342)
Topic: *Economy*	13.0 %	12.3 %	13.1 %
Topic: *Health*	10.8 %	9.7 %	11.0 %
Topic: *Politics*	31.9 %	25.0 %	33.1 %
Topic: *Science*	11.1 %	13.8 %	10.7 %
Topic: *Other*	45.7 %	49.5 %	45.1 %
MFRO	14.236 (4.472)	13.654 (4.803)	14.340 (4.402)

2.1 Fact-Checking Activity Over Time

With the development of "Community Notes" feature on X (formerly Twitter), the absolute number of fact-checking notes and fact-checked posts increased significantly (see Figure S1a). However, the ratio of displayed notes to all community notes over time did not increase and even decreased after January of 2023 (Figure S2a). Notably, the ratio of displayed notes was relatively stable around 0.1 over the period after April of 2023. On average, 12.1% community notes were displayed every day, compared to the total notes that were created on the same day (Figure S2b).

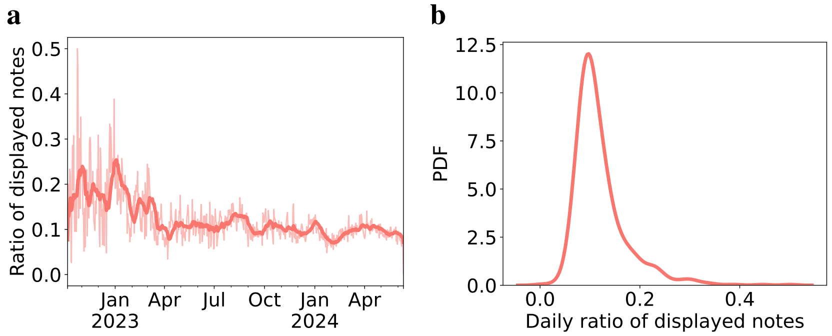

Figure S2: **The ratio of displayed notes relative to all the community notes over time.** **(a)** Two-week rolling averages of the ratio of displayed notes over time. **(b)** The Probability Density Function (PDF) of daily ratio of displayed notes.

2.2 Stability of Note Status

X (formerly Twitter) updates the status of notes on an hourly base according to its note ranking algorithm [10]. The rating algorithm considers all the ratings made by contributors from different perspectives to calculate the helpfulness score for each community note, determining its status as "Currently Rated Helpful," "Needs More Ratings," or "Currently Rated Not Helpful." Notably, contributors can also write notes that claim the source posts are not misleading. However, such kind of notes will stay at the status of "Needs More Ratings" despite of their high helpfulness scores. Community notes that carry the status of "Currently Rated Helpful" are directly displayed on the corresponding potentially misleading posts to inform users. On the other hand, community notes carrying the status of "Needs More Ratings" or "Currently Rated Not Helpful" are exclusively visible to the Community Notes contributors and, as such, are not expected to exert a substantial impact on the spread of associated source posts.

Out of the total notes, 368,910 (83.3%) remained in "Needs More Ratings (NMR)" status without any changes. 29,207 (6.6%) notes were initially rated not helpful in their first NonNMR status. Only 44,675 (10.1%) notes were initially rated helpful in their first NonNMR status with exact timestamps, and of them, 69.8% maintained helpful status in their current status and most recent status, while others changed to NMR in their current status. No note was rated helpful first and then changed into the status of not helpful in this dataset. In our main analysis, we used the first NonNMR status to indicate whether notes were displayed. Additionally, we conducted sensitivity analysis on the stability of helpful notes and found that stable helpful notes had a larger efficacy than non-stable helpful notes (see Supplementary Note 7.3).

Supplementary Note 3: Identification of Topics

We used machine learning to assign topic labels to the post in our dataset. Specifically, we employed (and validated) a supervised machine learning framework to categorize the source tweets into predefined topics: (i) POLITICS, (ii) HEALTH, (iii) ECONOMY, and (iv) SCIENCE. These topics have been identified based on a manual assessment of the posts in our dataset and the selection of topics in previous works (e.g., [11]). Posts that did do not fall into one of these topic categories were categorized as OTHER.

Our supervised machine learning framework proceeded as follows: First, we used a pre-trained large language model as the basis for our classifier, namely, the TwHIN-BERT (large) model [12]. This model was pre-trained on a corpus of 7 billion posts from X (formerly Twitter). Its training methodology incorporates not only text-based self-supervision (e.g., masked language model) but also a social objective derived from the social engagements with posts, thereby enhancing the model's understanding of social contexts. Second, we fine-tuned the model to our task using a manually labeled subset of posts from our dataset. Specifically, we tasked two trained research assistants to assign topic labels to a random subset of 1,500 community fact-checked posts, with each assistant labeling 750 distinct posts. To ensure label accuracy, both assistants also labeled an additional 175 posts that the other had already labeled. The inter-rater reliability assessment resulted in a relatively high macro averaged Cohens's κ coefficient of 0.711 and an overall agreement of 90.8 %. Finally, we used the labeled posts to train a deep neural network classifier to predict topic labels for all posts in our dataset. All hyperparameters were tuned using 10-fold cross validation. The model was implemented in Python 3.11.3 using the Transformers Python library (version 4.30.2; [13]).

The out-of-sample prediction performance (calculated using 10-fold cross-validation) of the classifier on the manually labeled posts is reported in Table S2. The classifier shows a high accuracy of, on average, 0.904; and a macro-averaged F_1 score of 0.755.

Table S2: Out-of-sample performance in topic prediction. Performance metrics were calculated using 10-fold cross-validation.

	Accuracy	F_1
ECONOMY	0.916	0.733
HEALTH	0.944	0.845
POLITICS	0.844	0.816
SCIENCE	0.912	0.628
[Macro-]Average	0.904	0.755

Supplementary Note 4: Propensity Score Matching

To reduce confounding and balance treatment and control groups, especially with respect to unmeasured confounders related to real-world events and selection bias, we performed one-to-one propensity score matching [14] to construct a control group from the source posts without displayed notes for the source posts with displayed notes. Specifically, we trained a logistic regression model based on the user-level and post-level variables (see Table S1) to estimate the propensity scores of all source posts. All continuous variables were z-standardized for better model fitting. According to the propensity score, we matched the closest post (caliper=0.15) in the group without displayed notes for each post in the treatment group without replacement, resulting in the creation of a matched control group for the treatment group. Additionally, we excluded matches from the source post without displayed notes in the first matching and performed the one-to-one propensity score matching again to construct another control group for the treatment group. We considered this second control group as placebo group that also shares similar user and post characteristics with the treatment group. The time when the community notes became displayed in control and placebo groups was assigned according to the corresponding posts in the treatment group. The placebo group can serve as a replacement for the treatment group in the comparison with the control group to demonstrate that there is no additional reduction in reposts without the actual treatment of note display.

As shown in Figure S3, the distributions of propensity score between source posts with displayed notes and source posts without displayed notes are much more balanced after matching compared to before matching. We tested the performance of the propensity score matching using binary logistic regressions. The binary dependent variable in the logistic regressions is $Display$ indicating whether the source posts have displayed community notes ($=1$) or not ($=0$). The explanatory variables are the user-level and post-level variables from Table S1. Column (1) of Table S3 reports the regression results for the source posts with and without displayed notes before the

matching. Many of the independent variables (e.g., $\mathit{Verified}$, $\mathit{AccountAge}$, $\mathit{Followers}$, and Media) are significantly different between the source posts with and without displayed notes. However, after the matching, all the independent variables among the treatment, control, and placebo groups are well-balanced and have no statistically significant differences (Columns (2) to (4) in Table S3).

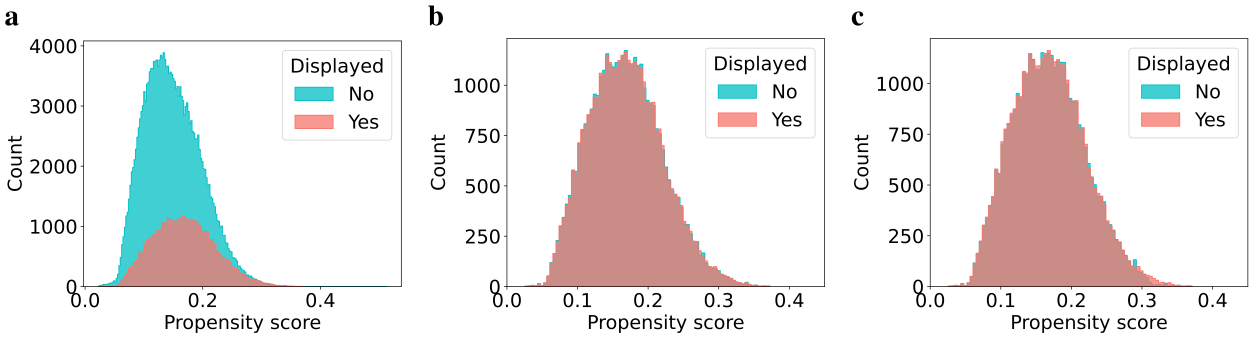

Figure S3: **The distributions of propensity score before and after one-to-one propensity score matching.** **(a)** The distributions of propensity score in the source posts with displayed notes and source posts without displayed notes before matching. **(b)** The distributions of propensity score in the source posts within the treatment group and matched source posts within the control group. **(c)** The distributions of peopensity score in the source posts within the treatment group and matched sorce posts within the placebo group.

Table S3: Regression results for the evaluation of propensity score matching. The binary dependent variable is $Display$. Column (1) reports the results for all the source posts before matching. Column (2) reports the results for the source posts in the treatment and control groups. Column (3) reports the results for the source posts in the treatment and placebo groups. Column (4) reports the results for the source posts in the placebo and control groups. Reported are coefficient estimates with standard errors in parentheses. * $p < 0.01$, ** $p < 0.005$, *** $p < 0.001$. Exact z statistics, p values and 99% CIs are reported in Table S4.

	(1) Before matching	(2) Treatment : Control	(3) Treatment : Placebo	(4) Placebo : Control
Verified	0.128***	0.010	–0.012	0.021
	(0.014)	(0.018)	(0.018)	(0.018)
AccountAge	–0.085***	0.002	–0.001	0.003
	(0.006)	(0.008)	(0.008)	(0.008)
Followers	–0.084***	–0.012	–0.020	0.008
	(0.009)	(0.011)	(0.010)	(0.010)
Followees	0.026***	0.006	–0.004	0.008
	(0.005)	(0.008)	(0.007)	(0.007)
Words	–0.164***	0.005	–0.006	0.012
	(0.006)	(0.008)	(0.008)	(0.008)
Media	0.358***	0.007	0.021	–0.015
	(0.013)	(0.017)	(0.017)	(0.017)
Economy	0.016	–0.016	–0.007	–0.008
	(0.018)	(0.023)	(0.023)	(0.023)
Health	–0.096***	–0.010	0.026	–0.036
	(0.020)	(0.026)	(0.026)	(0.026)
Politics	–0.261***	–0.008	–0.002	–0.006
	(0.014)	(0.019)	(0.019)	(0.019)
Science	0.188***	–0.035	–0.009	–0.027
	(0.018)	(0.023)	(0.023)	(0.023)
Positive	–0.023***	0.006	0.004	0.002
	(0.007)	(0.009)	(0.009)	(0.009)
Negative	–0.041***	0.000	0.015	–0.015
	(0.007)	(0.010)	(0.010)	(0.010)
MFRO	–0.186***	0.000	–0.011	0.012
	(0.006)	(0.007)	(0.007)	(0.007)
Intercept	–2.025***	–0.002	–0.004	0.003
	(0.016)	(0.021)	(0.021)	(0.021)
#Posts	237,175	72,154	71,984	71,984

Table S4: Details of z statistics, p values and 99% CIs (in brackets) for the coefficient estimates reported in Table S3.

	(1) Before matching	(2) Treatment : Control	(3) Treatment : Placebo	(4) Placebo : Control
Verified	z =9.21, p < 0.001	z =0.53, p =0.594	z =–0.66, p =0.512	z =1.16, p =0.247
	[0.092, 0.163]	[–0.037, 0.056]	[–0.058, 0.035]	[–0.025, 0.067]
AccountAge	z =–14.23, p < 0.001	z =0.25, p =0.805	z =–0.10, p =0.919	z =0.34, p =0.733
	[–0.101, –0.070]	[–0.018, 0.022]	[–0.021, 0.019]	[–0.017, 0.023]
Followers	z =–9.31, p < 0.001	z =–1.12, p =0.265	z =–1.90, p =0.057	z =0.81, p =0.419
	[–0.107, –0.061]	[–0.039, 0.016]	[–0.047, 0.007]	[–0.018, 0.035]
Followees	z =5.43, p < 0.001	z =0.79, p =0.429	z =–0.48, p =0.632	z =1.12, p =0.261
	[0.014, 0.038]	[–0.014, 0.027]	[–0.023, 0.016]	[–0.010, 0.026]
Words	z =–26.09, p < 0.001	z =0.63, p =0.526	z =–0.78, p =0.437	z =1.47, p =0.142
	[–0.181, –0.148]	[–0.016, 0.026]	[–0.027, 0.015]	[–0.009, 0.033]
Media	z =27.22, p < 0.001	z =0.39, p =0.697	z =1.24, p =0.214	z =–0.87, p =0.386
	[0.324, 0.392]	[–0.038, 0.051]	[–0.023, 0.066]	[–0.059, 0.029]
Economy	z =0.90, p =0.367	z =–0.67, p =0.503	z =–0.31, p =0.758	z =–0.34, p =0.738
	[–0.030, 0.063]	[–0.076, 0.044]	[–0.067, 0.053]	[–0.068, 0.052]
Health	z =–4.86, p < 0.001	z =–0.37, p =0.708	z =1.01, p =0.312	z =–1.38, p =0.168
	[–0.147, –0.045]	[–0.076, 0.056]	[–0.040, 0.093]	[–0.102, 0.031]
Politics	z =–18.48, p < 0.001	z =–0.45, p =0.650	z =–0.12, p =0.905	z =–0.35, p =0.727
	[–0.297, –0.224]	[–0.056, 0.039]	[–0.050, 0.046]	[–0.054, 0.041]
Science	z =10.46, p < 0.001	z =–1.57, p =0.116	z =–0.38, p =0.702	z =–1.21, p =0.226
	[0.141, 0.234]	[–0.093, 0.023]	[–0.067, 0.050]	[–0.085, 0.031]
Positive	z =–3.31, p < 0.001	z =0.68, p =0.498	z =0.41, p =0.681	z =0.22, p =0.829
	[–0.042, –0.005]	[–0.017, 0.030]	[–0.020, 0.027]	[–0.021, 0.025]
Negative	z =–5.53, p < 0.001	z =0.05, p =0.959	z =1.58, p =0.115	z =–1.61, p =0.107
	[–0.060, –0.022]	[–0.024, 0.025]	[–0.010, 0.040]	[–0.040, 0.009]
MFRO	z =–31.70, p < 0.001	z =0.01, p =0.992	z =–1.55, p =0.121	z =1.60, p =0.110
	[–0.202, –0.171]	[–0.018, 0.018]	[–0.030, 0.007]	[–0.007, 0.031]
Intercept	z =–128.33, p < 0.001	z =–0.09, p =0.928	z =–0.18, p =0.859	z =0.12, p =0.901
	[–2.066, –1.984]	[–0.056, 0.052]	[–0.057, 0.050]	[–0.051, 0.056]
#Posts	237,175	72,154	71,984	71,984

Supplementary Note 5: Estimation Results

5.1 Two-Period ATTs

The regression results for the two-period ATT estimation are reported in Table S5. The before-display period is between −4 and −1 hours from the note display. The period between 1 and 12 hours from the note display was considered as the after-display period.

The results for the main regression are reported in Column (1) of Table S5. Furthermore, Column (2) reports the results for an extended regression model that incorporates user and post characteristics as additional explanatory variables. The coefficient estimate for the difference-in-difference term ($Display \times After$) is qualitatively identical across both models, which further validates the effectiveness of the propensity score matching.

Table S5: Regression results for two-period ATT estimation. Post-specific random effects are included. Reported are coefficient estimates with standard errors in parentheses. * $p < 0.01$, ** $p < 0.005$, *** $p < 0.001$. Exact z statistics, p values and 99% CIs are reported in Table S6.

	(1) Main	(2) Extended
Display	1.200***	1.161***
	(0.023)	(0.022)
After	0.106***	0.105***
	(0.004)	(0.004)
Display×After	–0.948***	–0.948***
	(0.004)	(0.004)
PostAge	–0.797***	–0.796***
	(0.002)	(0.002)
Verified		1.144***
		(0.027)
AccountAge		–0.110***
		(0.012)
Followers		0.326***
		(0.017)
Followees		0.116***
		(0.011)
Words		0.075***
		(0.012)
Media		0.774***
		(0.026)
Economy		–0.368***
		(0.034)
Health		0.297***
		(0.038)
Politics		0.198***
		(0.027)
Science		–0.581***
		(0.033)
Positive		–0.094***
		(0.014)
Negative		0.188***
		(0.014)
MFRO		–0.180***
		(0.011)
Intercept	1.293***	–0.071
	(0.017)	(0.033)
Post-level RE	✓	✓
#Observations	654,400	654,400
#Posts	40,900	40,900

Table S6: Details of z statistics, p values and 99% CIs (in brackets) for the coefficient estimates reported in Table S5.

	(1)<br>Main	(2)<br>Extended
Display	z =51.36, p < 0.001<br>[1.140, 1.260]	z =52.25, p < 0.001<br>[1.103, 1.218]
After	z =25.27, p < 0.001<br>[0.095, 0.117]	z =24.99, p < 0.001<br>[0.094, 0.116]
Display×After	z =−211.71, p < 0.001<br>[−0.959, −0.936]	z =−211.82, p < 0.001<br>[−0.960, −0.937]
PostAge	z =−324.07, p < 0.001<br>[−0.803, −0.791]	z =−323.90, p < 0.001<br>[−0.802, −0.789]
Verified		z =42.27, p < 0.001<br>[1.074, 1.213]
AccountAge		z =−9.49, p < 0.001<br>[−0.140, −0.080]
Followers		z =19.06, p < 0.001<br>[0.282, 0.370]
Followees		z =10.26, p < 0.001<br>[0.087, 0.145]
Words		z =6.27, p < 0.001<br>[0.044, 0.106]
Media		z =30.12, p < 0.001<br>[0.708, 0.840]
Economy		z =−10.69, p < 0.001<br>[−0.457, −0.279]
Health		z =7.82, p < 0.001<br>[0.199, 0.395]
Politics		z =7.28, p < 0.001<br>[0.128, 0.268]
Science		z =−17.37, p < 0.001<br>[−0.667, −0.495]
Positive		z =−6.85, p < 0.001<br>[−0.129, −0.059]
Negative		z =13.37, p < 0.001<br>[0.152, 0.224]
MFRO		z =−16.47, p < 0.001<br>[−0.208, −0.152]
Intercept	z =77.92, p < 0.001<br>[1.251, 1.336]	z =−2.15, p =0.032<br>[−0.156, 0.014]
Post-level RE	✓	✓
#Observations	654,400	654,400
#Posts	40,900	40,900

5.2 Parallel Test and Multi-Period ATTs

In this section, we present the estimation results of our multi-period DiD analysis that allows us to examine the treatment effect over time. We begin by assessing parallel trends to validate the applicability of the DiD framework (Supplementary Note 5.2.1). Subsequently, we perform a comprehensive series of robustness checks and sensitivity analyses to further confirm the reliability of our estimates (Supplementary Note 5.2.2).

5.2.1 Estimation results and parallel trends

The regression results for the parallel test and multi-period ATTs are reported in Table S7. The first hour preceding the display of community notes is the baseline period in the multi-period model. The coefficient estimates of the difference-in-differences terms for the three hourly before-display periods are used to assess parallel trends relative to the baseline period. The coefficient estimates of the difference-in-differences terms in the hourly after-display periods are used to estimate hourly multi-period ATTs relative to the baseline period.

The parallel trends assumption in DiD models requires that, in the absence of treatment, the untreated potential outcomes of the treatment and control groups would follow the same trajectory. A common approach to testing this assumption is to set a null hypothesis that no pre-treatment differences exist and interpret failure to reject the null hypothesis as evidence that parallel trends hold. However, prior research suggests this approach can be misleading, especially in large datasets where statistical power is high [15, 16, 17, 18]. In such cases, even small pre-treatment differences can appear statistically significant, leading to the (incorrect) conclusion that the DiD framework is not applicable. To address this, the DiD literature proposes "non-inferiority" approaches, which reverse the null hypothesis: instead of testing whether pre-treatment differences are exactly zero, it tests whether these differences exceed a meaningful threshold [15, 16, 17]. If the data provide strong evidence that pre-trends are small, the null hypothesis of a "large pre-treatment difference"

is rejected, supporting the validity of the DiD design. In the following, we (i) first assess parallel trends using conventional null hypothesis significance testing. Subsequently, we (ii) then apply equivalence testing to determine whether any detected pre-trends are practically meaningful. In addition, we (iii) use alternative baseline periods to evaluate the sensitivity of parallel trends.

Null hypothesis testing: The coefficient estimates for $\mathit{Display} \times \mathit{Before} : \mathit{4}$ $(\mathit{coef.} =$ 0.040, p <0.001), $\mathit{Display} \times \mathit{Before} : \mathit{3}$ $(\mathit{coef.} =$ 0.043, p <0.001) are statistically significant at the 1% statistical significance level, whereas the coefficient estimate for $\mathit{Display} \times \mathit{Before} : \mathit{2}$ $(\mathit{coef.} =$ 0.026, p =0.011) is statistically significant at the 5% statistical significance level. This indicates the presence of pre-trend differences between treatment and control groups. However, compared to the post-treatment effects, the effect sizes are small, making it unclear whether these differences are meaningful or merely a consequence of high statistical power. To determine whether these pre-trends are practically negligible, we conduct equivalence testing in the following.

Equivalence testing: We follow the DiD literature [15, 16] by considering an alternative null hypothesis of "non-negligible trend differences" and conducting a test for statistical equivalence by specifying a lower and upper bound. If the differences fall within the equivalence bounds, they are considered equivalent to the absence of pre-trends that are worthwhile to examine. Accordingly, we performed an equivalence test to assess whether the estimated trend differences between treatment and control groups during before-display period were large enough to be considered meaningful. As suggested in [19], the differences of 0.3 scale point on a 5-point scale can be considered as a reasonable equivalence bound. Given this, we define the bound for our equivalence test as:

$$\Delta = 0.3 * \delta_{max}/5, \tag{1}$$

where δ_{max} is the max absolute value of the estimated coefficients of difference-in-differences terms for hourly periods before and after the display of community notes. In our data, the value of δ_{max} is 1.148, and the equivalence bound is 0.069.

As shown in Figure S4, all the DiD coefficient estimates during before-display period fall within the range of equivalence bounds. This means that, while statistically significant, the small trend differences during before-display period between treatment and control groups are negligible and equivalent to the absence of pre-trends. In sum, this implies that the DiD framework is applicable in our study.

Parallel test with alternative baseline periods: Our main analysis uses the hour immediately preceding note display as the baseline. To assess the sensitivity of our findings to this choice, we re-estimated the DiD model using alternative baselines, shifting the reference period to the second, third, and fourth before-display hours. Table S9 reports DiD coefficient estimates based on baseline periods from the second hour to the fourth hour preceding the display of community notes. In Column (1) of Table S9, the baseline period is the second hour before note display, and all the DiD coefficient estimates during before-display period are not statistically significant. Additionally, in Columns (2) and (3) of Table S9, only the coefficient estimates of $\mathit{Display} \times \mathit{Before} : \mathit{1}$ are statistically significant but fall within the range of equivalence bounds. This suggests that the trend differences between treatment and control groups are too small to be considered meaningful regardless of baseline periods.

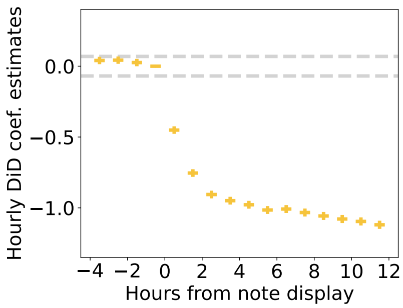

Figure S4: **Equivalence test for parallel trend.** Shown are hourly DiD coefficient estimates with 99% CIs. The two grey horizontal dash lines indicates the lower and upper bounds for the equivalence test.

Table S7: Regression results for parallel test and multi-period ATTs estimation. Post-specific random effects are included. Reported are coefficient estimates with standard errors in parentheses. * $p < 0.01$, ** $p < 0.005$, *** $p < 0.001$. Exact z statistics, p values and 99% CIs are reported in Table S8.

	Multi-period		Continued		Continued
Display	1.175***	Display×Before:4	0.040***	Intercept	1.397***
	(0.024)		(0.010)		(0.019)
Before:4	0.161***	Display×Before:3	0.043***	Post-level RE	✓
	(0.010)		(0.010)		
Before:3	0.055***	Display×Before:2	0.026	#Observations	654,400
	(0.009)		(0.010)	#Posts	40,900
Before:2	0.014	Display×After:1	–0.451***		
	(0.008)		(0.010)		
After:1	–0.010	Display×After:2	–0.754***		
	(0.008)		(0.010)		
After:2	–0.031***	Display×After:3	–0.906***		
	(0.009)		(0.011)		
After:3	–0.037***	Display×After:4	–0.949***		
	(0.010)		(0.011)		
After:4	–0.046***	Display×After:5	–0.978***		
	(0.011)		(0.011)		
After:5	–0.060***	Display×After:6	–1.015***		
	(0.013)		(0.011)		
After:6	–0.050***	Display×After:7	–1.008***		
	(0.015)		(0.011)		
After:7	–0.069***	Display×After:8	–1.033***		
	(0.016)		(0.011)		
After:8	–0.055**	Display×After:9	–1.057***		
	(0.018)		(0.011)		
After:9	–0.046	Display×After:10	–1.079***		
	(0.020)		(0.011)		
After:10	–0.038	Display×After:11	–1.096***		
	(0.022)		(0.011)		
After:11	–0.026	Display×After:12	–1.120***		
	(0.024)		(0.011)		
After:12	–0.006	PostAge	–0.603***		
	(0.026)		(0.015)		

Table S8: Details of z statistics, p values and 99% CIs (in brackets) for the coefficient estimates reported in Table S7

Multi-period		Continued		Continued	
Display	z =48.74, $p < 0.001$<br>[1.113, 1.237]	Display×Before:4	z =4.01, $p < 0.001$<br>[0.014, 0.066]	Intercept	z =72.05, $p < 0.001$<br>[1.347, 1.447]
Before:4	z =16.76, $p < 0.001$<br>[0.136, 0.186]	Display×Before:3	z =4.24, $p < 0.001$<br>[0.017, 0.069]	Post-level RE	✓
Before:3	z =6.42, $p < 0.001$<br>[0.033, 0.077]	Display×Before:2	z =2.55, p =0.011<br>[0.000, 0.052]	#Observations	654,400
Before:2	z =1.84, p =0.066<br>[−0.006, 0.035]	Display×After:1	z =−43.82, $p < 0.001$<br>[−0.477, −0.424]	#Posts	40,900
After:1	z =−1.26, p =0.209<br>[−0.031, 0.011]	Display×After:2	z =−72.23, $p < 0.001$<br>[−0.781, −0.727]		
After:2	z =−3.49, $p < 0.001$<br>[−0.054, −0.008]	Display×After:3	z =−85.81, $p < 0.001$<br>[−0.933, −0.879]		
After:3	z =−3.68, $p < 0.001$<br>[−0.063, −0.011]	Display×After:4	z =−89.27, $p < 0.001$<br>[−0.977, −0.922]		
After:4	z =−4.03, $p < 0.001$<br>[−0.076, −0.017]	Display×After:5	z =−91.34, $p < 0.001$<br>[−1.006, −0.951]		
After:5	z =−4.59, $p < 0.001$<br>[−0.093, −0.026]	Display×After:6	z =−94.17, $p < 0.001$<br>[−1.043, −0.987]		
After:6	z =−3.39, $p < 0.001$<br>[−0.087, −0.012]	Display×After:7	z =−93.00, $p < 0.001$<br>[−1.036, −0.980]		
After:7	z =−4.23, $p < 0.001$<br>[−0.111, −0.027]	Display×After:8	z =−94.71, $p < 0.001$<br>[−1.061, −1.005]		
After:8	z =−3.04, p =0.002<br>[−0.102, −0.008]	Display×After:9	z =−96.35, $p < 0.001$<br>[−1.086, −1.029]		
After:9	z =−2.30, p =0.022<br>[−0.097, 0.006]	Display×After:10	z =−97.66, $p < 0.001$<br>[−1.107, −1.050]		
After:10	z =−1.73, p =0.083<br>[−0.094, 0.018]	Display×After:11	z =−98.59, $p < 0.001$<br>[−1.124, −1.067]		
After:11	z =−1.09, p =0.276<br>[−0.087, 0.035]	Display×After:12	z =−100.01, $p < 0.001$<br>[−1.148, −1.091]		
After:12	z =−0.23, p =0.818<br>[−0.072, 0.060]	PostAge	z =−40.74, $p < 0.001$<br>[−0.641, −0.565]		

Table S9: Regression results parallel trends test with alternative baseline periods. Post-specific random effects are included. Reported are coefficient estimates with standard errors in parentheses. * $p < 0.01$, ** $p < 0.005$, *** $p < 0.001$. Exact z statistics, p values and 99% CIs are reported in Table S10.

	Base period: -2	Base period: -3	Base period: -4
Display×Before:4	0.015	–0.002	
	(0.010)	(0.010)	
Display×Before:3	0.017		0.002
	(0.010)		(0.010)
Display×Before:2		–0.017	–0.015
		(0.010)	(0.010)
Display×Before:1	–0.026	–0.043***	–0.040***
	(0.010)	(0.010)	(0.010)
Display×After:1	–0.477***	–0.494***	–0.491***
	(0.010)	(0.010)	(0.010)
Display×After:2	–0.780***	–0.797***	–0.795***
	(0.010)	(0.010)	(0.010)
Display×After:3	–0.932***	–0.949***	–0.946***
	(0.011)	(0.011)	(0.011)
Display×After:4	–0.975***	–0.992***	–0.990***
	(0.011)	(0.011)	(0.011)
Display×After:5	–1.004***	–1.021***	–1.019***
	(0.011)	(0.011)	(0.011)
Display×After:6	–1.041***	–1.058***	–1.056***
	(0.011)	(0.011)	(0.011)
Display×After:7	–1.034***	–1.051***	–1.049***
	(0.011)	(0.011)	(0.011)
Display×After:8	–1.059***	–1.076***	–1.073***
	(0.011)	(0.011)	(0.011)
Display×After:9	–1.083***	–1.100***	–1.098***
	(0.011)	(0.011)	(0.011)
Display×After:10	–1.105***	–1.122***	–1.119***
	(0.011)	(0.011)	(0.011)
Display×After:11	–1.122***	–1.139***	–1.136***
	(0.011)	(0.011)	(0.011)
Display×After:12	–1.145***	–1.162***	–1.160***
	(0.011)	(0.011)	(0.011)

Table S10: Details of z statistics, p values and 99% CIs (in brackets) for the coefficient estimates reported in Table S9.

	Base period: -2	Base period: -3	Base period: -4
Display×Before:4	z =1.46, p =0.145<br>[–0.011, 0.040]	z =–0.24, p =0.810<br>[–0.028, 0.023]	z =0.24, p =0.810<br>[–0.023, 0.028]
Display×Before:3	z =1.69, p =0.090<br>[–0.009, 0.043]	z =–1.69, p =0.090<br>[–0.043, 0.009]	z =–1.46, p =0.145<br>[–0.040, 0.011]
Display×Before:2	z =–2.55, p =0.011<br>[–0.052, 0.000]	z =–4.24, $p < 0.001$<br>[–0.069, –0.017]	z =–4.01, $p < 0.001$<br>[–0.066, –0.014]
Display×After:1	z =–46.42, $p < 0.001$<br>[–0.503, –0.450]	z =–48.18, $p < 0.001$<br>[–0.520, –0.467]	z =–47.95, $p < 0.001$<br>[–0.518, –0.465]
Display×After:2	z =–74.86, $p < 0.001$<br>[–0.807, –0.753]	z =–76.65, $p < 0.001$<br>[–0.824, –0.770]	z =–76.45, $p < 0.001$<br>[–0.821, –0.768]
Display×After:3	z =–88.44, $p < 0.001$<br>[–0.959, –0.905]	z =–90.26, $p < 0.001$<br>[–0.976, –0.922]	z =–90.08, $p < 0.001$<br>[–0.973, –0.919]
Display×After:4	z =–91.90, $p < 0.001$<br>[–1.003, –0.948]	z =–93.73, $p < 0.001$<br>[–1.019, –0.965]	z =–93.56, $p < 0.001$<br>[–1.017, –0.963]
Display×After:5	z =–93.97, $p < 0.001$<br>[–1.032, –0.976]	z =–95.80, $p < 0.001$<br>[–1.048, –0.994]	z =–95.65, $p < 0.001$<br>[–1.046, –0.991]
Display×After:6	z =–96.80, $p < 0.001$<br>[–1.069, –1.013]	z =–98.64, $p < 0.001$<br>[–1.086, –1.030]	z =–98.49, $p < 0.001$<br>[–1.083, –1.028]
Display×After:7	z =–95.62, $p < 0.001$<br>[–1.062, –1.006]	z =–97.45, $p < 0.001$<br>[–1.079, –1.023]	z =–97.29, $p < 0.001$<br>[–1.076, –1.021]
Display×After:8	z =–97.32, $p < 0.001$<br>[–1.087, –1.031]	z =–99.14, $p < 0.001$<br>[–1.104, –1.048]	z =–98.99, $p < 0.001$<br>[–1.101, –1.045]
Display×After:9	z =–98.95, $p < 0.001$<br>[–1.111, –1.055]	z =–100.77, $p < 0.001$<br>[–1.128, –1.072]	z =–100.62, $p < 0.001$<br>[–1.126, –1.070]
Display×After:10	z =–100.25, $p < 0.001$<br>[–1.133, –1.076]	z =–102.05, $p < 0.001$<br>[–1.150, –1.093]	z =–101.90, $p < 0.001$<br>[–1.147, –1.091]
Display×After:11	z =–101.15, $p < 0.001$<br>[–1.150, –1.093]	z =–102.94, $p < 0.001$<br>[–1.167, –1.110]	z =–102.80, $p < 0.001$<br>[–1.165, –1.108]
Display×After:12	z =–102.56, $p < 0.001$<br>[–1.174, –1.117]	z =–104.34, $p < 0.001$<br>[–1.191, –1.134]	z =–104.19, $p < 0.001$<br>[–1.189, –1.131]

5.2.2 *Sensitivity analyses for DiD estimates*

In addition to demonstrating that the pre-trends in our dataset are negligible, we followed best practices [16] and conducted a wide range of sensitivity analyses to further assess the robustness of our DiD estimates. These include (i) using bounds on relative magnitudes, (ii) incorporation of linear trend, and (iii) rolling regressions. In all of these checks, the findings consistently confirm the reliability of our estimates.

Bounds on relative magnitudes: To further assess the robustness of our estimates against potential violations of parallel trends, we employed HonestDiD using the Conditional Least Favorable Hybrid (C-LF) method (see [16, 20] for details). HonestDiD is a robust inference approach designed to quantify uncertainty in DiD estimates when strict parallel trends between treatment and control groups may not hold. Instead of assuming that any detected pre-trend invalidates the analysis, HonestDiD allows for small trend differences and adjusts the estimates accordingly. Specifically, the method assumes that any deviations from parallel trends in the after-display period cannot exceed $\overline{M}$ times the largest deviation observed in the before-display period. As shown in Figure S5, our DiD estimates remain robust throughout the 12 hours following the display of community notes, even when allowing after-display deviations to be twice as large as the maximum before-display deviation (i.e., $\overline{M} = 2$). These results confirm that our findings hold even under relaxed parallel trends assumptions, reinforcing the credibility of our estimates.

Incorporation of linear trend: To further account for potential pre-trends, we incorporated a linear trend adjustment into the DiD model, assuming that existing trends in the before-display period continue into the after-display period [15, 18]. Based on the estimation results with varying baseline periods (see Table S9), we found that the pre-trend in the hour immediately before note display slightly but significantly differed from that in the three preceding hours, while the pre-trends in those three preceding hours were parallel with each other. Given this, we used the slope between the second and first before-display hours as a linear pre-trend in an augmented DiD model. The full estimation results are reported in Table S11. The coefficient estimate of the slope is not

statistically significant ($coef. =$ –0.026, $p =$0.011). Additionally, Figure S6a shows that the hourly DiD coefficient estimates between the augmented DiD model with linear trend and the standard DiD model are not statistically significantly different from each other. Overall, this suggests that our results were robust to potential linear trends extending into the after-display period.

Rolling regressions: We conducted a series of rolling DiD regressions using a two-hour sliding window, covering the period from four hours before to twelve hours after note display. In each rolling DiD regression, we examined the difference between the treatment and control groups in the subsequent hour, relative to the previous hour. The results indicate a sharp drop in the rolling DiD coefficient immediately after note display, followed by a gradual increase that stabilizes by the fourth hour after note display (see Figure S6b). This suggests that the display of community notes significantly reduced the spread of misleading posts, with its effect stabilizing within four hours.

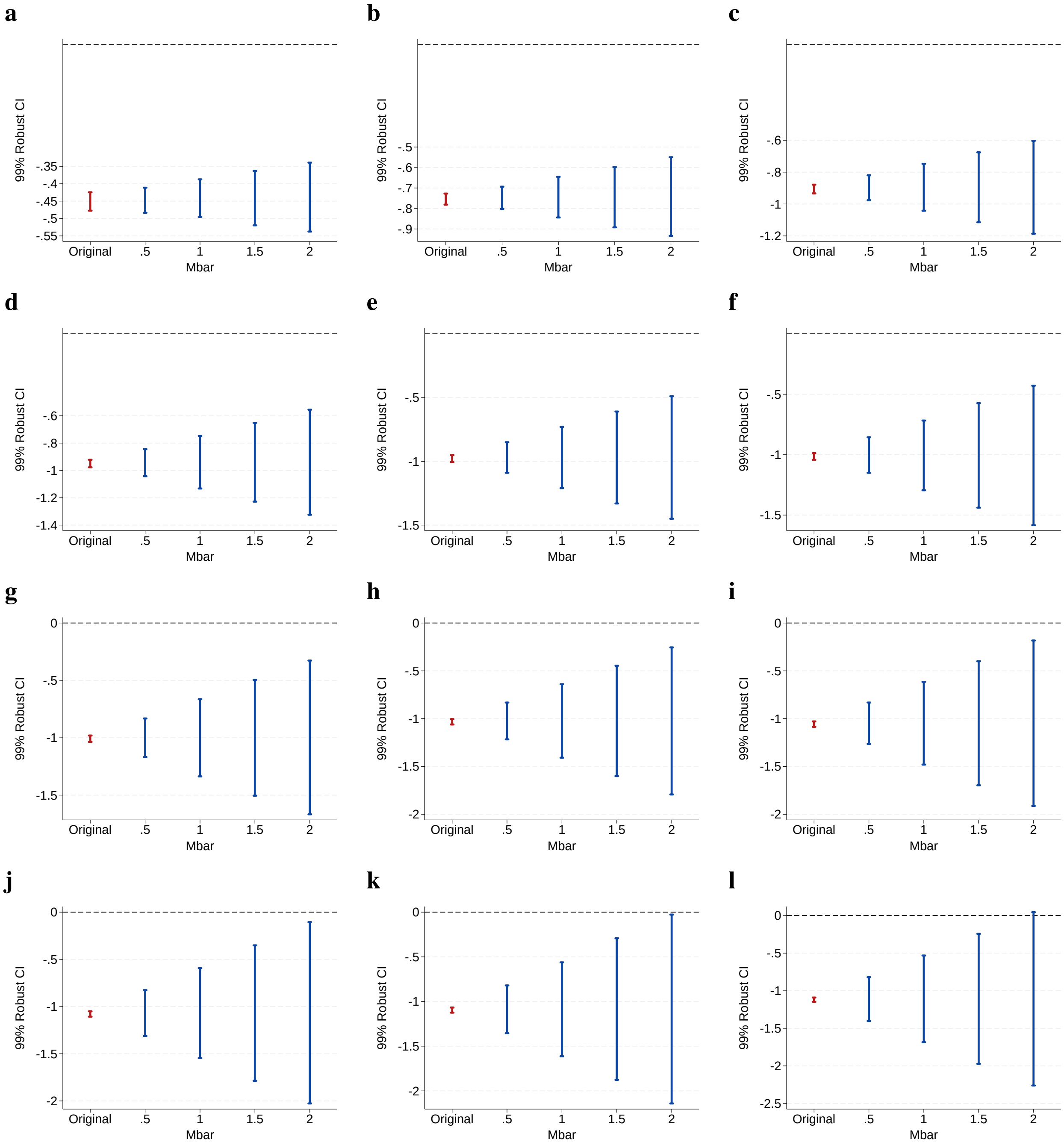

Figure S5: **The original DiD coefficient estimates and robust confidence intervals under different Mbar ($\overline{M}$) from 0.5 to 2 with an increment of 0.5. (a) – (l)** The first hour to the twelfth hour after the display of community notes. The error bars represent 99% CIs. The robust confidence intervals are interpreted based on HonestDiD with the C-LF method and relative magnitudes restrictions [20].

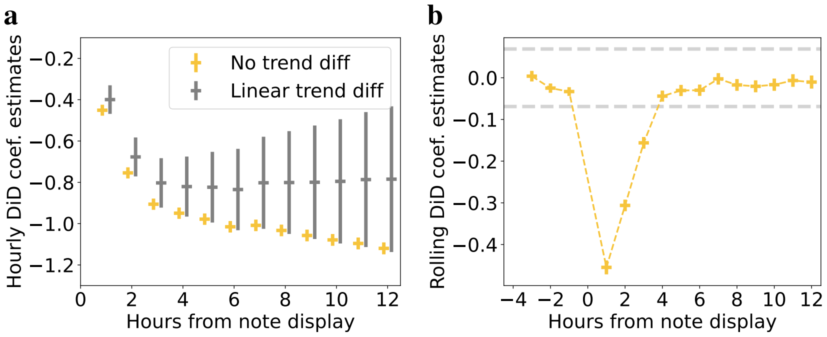

Figure S6: **The hourly DiD coefficient estimates.** **(a)** The hourly DiD coefficient estimates in an augmented DiD model with a linear trend. **(b)** The hourly DiD coefficient estimates using a rolling two-hour sliding window. The horizontal grey lines represent the upper and lower equivalence bounds. The error bars represent 99% CIs.

Table S11: Regression results for parallel test and multi-period ATTs estimation. Post-specific random effects are included. Reported are coefficient estimates with standard errors in parentheses. * $p < 0.01$, ** $p < 0.005$, *** $p < 0.001$. Exact z statistics, p values and 99% CIs are reported in Table S12.

	Multi-period		Continued		Continued
Display	1.149***	Display×Before:4	–0.037	Intercept	1.397***
	(0.028)		(0.027)		(0.019)
Before:4	0.161***	Display×Before:3	–0.009	Post-level RE	✓
	(0.010)		(0.017)		
Before:3	0.055***	Display×After:1	–0.399***	#Observations	654,400
	(0.009)		(0.027)	#Posts	40,900
Before:2	0.014	Display×After:2	–0.677***		
	(0.008)		(0.037)		
After:1	–0.010	Display×After:3	–0.803***		
	(0.008)		(0.046)		
After:2	–0.031***	Display×After:4	–0.821***		
	(0.009)		(0.056)		
After:3	–0.037***	Display×After:5	–0.824***		
	(0.010)		(0.066)		
After:4	–0.046***	Display×After:6	–0.835***		
	(0.011)		(0.076)		
After:5	–0.060***	Display×After:7	–0.802***		
	(0.013)		(0.086)		
After:6	–0.050***	Display×After:8	–0.801***		
	(0.015)		(0.096)		
After:7	–0.069***	Display×After:9	–0.800***		
	(0.016)		(0.106)		
After:8	–0.055**	Display×After:10	–0.795***		
	(0.018)		(0.117)		
After:9	–0.046	Display×After:11	–0.787***		
	(0.020)		(0.127)		
After:10	–0.038	Display×After:12	–0.785***		
	(0.022)		(0.137)		
After:11	–0.026	DiD Slope	–0.026		
	(0.024)		(0.010)		
After:12	–0.006	PostAge	–0.603***		
	(0.026)		(0.015)		

Table S12: Details of z statistics, p values and 99% CIs (in brackets) for the coefficient estimates reported in Table S11.

Multi-period		Continued		Continued	
Display	z =40.99, $p < 0.001$ [1.077, 1.221]	Display×Before:4	z =−1.39, p =0.166 [−0.106, 0.032]	Intercept	z =72.05, $p < 0.001$ [1.347, 1.447]
Before:4	z =16.76, $p < 0.001$ [0.136, 0.186]	Display×Before:3	z =−0.50, p =0.614 [−0.054, 0.036]	Post-level RE	✓
Before:3	z =6.42, $p < 0.001$ [0.033, 0.077]	Display×After:1	z =−14.90, $p < 0.001$ [−0.468, −0.330]	#Observations	654,400
Before:2	z =1.84, p =0.066 [−0.006, 0.035]	Display×After:2	z =−18.54, $p < 0.001$ [−0.771, −0.583]	#Posts	40,900
After:1	z =−1.26, p =0.209 [−0.031, 0.011]	Display×After:3	z =−17.30, $p < 0.001$ [−0.922, −0.683]		
After:2	z =−3.49, $p < 0.001$ [−0.054, −0.008]	Display×After:4	z =−14.56, $p < 0.001$ [−0.966, −0.675]		
After:3	z =−3.68, $p < 0.001$ [−0.063, −0.011]	Display×After:5	z =−12.42, $p < 0.001$ [−0.995, −0.653]		
After:4	z =−4.03, $p < 0.001$ [−0.076, −0.017]	Display×After:6	z =−10.93, $p < 0.001$ [−1.032, −0.638]		
After:5	z =−4.59, $p < 0.001$ [−0.093, −0.026]	Display×After:7	z =−9.29, $p < 0.001$ [−1.025, −0.580]		
After:6	z =−3.39, $p < 0.001$ [−0.087, −0.012]	Display×After:8	z =−8.31, $p < 0.001$ [−1.049, −0.553]		
After:7	z =−4.23, $p < 0.001$ [−0.111, −0.027]	Display×After:9	z =−7.51, $p < 0.001$ [−1.074, −0.525]		
After:8	z =−3.04, p =0.002 [−0.102, −0.008]	Display×After:10	z =−6.82, $p < 0.001$ [−1.096, −0.495]		
After:9	z =−2.30, p =0.022 [−0.097, 0.006]	Display×After:11	z =−6.21, $p < 0.001$ [−1.113, −0.460]		
After:10	z =−1.73, p =0.083 [−0.094, 0.018]	Display×After:12	z =−5.74, $p < 0.001$ [−1.137, −0.433]		
After:11	z =−1.09, p =0.276 [−0.087, 0.035]	DiD Slope	z =−2.55, p =0.011 [−0.052, 0.000]		
After:12	z =−0.23, p =0.818 [−0.072, 0.060]	PostAge	z =−40.74, $p < 0.001$ [−0.641, −0.565]		

Supplementary Note 6: Placebo Analyses

We performed one-to-one matching again for the source posts in the treatment group and constructed a placebo group from source posts without displayed notes based on their propensity scores (see Supplementary Note 4). The placebo group demonstrates no statistically significant difference with the treatment group across the variables from user profiles and post features (Table S3). We considered the placebo group as a virtual treatment group that is similar to the treatment group but did not receive actual treatment (i.e., note display). Subsequently, we investigated whether there was an additional reduction in reposts for placebo group during the after-display period compared to control group and relative to the before-display period. As shown in Figure S7a, the changes in reposts within the placebo group overlapped with those within the control group. Based on the results from the regression that estimates multi-period ATTs using placebo and control groups (see Table S13), we found that all the hourly ATT estimations, whether before or after the note display, were not statistically significant. As visualized in Figure S7b, there is no significant extra reductions after the display of community notes. Additionally, the two-period ATT estimation with the replacement of the actual treatment group was not statistically significant (ATT: 0.002; 99% CI: [–0.010, 0.013]; $z = 0.38$, $p = 0.708$; see Table S15). These results suggest that the spread of the source posts in placebo and control groups followed a parallel pattern regardless of the note display.

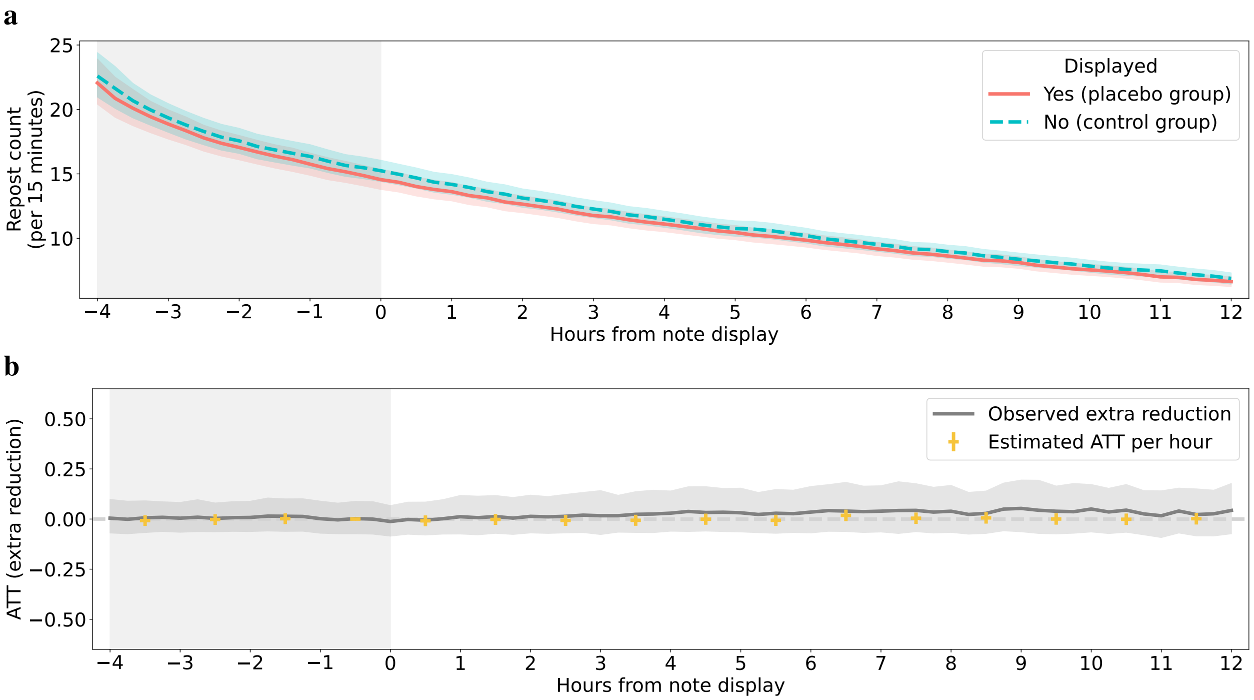

Figure S7: **Placebo analyses for the effectiveness of community notes. (a)** The changes in repost count within the source posts in the placebo and control groups. The error bands represent 99% CIs. **(b)** The estimated hourly extra reductions (ATTs) of reposts in placebo group after the display of community notes, compared to control group and relative to before-display period (yellow). The error bars represent 99% CIs. The grey belt (with 99% CIs) visualizes the observed extra reduction of the ratio of reposts in the treatment group relative to reposts in the control group and compared to the ratio of reposts before the display of community notes.

Table S13: Regression results for the multi-period ATT estimation with the replacement of treatment group. Post-specific random effects are included. Reported are coefficient estimates with standard errors in parentheses. * $p < 0.01$, ** $p < 0.005$, *** $p < 0.001$. Exact z statistics, p values and 99% CIs are reported in Table S14.

	Multi-period		Continued		Continued
Display	–0.058	Display×Before:4	–0.008	Intercept	1.317***
	(0.027)		(0.010)		(0.021)
Before:4	0.134***	Display×Before:3	–0.002	Post-level RE	✓
	(0.010)		(0.010)		
Before:3	0.037***	Display×Before:2	0.002	#Observations	652,976
	(0.009)		(0.010)	#Posts	40,811
Before:2	0.005	Display×After:1	–0.009		
	(0.008)		(0.011)		
After:1	0.001	Display×After:2	–0.002		
	(0.008)		(0.011)		
After:2	–0.011	Display×After:3	–0.007		
	(0.009)		(0.011)		
After:3	–0.008	Display×After:4	–0.006		
	(0.010)		(0.011)		
After:4	–0.008	Display×After:5	–0.001		
	(0.012)		(0.011)		
After:5	–0.012	Display×After:6	–0.006		
	(0.014)		(0.011)		
After:6	0.006	Display×After:7	0.018		
	(0.016)		(0.011)		
After:7	–0.004	Display×After:8	0.004		
	(0.018)		(0.011)		
After:8	0.019	Display×After:9	0.006		
	(0.020)		(0.011)		
After:9	0.038	Display×After:10	0.000		
	(0.022)		(0.011)		
After:10	0.054	Display×After:11	–0.001		
	(0.024)		(0.011)		
After:11	0.075**	Display×After:12	0.002		
	(0.026)		(0.011)		
After:12	0.104***	PostAge	–0.670***		
	(0.028)		(0.016)		

Table S14: Details of z statistics, p values and 99% CIs (in brackets) for the coefficient estimates reported in Table S13.

Multi-period		Continued		Continued	
Display	z =–2.16, p =0.031 [–0.127, 0.011]	Display×Before:4	z =–0.74, p =0.461 [–0.034, 0.019]	Intercept	z =61.49, $p < 0.001$ [1.262, 1.372]
Before:4	z =13.60, $p < 0.001$ [0.109, 0.160]	Display×Before:3	z =–0.24, p =0.814 [–0.029, 0.024]	Post-level RE	✓
Before:3	z =4.33, $p < 0.001$ [0.015, 0.059]	Display×Before:2	z =0.17, p =0.866 [–0.025, 0.029]	#Observations	652,976
Before:2	z =0.71, p =0.477 [–0.014, 0.025]	Display×After:1	z =–0.83, p =0.405 [–0.036, 0.018]	#Posts	40,811
After:1	z =0.13, p =0.897 [–0.019, 0.021]	Display×After:2	z =–0.18, p =0.855 [–0.029, 0.025]		
After:2	z =–1.22, p =0.223 [–0.034, 0.012]	Display×After:3	z =–0.64, p =0.524 [–0.034, 0.021]		
After:3	z =–0.75, p =0.452 [–0.035, 0.019]	Display×After:4	z =–0.57, p =0.572 [–0.034, 0.022]		
After:4	z =–0.67, p =0.506 [–0.039, 0.023]	Display×After:5	z =–0.06, p =0.952 [–0.028, 0.027]		
After:5	z =–0.89, p =0.371 [–0.048, 0.023]	Display×After:6	z =–0.58, p =0.563 [–0.034, 0.022]		
After:6	z =0.41, p =0.683 [–0.034, 0.047]	Display×After:7	z =1.66, p =0.097 [–0.010, 0.046]		
After:7	z =–0.21, p =0.833 [–0.049, 0.042]	Display×After:8	z =0.38, p =0.704 [–0.024, 0.032]		
After:8	z =0.98, p =0.329 [–0.032, 0.070]	Display×After:9	z =0.53, p =0.598 [–0.023, 0.034]		
After:9	z =1.72, p =0.085 [–0.019, 0.094]	Display×After:10	z =0.03, p =0.972 [–0.028, 0.029]		
After:10	z =2.28, p =0.022 [–0.007, 0.116]	Display×After:11	z =–0.10, p =0.922 [–0.030, 0.028]		
After:11	z =2.90, p =0.004 [0.008, 0.142]	Display×After:12	z =0.15, p =0.878 [–0.027, 0.031]		
After:12	z =3.70, $p < 0.001$ [0.032, 0.176]	PostAge	z =–40.64, $p < 0.001$ [–0.712, –0.627]		

Table S15: Regression results for two-period ATT estimation with the replacement of treatment group. Post-specific random effects are included. Reported are coefficient estimates with standard errors in parentheses. * $p < 0.01$, ** $p < 0.005$, *** $p < 0.001$. Exact z statistics, p values and 99% CIs are reported in Table S16.

	(1) Main	(2) Extended
Display	−0.060	−0.059
	(0.026)	(0.024)
After	−0.084***	−0.084***
	(0.004)	(0.004)
Display×After	0.002	0.002
	(0.004)	(0.004)
PostAge	−0.616***	−0.615***
	(0.002)	(0.002)
Verified		1.428***
		(0.029)
AccountAge		−0.062***
		(0.013)
Followers		0.375***
		(0.017)
Followees		0.075***
		(0.012)
Words		0.111***
		(0.013)
Media		0.875***
		(0.028)
Economy		−0.451***
		(0.038)
Health		0.318***
		(0.042)
Politics		0.257***
		(0.030)
Science		−0.753***
		(0.037)
Positive		−0.131***
		(0.015)
Negative		0.263***
		(0.016)
MFRO		−0.079***
		(0.012)
Intercept	1.409***	−0.194***
	(0.018)	(0.036)
Post-level RE	✓	✓
#Observations	652,976	652,976
#Posts	40,811	40,811

Table S16: Details of z statistics, p values and 99% CIs (in brackets) for the coefficient estimates reported in Table S15.

	(1)<br>Main	(2)<br>Extended
Display	z =–2.32, p =0.020<br>[–0.127, 0.007]	z =–2.42, p =0.016<br>[–0.122, 0.004]
After	z =–20.75, $p < 0.001$<br>[–0.095, –0.074]	z =–20.73, $p < 0.001$<br>[–0.095, –0.074]
Display×After	z =0.38, p =0.708<br>[–0.010, 0.013]	z =0.37, p =0.713<br>[–0.010, 0.013]
PostAge	z =–251.16, $p < 0.001$<br>[–0.622, –0.609]	z =–251.44, $p < 0.001$<br>[–0.622, –0.609]
Verified		z =49.32, $p < 0.001$<br>[1.354, 1.503]
AccountAge		z =–4.85, $p < 0.001$<br>[–0.094, –0.029]
Followers		z =21.96, $p < 0.001$<br>[0.331, 0.419]
Followees		z =6.50, $p < 0.001$<br>[0.045, 0.104]
Words		z =8.31, $p < 0.001$<br>[0.076, 0.145]
Media		z =31.41, $p < 0.001$<br>[0.803, 0.947]
Economy		z =–12.01, $p < 0.001$<br>[–0.548, –0.354]
Health		z =7.57, $p < 0.001$<br>[0.210, 0.426]
Politics		z =8.55, $p < 0.001$<br>[0.180, 0.335]
Science		z =–20.53, $p < 0.001$<br>[–0.848, –0.659]
Positive		z =–8.86, $p < 0.001$<br>[–0.169, –0.093]
Negative		z =16.89, $p < 0.001$<br>[0.223, 0.303]
MFRO		z =–6.55, $p < 0.001$<br>[–0.109, –0.048]
Intercept	z =76.44, $p < 0.001$<br>[1.361, 1.456]	z =–5.44, $p < 0.001$<br>[–0.286, –0.102]
Post-level RE	✓	✓
#Observations	652,976	652,976
#Posts	40,811	40,811

Supplementary Note 7: Sensitivity Analyses

We conducted additional sensitivity analyses on how the efficacy of community notes varied (i) depending on the post age at note display, (ii) over time since the roll-out of the "Community Notes" program in October 2022, (iii) depending on the the number of ratings, the stability and helpfulness scores of community notes, and (iv) across user and post characteristics.

7.1 Sensitivity Across Response Time

We analyzed how the efficacy of community notes varies depending on the post age at note display, i.e., the response time. We conducted the regressions across the source posts grouped by the response time with a window of 4 hours. For instance, the source posts that received displayed notes at 4 and 8 hours from post creation are grouped together for the subset analysis. The regression results are reported in Table S17. We found that the treatment effects were significantly more pronounced for early vs. late community notes. For instance, for community notes displayed within 4 to 8 hours since the creation of the misleading post, the ATT was −0.665 (99% CI: [−0.671, −0.658]; $z = -139.13$, $p < 0.001$). In contrast, the ATT decreased to −0.457 (99% CI: [−0.473, −0.439]; $z = -50.65$, $p < 0.001$) if the community notes was displayed within 20 to 24 hours after post creation. Overall, the efficacy of community notes in reducing the spread of misleading posts was larger if they were displayed earlier.

Table S17: Regression results for two-period ATT estimations over response time, i.e., post age at note display from 4 to 24 hours. Post-specific random effects are included. Reported are coefficient estimates with standard errors in parentheses. * $p < 0.01$, ** $p < 0.005$, *** $p < 0.001$. Exact z statistics, p values and 99% CIs are reported in Table S18.

Response time	4 – 8	8 – 12	12 – 16	16 – 20	20 – 24
Display	1.182***	1.144***	1.134***	1.292***	1.300***
	(0.040)	(0.049)	(0.058)	(0.061)	(0.065)
After	–0.142***	0.052***	0.327***	0.681***	0.114***
	(0.007)	(0.008)	(0.010)	(0.010)	(0.011)
Display × After	–1.092***	–0.984***	–0.931***	–0.815***	–0.610***
	(0.008)	(0.009)	(0.010)	(0.011)	(0.012)
PostAge	–0.656***	–0.485***	–0.749***	–1.407***	–1.385***
	(0.004)	(0.005)	(0.006)	(0.007)	(0.007)
Intercept	1.487***	1.396***	1.279***	1.369***	1.936***
	(0.029)	(0.035)	(0.041)	(0.043)	(0.045)
Post-level RE	✓	✓	✓	✓	✓
#Observations	210,112	145,408	108,832	98,464	91,584

Table S18: Details of z statistics, p values and 99% CIs (in brackets) for the coefficient estimates reported in Table S17.

Response time	4 – 8	8 – 12	12 – 16	16 – 20	20 – 24
Display	z =29.87, p < 0.001	z =23.12, p < 0.001	z =19.49, p < 0.001	z =21.12, p < 0.001	z =20.15, p < 0.001
	[1.080, 1.284]	[1.016, 1.271]	[0.984, 1.284]	[1.135, 1.450]	[1.134, 1.467]
After	z =–19.26, p < 0.001	z =6.30, p < 0.001	z =34.11, p < 0.001	z =65.31, p < 0.001	z =10.16, p < 0.001
	[–0.161, –0.123]	[0.031, 0.073]	[0.303, 0.352]	[0.654, 0.708]	[0.085, 0.143]
Display×After	z =–139.13, p < 0.001	z =–113.22, p < 0.001	z =–92.69, p < 0.001	z =–74.34, p < 0.001	z =–50.65, p < 0.001
	[–1.113, –1.072]	[–1.006, –0.962]	[–0.957, –0.905]	[–0.843, –0.786]	[–0.641, –0.579]
PostAge	z =–154.53, p < 0.001	z =–102.47, p < 0.001	z =–129.49, p < 0.001	z =–213.14, p < 0.001	z =–197.55, p < 0.001
	[–0.667, –0.645]	[–0.498, –0.473]	[–0.764, –0.735]	[–1.424, –1.390]	[–1.403, –1.367]
Intercept	z =51.54, p < 0.001	z =39.57, p < 0.001	z =31.11, p < 0.001	z =31.81, p < 0.001	z =42.61, p < 0.001
	[1.412, 1.561]	[1.305, 1.486]	[1.173, 1.385]	[1.258, 1.480]	[1.819, 2.053]
Post-level RE	✓	✓	✓	✓	✓
#Observations	210,112	145,408	108,832	98,464	91,584

7.2 Sensitivity Across Months From Roll-Out

Next, we examined the changes in the efficacy of community notes since the roll-out of the feature in October 2022. The regression results for the two-period ATT estimation model across months from the roll-out (MFRO) of "Community Notes" program are reported in Table S19. We found that the ATTs in the first month and second month of the community notes program were −0.403 (99% CI: [−0.477, −0.318]; $z = -10.02$, $p < 0.001$) and −0.322 (99% CI: [−0.392, −0.245]; $z = -9.26$, $p < 0.001$), respectively. However, after 21 months, the ATT increased to −0.697 (99% CI: [−0.716, −0.676]; $z = -47.41$, $p < 0.001$). Overall, this suggests an trend of increasing efficacy of community notes in the months following its launch. This may be partly attributed to improvements within the Community Notes program, such as faster note generation (see Supplementary Note 12).

Table S19: Regression results for two-period ATT estimations across months from the roll-out of "Community Notes" program. Post-specific random effects are included. Reported are coefficient estimates with standard errors in parentheses. $^{*}\ p < 0.01$, $^{**}\ p < 0.005$, $^{***}\ p < 0.001$. Exact z statistics, p values and 99% CIs are reported in Table S20.

MFRO	1	2	3	4	5	6	7
Display	1.014**	1.812***	0.856**	1.663***	1.379***	0.937***	1.434***
	(0.358)	(0.273)	(0.273)	(0.266)	(0.216)	(0.169)	(0.146)
After	0.098	−0.009	0.106*	0.205***	0.192***	0.149***	0.058
	(0.046)	(0.038)	(0.041)	(0.038)	(0.033)	(0.026)	(0.024)
Display × After	−0.516***	−0.389***	−0.645***	−0.748***	−0.798***	−0.737***	−0.627***
	(0.051)	(0.042)	(0.042)	(0.042)	(0.036)	(0.029)	(0.026)
PostAge	−0.726***	−0.745***	−0.779***	−0.687***	−0.798***	−0.824***	−0.835***
	(0.028)	(0.023)	(0.023)	(0.022)	(0.019)	(0.016)	(0.014)
Intercept	2.114***	1.775***	2.022***	1.585***	1.285***	1.542***	1.247***
	(0.230)	(0.181)	(0.204)	(0.171)	(0.141)	(0.109)	(0.096)
Post-level RE	✓	✓	✓	✓	✓	✓	✓
#Observations	3,568	5,616	5,584	6,080	9,136	12,992	16,304

MFRO	8	9	10	11	12	13	14
Display	1.296***	1.198***	1.667***	1.471***	1.407***	1.233***	1.017***
	(0.124)	(0.123)	(0.122)	(0.098)	(0.108)	(0.084)	(0.089)
After	0.145***	0.156***	0.244***	0.371***	0.249***	0.084***	0.100***
	(0.021)	(0.020)	(0.021)	(0.017)	(0.019)	(0.014)	(0.015)
Display × After	−0.663***	−0.640***	−0.631***	−0.800***	−0.714***	−0.967***	−0.957***
	(0.023)	(0.022)	(0.023)	(0.018)	(0.020)	(0.015)	(0.016)
PostAge	−0.893***	−0.886***	−0.918***	−0.927***	−0.905***	−0.853***	−0.837***
	(0.012)	(0.012)	(0.012)	(0.010)	(0.011)	(0.008)	(0.009)
Intercept	1.111***	1.349***	1.236***	1.128***	1.194***	1.411***	1.427***
	(0.086)	(0.086)	(0.084)	(0.070)	(0.074)	(0.060)	(0.062)
Post-level RE	✓	✓	✓	✓	✓	✓	✓
#Observations	21,936	22,448	23,856	37,472	27,552	51,616	47,312

MFRO	15	16	17	18	19	20	21
Display	0.990***	1.222***	1.147***	1.240***	1.203***	1.197***	0.974***
	(0.080)	(0.081)	(0.091)	(0.074)	(0.072)	(0.075)	(0.122)
After	0.072***	0.007	0.066***	0.069***	0.056***	0.047**	0.021
	(0.014)	(0.014)	(0.016)	(0.015)	(0.015)	(0.016)	(0.023)
Display × After	−1.014***	−0.989***	−1.089***	−1.070***	−1.066***	−1.188***	−1.192***
	(0.015)	(0.016)	(0.017)	(0.015)	(0.016)	(0.016)	(0.025)
PostAge	−0.707***	−0.732***	−0.742***	−0.749***	−0.777***	−0.723***	−0.701***
	(0.008)	(0.009)	(0.009)	(0.009)	(0.009)	(0.009)	(0.014)
Intercept	1.325***	1.316***	1.440***	1.083***	1.144***	1.158***	1.507***
	(0.058)	(0.056)	(0.064)	(0.056)	(0.054)	(0.055)	(0.086)
Post-level RE	✓	✓	✓	✓	✓	✓	✓
#Observations	56,992	58,624	46,544	60,016	61,136	57,056	22,560

Table S20: Details of z statistics, p values and 99% CIs (in brackets) for the coefficient estimates reported in Table S19.

MFRO	1	2	3	4	5	6	7
Display	z =2.83, p =0.005<br>[0.092, 1.937]	z =6.65, p < 0.001<br>[1.110, 2.515]	z =3.14, p =0.002<br>[0.153, 1.559]	z =6.26, p < 0.001<br>[0.978, 2.347]	z =6.39, p < 0.001<br>[0.823, 1.934]	z =5.55, p < 0.001<br>[0.502, 1.371]	z =9.83, p < 0.001<br>[1.058, 1.809]
After	z =2.13, p =0.033<br>[−0.020, 0.215]	z =−0.24, p =0.808<br>[−0.108, 0.090]	z =2.63, p =0.009<br>[0.002, 0.211]	z =5.42, p < 0.001<br>[0.108, 0.303]	z =5.85, p < 0.001<br>[0.107, 0.276]	z =5.78, p < 0.001<br>[0.083, 0.216]	z =2.45, p =0.014<br>[−0.003, 0.119]
Display×After	z =−10.02, p < 0.001<br>[−0.648, −0.383]	z =−9.26, p < 0.001<br>[−0.497, −0.281]	z =−15.29, p < 0.001<br>[−0.754, −0.537]	z =−17.93, p < 0.001<br>[−0.855, −0.640]	z =−22.31, p < 0.001<br>[−0.890, −0.706]	z =−25.42, p < 0.001<br>[−0.811, −0.662]	z =−24.28, p < 0.001<br>[−0.693, −0.560]
PostAge	z =−26.07, p < 0.001<br>[−0.798, −0.654]	z =−32.91, p < 0.001<br>[−0.803, −0.687]	z =−34.06, p < 0.001<br>[−0.838, −0.720]	z =−30.58, p < 0.001<br>[−0.745, −0.629]	z =−40.93, p < 0.001<br>[−0.848, −0.748]	z =−52.09, p < 0.001<br>[−0.865, −0.783]	z =−58.91, p < 0.001<br>[−0.871, −0.798]
Intercept	z =9.20, p < 0.001<br>[1.522, 2.706]	z =9.83, p < 0.001<br>[1.310, 2.240]	z =9.89, p < 0.001<br>[1.495, 2.549]	z =9.29, p < 0.001<br>[1.145, 2.024]	z =9.10, p < 0.001<br>[0.921, 1.648]	z =14.10, p < 0.001<br>[1.261, 1.824]	z =12.94, p < 0.001<br>[0.998, 1.495]
Post-level RE	✓	✓	✓	✓	✓	✓	✓
#Observations	3,568	5,616	5,584	6,080	9,136	12,992	16,304

MFRO	8	9	10	11	12	13	14
Display	z =10.49, p < 0.001<br>[0.978, 1.615]	z =9.73, p < 0.001<br>[0.881, 1.515]	z =13.67, p < 0.001<br>[1.353, 1.981]	z =15.05, p < 0.001<br>[1.220, 1.723]	z =13.06, p < 0.001<br>[1.130, 1.685]	z =14.61, p < 0.001<br>[1.016, 1.451]	z =11.49, p < 0.001<br>[0.789, 1.245]
After	z =6.79, p < 0.001<br>[0.090, 0.200]	z =7.64, p < 0.001<br>[0.104, 0.209]	z =11.63, p < 0.001<br>[0.190, 0.299]	z =21.34, p < 0.001<br>[0.326, 0.415]	z =13.20, p < 0.001<br>[0.200, 0.298]	z =6.02, p < 0.001<br>[0.048, 0.121]	z =6.79, p < 0.001<br>[0.062, 0.139]
Display×After	z =−29.44, p < 0.001<br>[−0.721, −0.605]	z =−29.27, p < 0.001<br>[−0.696, −0.583]	z =−27.98, p < 0.001<br>[−0.690, −0.573]	z =−43.77, p < 0.001<br>[−0.848, −0.753]	z =−35.39, p < 0.001<br>[−0.766, −0.662]	z =−65.14, p < 0.001<br>[−1.005, −0.928]	z =−60.20, p < 0.001<br>[−0.998, −0.916]
PostAge	z =−71.56, p < 0.001<br>[−0.925, −0.861]	z =−73.53, p < 0.001<br>[−0.917, −0.855]	z =−74.77, p < 0.001<br>[−0.950, −0.887]	z =−93.57, p < 0.001<br>[−0.952, −0.901]	z =−81.80, p < 0.001<br>[−0.934, −0.877]	z =−103.80, p < 0.001<br>[−0.874, −0.832]	z =−95.49, p < 0.001<br>[−0.860, −0.815]
Intercept	z =12.87, p < 0.001<br>[0.889, 1.333]	z =15.72, p < 0.001<br>[1.128, 1.570]	z =14.74, p < 0.001<br>[1.020, 1.453]	z =16.16, p < 0.001<br>[0.948, 1.308]	z =16.09, p < 0.001<br>[1.003, 1.385]	z =23.49, p < 0.001<br>[1.256, 1.566]	z =22.93, p < 0.001<br>[1.267, 1.587]
Post-level RE	✓	✓	✓	✓	✓	✓	✓
#Observations	21,936	22,448	23,856	37,472	27,552	51,616	47,312

MFRO	15	16	17	18	19	20	21
Display	z =12.31, p < 0.001<br>[0.783, 1.198]	z =15.09, p < 0.001<br>[1.014, 1.431]	z =12.62, p < 0.001<br>[0.913, 1.381]	z =16.69, p < 0.001<br>[1.048, 1.431]	z =16.71, p < 0.001<br>[1.018, 1.389]	z =15.97, p < 0.001<br>[1.004, 1.390]	z =7.99, p < 0.001<br>[0.660, 1.288]
After	z =5.12, p < 0.001<br>[0.036, 0.109]	z =0.50, p =0.616<br>[−0.030, 0.044]	z =4.20, p < 0.001<br>[0.026, 0.107]	z =4.60, p < 0.001<br>[0.030, 0.107]	z =3.69, p < 0.001<br>[0.017, 0.095]	z =2.99, p =0.003<br>[0.007, 0.087]	z =0.92, p =0.359<br>[−0.039, 0.082]
Display×After	z =−67.17, p < 0.001<br>[−1.053, −0.975]	z =−62.84, p < 0.001<br>[−1.029, −0.948]	z =−63.83, p < 0.001<br>[−1.132, −1.045]	z =−69.30, p < 0.001<br>[−1.110, −1.030]	z =−67.68, p < 0.001<br>[−1.107, −1.025]	z =−72.35, p < 0.001<br>[−1.230, −1.146]	z =−47.41, p < 0.001<br>[−1.257, −1.128]
PostAge	z =−84.65, p < 0.001<br>[−0.729, −0.685]	z =−84.56, p < 0.001<br>[−0.755, −0.710]	z =−78.71, p < 0.001<br>[−0.767, −0.718]	z =−88.03, p < 0.001<br>[−0.771, −0.727]	z =−90.05, p < 0.001<br>[−0.799, −0.754]	z =−80.36, p < 0.001<br>[−0.747, −0.700]	z =−50.51, p < 0.001<br>[−0.737, −0.665]
Intercept	z =23.00, p < 0.001<br>[1.176, 1.473]	z =23.64, p < 0.001<br>[1.173, 1.459]	z =22.65, p < 0.001<br>[1.276, 1.603]	z =19.50, p < 0.001<br>[0.940, 1.226]	z =21.12, p < 0.001<br>[1.004, 1.283]	z =20.99, p < 0.001<br>[1.016, 1.300]	z =17.56, p < 0.001<br>[1.286, 1.728]
Post-level RE	✓	✓	✓	✓	✓	✓	✓
#Observations	56,992	58,624	46,544	60,016	61,136	57,056	22,560

7.3 Sensitivity Across Rating Thresholds and Note Helpfulness Scores

We analyzed whether the efficacy of community notes varies depending on the number of ratings, i.e., the popularity of notes as reflected by other fact-checking contributors. A higher number of ratings points towards notes that attract a lot of attention from other fact-checking contributors (e.g., because the fact-checked post is particularly viral). Additionally, the Community Notes system sends "Need Your Help" alerts to contributors, requesting ratings for notes with "Needs More Ratings" status. This mechanism promotes engagement with selected notes. Notably, while community notes must receive a minimum number of ratings (e.g., 5) to be assigned a status, highly rated notes are not necessarily displayed, as the algorithm does not rely on majority votes to prevent manipulation. During estimation, we only considered source posts with community notes that indicate potentially misleading information and had never been rated as not helpful, as those notes were more likely to be promoted by "Needs Your Help" alerts.

Figure S8 shows the ATT estimates across the rating thresholds from 10 to 80 (mean of ratings per note) with a step size of 10. We found that the ATT estimates across the rating thresholds had no significant differences with each other. This suggests that once the number of helpfulness ratings exceeded the display threshold, the efficacy of community notes in reducing the spread of misleading posts was relatively stable.

Additionally, we examined whether the stability of the helpful status affect the efficacy of community notes. Based on the original status history dataset, we categorize helpful notes into stable helpful notes (notes that retained a helpful status since their first NonNMR status) and non-stable helpful note (notes whose helpful status later disappeared). The full estimation results are reported in Table S23. We found that the stable helpful notes (ATT: –0.654; 99% CI: [–0.658, –0.650]; $z = -222.56$, $p < 0.001$) had a larger efficacy than non-stable helpful notes (ATT: –0.436; 99% CI: [–0.445, –0.426]; $z = -85.09$, $p < 0.001$).

Furthermore, we analyzed how the efficacy of community notes varied across their helpfulness scores. To this end, we ran the note ranking algorithm using datasets downloaded from the

Community Notes website and calculated note helpfulness scores. The full estimation results are reported in Table S25. We found that the efficacy of community notes increased with the increase of helpfulness scores.

Taken together, community notes reduced the spread of misleading posts across subsets separated by the number of ratings, note stability, and note helpfulness scores. Moreover, their efficacy was strengthened when having a stable helpful status and high helpfulness scores.

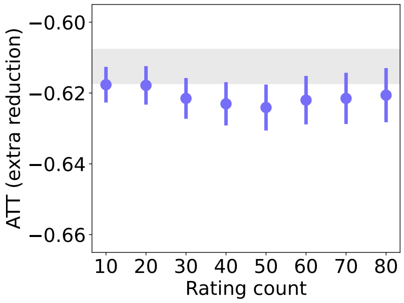

Figure S8: **The estimated ATTs of community notes depending on the number of ratings.** The error bars represent 99% CIs, and the grey bands visualize the ATT (with 99% CI) estimated via the two-period DiD model from our main analysis. The full estimation results are reported in Table S21.

Table S21: Regression results for two-period ATT estimations across rating thresholds. Post-specific random effects are included. Reported are coefficient estimates with standard errors in parentheses. $^{*}\ p < 0.01$, $^{**}\ p < 0.005$, $^{***}\ p < 0.001$. Exact z statistics, p values and 99% CIs are reported in Table S22.

Ratings	10	20	30	40	50	60	70	80
Display	0.151***	−0.233***	−0.414***	−0.503***	−0.545***	−0.558***	−0.564***	−0.566***
	(0.025)	(0.027)	(0.029)	(0.031)	(0.033)	(0.034)	(0.036)	(0.038)
After	0.151***	0.167***	0.183***	0.191***	0.199***	0.199***	0.205***	0.207***
	(0.005)	(0.005)	(0.006)	(0.006)	(0.007)	(0.007)	(0.007)	(0.008)
Display $\times$ After	−0.961***	−0.962***	−0.972***	−0.976***	−0.978***	−0.973***	−0.972***	−0.969***
	(0.005)	(0.006)	(0.006)	(0.006)	(0.007)	(0.007)	(0.007)	(0.008)
PostAge	−0.826***	−0.842***	−0.849***	−0.851***	−0.851***	−0.851***	−0.848***	−0.841***
	(0.003)	(0.003)	(0.003)	(0.003)	(0.003)	(0.003)	(0.003)	(0.003)
Intercept	2.328***	2.731***	2.970***	3.138***	3.266***	3.357***	3.448***	3.524***
	(0.021)	(0.023)	(0.025)	(0.027)	(0.029)	(0.031)	(0.032)	(0.034)
Post-level RE	✓	✓	✓	✓	✓	✓	✓	✓
#Observations	495,664	448,816	413,888	382,736	351,584	324,768	298,624	272,864

Table S22: Details of z statistics, p values and 99% CIs (in brackets) for the coefficient estimates reported in Table S21.

Ratings	10	20	30	40	50	60	70	80
Display	z =5.95, $p < 0.001$	z =–8.53, $p < 0.001$	z =–14.18, $p < 0.001$	z =–16.30, $p < 0.001$	z =–16.72, $p < 0.001$	z =–16.34, $p < 0.001$	z =–15.79, $p < 0.001$	z =–15.06, $p < 0.001$
	[0.086, 0.216]	[–0.304, –0.163]	[–0.489, –0.339]	[–0.582, –0.423]	[–0.629, –0.461]	[–0.645, –0.470]	[–0.656, –0.472]	[–0.662, –0.469]
After	z =30.06, $p < 0.001$	z =30.37, $p < 0.001$	z =30.90, $p < 0.001$	z =30.23, $p < 0.001$	z =29.45, $p < 0.001$	z =28.05, $p < 0.001$	z =27.33, $p < 0.001$	z =26.23, $p < 0.001$
	[0.138, 0.164]	[0.153, 0.181]	[0.168, 0.198]	[0.175, 0.208]	[0.181, 0.216]	[0.181, 0.217]	[0.185, 0.224]	[0.187, 0.228]
Display×After	z =–188.33, $p < 0.001$	z =–174.43, $p < 0.001$	z =–164.57, $p < 0.001$	z =–155.26, $p < 0.001$	z =–146.42, $p < 0.001$	z =–138.61, $p < 0.001$	z =–131.12, $p < 0.001$	z =–124.07, $p < 0.001$
	[–0.975, –0.948]	[–0.976, –0.948]	[–0.987, –0.956]	[–0.992, –0.959]	[–0.996, –0.961]	[–0.991, –0.955]	[–0.991, –0.952]	[–0.989, –0.949]
PostAge	z =–308.20, $p < 0.001$	z =–302.47, $p < 0.001$	z =–295.47, $p < 0.001$	z =–287.56, $p < 0.001$	z =–278.26, $p < 0.001$	z =–270.43, $p < 0.001$	z =–260.50, $p < 0.001$	z =–249.03, $p < 0.001$
	[–0.833, –0.819]	[–0.849, –0.835]	[–0.856, –0.842]	[–0.859, –0.844]	[–0.859, –0.843]	[–0.859, –0.843]	[–0.856, –0.839]	[–0.849, –0.832]
Intercept	z =112.37, $p < 0.001$	z =117.18, $p < 0.001$	z =116.57, $p < 0.001$	z =114.85, $p < 0.001$	z =111.98, $p < 0.001$	z =109.30, $p < 0.001$	z =106.74, $p < 0.001$	z =103.38, $p < 0.001$
	[2.274, 2.381]	[2.671, 2.792]	[2.904, 3.036]	[3.067, 3.208]	[3.191, 3.341]	[3.278, 3.436]	[3.365, 3.532]	[3.436, 3.612]
Post-level RE	✓	✓	✓	✓	✓	✓	✓	✓
#Observations	495,664	448,816	413,888	382,736	351,584	324,768	298,624	272,864

Table S23: Regression results for two-period ATT estimations depending on the stability of note helpful status. Post-specific random effects are included. Reported are coefficient estimates with standard errors in parentheses. * $p < 0.01$, ** $p < 0.005$, *** $p < 0.001$. Exact z statistics, p values and 99% CIs are reported in Table S24.

	Non-stable helpful	Stable helpful
Display	1.317***	1.161***
	(0.041)	(0.026)
After	–0.070***	0.132***
	(0.005)	(0.004)
Display × After	–0.572***	–1.061***
	(0.007)	(0.005)
PostAge	–0.630***	–0.822***
	(0.003)	(0.003)
Intercept	1.412***	1.267***
	(0.018)	(0.017)
Post-level RE	✓	✓
#Observations	403,168	578,432

Table S24: Details of z statistics, p values and 99% CIs (in brackets) for the coefficient estimates reported in Table S23.

	Non-stable helpful	Stable helpful
Display	z =32.22, p < 0.001	z =45.42, p < 0.001
	[1.212, 1.422]	[1.095, 1.227]
After	z =−15.41, p < 0.001	z =30.76, p < 0.001
	[−0.081, −0.058]	[0.121, 0.144]
Display×After	z =−85.09, p < 0.001	z =−222.56, p < 0.001
	[−0.589, −0.555]	[−1.073, −1.049]
PostAge	z =−205.10, p < 0.001	z =−312.58, p < 0.001
	[−0.637, −0.622]	[−0.828, −0.815]
Intercept	z =79.62, p < 0.001	z =74.91, p < 0.001
	[1.366, 1.458]	[1.223, 1.311]
Post-level RE	✓	✓
#Observations	403,168	578,432

Table S25: Regression results for two-period ATT estimations across note helpfulness scores. Post-specific random effects are included. Reported are coefficient estimates with standard errors in parentheses. * $p < 0.01$, ** $p < 0.005$, *** $p < 0.001$. Exact z statistics, p values and 99% CIs are reported in Table S26.

Note scores	0.4 – 0.45	0.45 – 0.50	0.50 – 0.55	0.55 – 0.60
Display	0.228***	0.380***	0.674***	1.184***
	(0.044)	(0.040)	(0.043)	(0.070)
After	−0.010	0.016**	0.023***	−0.035***
	(0.005)	(0.005)	(0.005)	(0.005)
Display × After	−0.992***	−1.110***	−1.217***	−1.109***
	(0.008)	(0.008)	(0.008)	(0.012)
PostAge	−0.678***	−0.703***	−0.709***	−0.654***
	(0.003)	(0.003)	(0.003)	(0.004)
Intercept	2.134***	2.114***	2.110***	2.152***
	(0.019)	(0.019)	(0.019)	(0.019)
Post-level RE	✓	✓	✓	✓
#Observations	287,488	298,384	288,880	252,144

Table S26: Details of z statistics, p values and 99% CIs (in brackets) for the coefficient estimates reported in Table S25.

Note scores	0.4 – 0.45	0.45 – 0.50	0.50 – 0.55	0.55 – 0.60
Display	z =5.24, $p < 0.001$	z =9.43, $p < 0.001$	z =15.69, $p < 0.001$	z =16.93, $p < 0.001$
	[0.116, 0.340]	[0.276, 0.484]	[0.563, 0.785]	[1.004, 1.364]
After	z =−1.96, p =0.050	z =3.28, p =0.001	z =4.46, $p < 0.001$	z =−6.75, $p < 0.001$
	[−0.023, 0.003]	[0.004, 0.029]	[0.010, 0.036]	[−0.048, −0.021]
Display×After	z =−122.33, $p < 0.001$	z =−147.95, $p < 0.001$	z =−154.25, $p < 0.001$	z =−92.76, $p < 0.001$
	[−1.013, −0.971]	[−1.130, −1.091]	[−1.238, −1.197]	[−1.140, −1.078]
PostAge	z =−197.84, $p < 0.001$	z =−207.87, $p < 0.001$	z =−206.94, $p < 0.001$	z =−182.46, $p < 0.001$
	[−0.686, −0.669]	[−0.711, −0.694]	[−0.718, −0.700]	[−0.663, −0.645]
Intercept	z =111.88, $p < 0.001$	z =111.35, $p < 0.001$	z =111.06, $p < 0.001$	z =111.88, $p < 0.001$
	[2.085, 2.183]	[2.065, 2.163]	[2.061, 2.159]	[2.103, 2.202]
Post-level RE	✓	✓	✓	✓
#Observations	287,488	298,384	288,880	252,144

7.4 Sensitivity Across Poster and Post Characteristics

We studied whether the treatment effect of community notes was moderated by author characteristics (e.g., the number of followers, verified status) and post characteristics (e.g., sentiment, topics). Table S27 reports the regression results for the sensitivity analysis on how the efficacy of community notes varied depending on poster characteristics ($Verified$, $AccountAge$, $Followers$, and $Followees$). Table S29 and Table S31 report the regression results for the sensitivity analysis on how the efficacy of community notes varied depending on post characteristics ($Words$, $Media$, $Positive$, $Negative$, $Economy$, $Health$, $Politics$, and $Science$).

We found that the ATT estimates were significantly negative across all author characteristics. However, the efficacy of community notes was larger if the author of the fact-checked post was not verified (ATT: −0.732; 99% CI: [−0.741, −0.723]; $z = -104.72$, $p < 0.001$), for younger accounts (ATT: −0.651; 99% CI: [−0.657, −0.645]; $z = -164.28$, $p < 0.001$), and for accounts with a lower number of followers (ATT: −0.687; 99% CI: [−0.693, −0.681]; $z = -151.26$, $p < 0.001$), as compared to fact-checked post from verified accounts (ATT: −0.580; 99% CI: [−0.586, −0.575]; $z = -183.75$, $p < 0.001$), older accounts (ATT: −0.570; 99% CI: [−0.577, −0.563]; $z = -135.11$, $p < 0.001$), and accounts with higher number of followers (ATT: −0.549; 99% CI: [−0.555, −0.543]; $z = -147.48$, $p < 0.001$). This suggests that the effectiveness of community notes might be slightly discounted for source posts from accounts with high social influence.

In terms of post characteristics, the ATT estimates were larger for posts that were shorter (ATT: −0.644; 99% CI: [−0.650, −0.638]; $z = -157.45$, $p < 0.001$) and posts that had media elements (ATT: −0.634; 99% CI: [−0.639, −0.629]; $z = -197.12$, $p < 0.001$), as compared to posts that were longer (ATT: −0.579; 99% CI: [−0.585, −0.572]; $z = -141.64$, $p < 0.001$) and posts without media elements (ATT: −0.534; 99% CI: [−0.545, −0.523]; $z = -82.34$, $p < 0.001$). Moreover, we used a state-of-the-art machine learning model to calculate positive and negative sentiment scores for the fact-checked posts (see Supplementary Note 2). Here, we found that the efficacy of community notes was robust and had no significant difference between posts with low negative

sentiment (ATT: –0.615; 99% CI: [–0.622, –0.609]; z = –146.22, $p < 0.001$) and those with high negative sentiment (ATT: –0.610; 99% CI: [–0.616, –0.604]; z = –153.05, $p < 0.001$), and between posts with low positive sentiment (ATT: –0.606; 99% CI: [–0.612, –0.600]; z = –151.97, $p < 0.001$) and those with high positive sentiment (ATT: –0.620; 99% CI: [–0.626, –0.613]; z = –147.49, $p < 0.001$). Ultimately, we implemented (and validated) a topic modeling approach (see Supplementary Note 3) to study how the efficacy of community notes varies across different topics, namely, *Economy*, *Health*, *Politics*, and *Science*. Posts that did not fall into one of these topic categories were categorized as OTHER. Here, we found that the ATT was smaller for posts related to economy (ATT: –0.575; 99% CI: [–0.590, –0.560]; z = –62.59, $p < 0.001$), health (ATT: –0.549; 99% CI: [–0.564, –0.535]; z = –63.25, $p < 0.001$), and politics (ATT: –0.567; 99% CI: [–0.577, –0.558]; z = –98.92, $p < 0.001$), as compared to posts not related to economy (ATT: –0.617; 99% CI: [–0.621, –0.612]; z = –202.51, $p < 0.001$), health (ATT: –0.618; 99% CI: [–0.623, –0.614]; z = –201.74, $p < 0.001$), and politics (ATT: –0.629; 99% CI: [–0.634, –0.624]; z = –187.94, $p < 0.001$). This suggests that users have a higher resistance towards community notes if they are attached to posts covering economy, health, and political topics. We found no such statistically significant difference for science-related posts (ATT: –0.614; 99% CI: [–0.626, –0.601]; z = –74.71, $p < 0.001$), as compared to posts not covering science-related topics (ATT: –0.629; 99% CI: [–0.634, –0.624]; z = –198.51, $p < 0.001$).

Table S27: Regression results for two-period ATT estimations across user characteristics, i.e., *Verified*, *AccountAge*, *Followers* and *Followees*. The estimation models incorporate interaction terms and subsets separately. Post-specific random effects are included. Reported are coefficient estimates with standard errors in parentheses. $^{*}\ p < 0.01$, $^{**}\ p < 0.005$, $^{***}\ p < 0.001$. Exact z statistics, p values and 99% CIs are reported in Table S28.

	Interaction	Verified	Not verified
Display	1.849***	0.974***	1.877***
	(0.049)	(0.025)	(0.054)
After	0.237***	0.085***	0.205***
	(0.008)	(0.004)	(0.012)
Display×After	−1.328***	−0.869***	−1.317***
	(0.011)	(0.005)	(0.013)
PostAge	−0.797***	−0.799***	−0.785***
	(0.002)	(0.003)	(0.007)
Verified	1.650***		
	(0.038)		
Display×Verified	−0.871***		
	(0.055)		
After×Verified	−0.158***		
	(0.009)		
ATT×Verified	0.460***		
	(0.012)		
Intercept	0.028	1.679***	0.006
	(0.033)	(0.018)	(0.037)
Post-level RE	✓	✓	✓
#Observations	654,400	508,640	145,760

	Interaction	High account age	Low account age
Display	1.175***	1.026***	1.377***
	(0.024)	(0.032)	(0.034)
After	0.102***	0.059***	0.155***
	(0.004)	(0.006)	(0.006)
Display×After	−0.933***	−0.843***	−1.053***
	(0.004)	(0.006)	(0.006)
PostAge	−0.797***	−0.798***	−0.797***
	(0.002)	(0.003)	(0.004)
AccountAge	0.088***		
	(0.017)		
Display×AccountAge	−0.225***		
	(0.024)		
After×AccountAge	−0.055***		
	(0.003)		
ATT×AccountAge	0.134***		
	(0.005)		
Intercept	1.301***	1.356***	1.228***
	(0.017)	(0.023)	(0.024)
Post-level RE	✓	✓	✓
#Observations	654,400	327,184	327,216

	Interaction	High followers	Low followers
Display	1.196***	0.559***	1.664***
	(0.023)	(0.029)	(0.033)
After	0.106***	0.072***	0.148***
	(0.004)	(0.005)	(0.007)
Display×After	−0.943***	−0.796***	−1.162***
	(0.004)	(0.005)	(0.008)
PostAge	−0.797***	−0.805***	−0.781***
	(0.002)	(0.003)	(0.004)
Followers	0.365***		
	(0.024)		
Display×Followers	−0.155***		
	(0.036)		
After×Followers	−0.030***		
	(0.004)		
ATT×Followers	0.116***		
	(0.006)		
Intercept	1.310***	2.418***	0.292***
	(0.017)	(0.022)	(0.023)
Post-level RE	✓	✓	✓
#Observations	654,400	327,120	327,280

	Interaction	High followees	Low followees
Display	1.194***	1.161***	1.228***
	(0.023)	(0.032)	(0.034)
After	0.106***	0.116***	0.095***
	(0.004)	(0.006)	(0.006)
Display×After	−0.946***	−0.993***	−0.897***
	(0.004)	(0.006)	(0.006)
PostAge	−0.797***	−0.814***	−0.779***
	(0.002)	(0.003)	(0.003)
Followees	0.130***		
	(0.016)		
Display×Followees	0.076**		
	(0.024)		
After×Followees	0.000		
	(0.003)		
ATT×Followees	−0.034***		
	(0.004)		
Intercept	1.293***	1.444***	1.146***
	(0.017)	(0.023)	(0.024)
Post-level RE	✓	✓	✓
#Observations	654,400	327,152	327,248

Table S28: Details of z statistics, p values and 99% CIs (in brackets) for the coefficient estimates reported in Table S27.

	Interaction	Verified	Not verified
Display	z =38.11, $p < 0.001$ [1.724, 1.974]	z =38.69, $p < 0.001$ [0.909, 1.039]	z =34.95, $p < 0.001$ [1.738, 2.015]
After	z =28.46, $p < 0.001$ [0.216, 0.259]	z =19.04, $p < 0.001$ [0.073, 0.096]	z =17.39, $p < 0.001$ [0.174, 0.235]
Display×After	z =−122.84, $p < 0.001$ [−1.356, −1.300]	z =−183.75, $p < 0.001$ [−0.881, −0.856]	z =−104.72, $p < 0.001$ [−1.350, −1.285]
PostAge	z =−324.58, $p < 0.001$ [−0.803, −0.790]	z =−308.11, $p < 0.001$ [−0.806, −0.793]	z =−112.70, $p < 0.001$ [−0.803, −0.767]
Verified	z =43.77, $p < 0.001$ [1.553, 1.747]		
Display×Verified	z =−15.84, $p < 0.001$ [−1.013, −0.730]		
After×Verified	z =−18.15, $p < 0.001$ [−0.180, −0.135]		
ATT×Verified	z =38.74, $p < 0.001$ [0.429, 0.490]		
Intercept	z =0.85, p =0.394 [−0.057, 0.113]	z =92.80, $p < 0.001$ [1.633, 1.726]	z =0.16, p =0.873 [−0.089, 0.101]
Post-level RE	✓	✓	✓
#Observations	654,400	508,640	145,760

	Interaction	High account age	Low account age
Display	z =49.99, $p < 0.001$ [1.115, 1.236]	z =31.88, $p < 0.001$ [0.943, 1.109]	z =40.63, $p < 0.001$ [1.289, 1.464]
After	z =24.31, $p < 0.001$ [0.091, 0.113]	z =10.13, $p < 0.001$ [0.044, 0.074]	z =25.85, $p < 0.001$ [0.140, 0.171]
Display×After	z =−207.42, $p < 0.001$ [−0.945, −0.921]	z =−135.11, $p < 0.001$ [−0.859, −0.827]	z =−164.28, $p < 0.001$ [−1.070, −1.037]
PostAge	z =−324.43, $p < 0.001$ [−0.804, −0.791]	z =−231.58, $p < 0.001$ [−0.806, −0.789]	z =−227.17, $p < 0.001$ [−0.806, −0.788]
AccountAge	z =5.32, $p < 0.001$ [0.045, 0.131]		
Display×AccountAge	z =−9.45, $p < 0.001$ [−0.286, −0.164]		
After×AccountAge	z =−16.26, $p < 0.001$ [−0.064, −0.046]		
ATT×AccountAge	z =28.93, $p < 0.001$ [0.122, 0.146]		
Intercept	z =77.97, $p < 0.001$ [1.258, 1.344]	z =59.17, $p < 0.001$ [1.297, 1.415]	z =51.16, $p < 0.001$ [1.166, 1.290]
Post-level RE	✓	✓	✓
#Observations	654,400	327,184	327,216

	Interaction	High followers	Low followers
Display	z =51.18, $p < 0.001$ [1.136, 1.257]	z =19.07, $p < 0.001$ [0.484, 0.635]	z =50.42, $p < 0.001$ [1.579, 1.749]
After	z =25.24, $p < 0.001$ [0.095, 0.117]	z =14.19, $p < 0.001$ [0.059, 0.085]	z =20.59, $p < 0.001$ [0.129, 0.166]
Display×After	z =−210.48, $p < 0.001$ [−0.954, −0.931]	z =−147.48, $p < 0.001$ [−0.810, −0.782]	z =−151.26, $p < 0.001$ [−1.181, −1.142]
PostAge	z =−324.24, $p < 0.001$ [−0.804, −0.791]	z =−274.43, $p < 0.001$ [−0.813, −0.797]	z =−182.59, $p < 0.001$ [−0.792, −0.770]
Followers	z =15.53, $p < 0.001$ [0.305, 0.426]		
Display×Followers	z =−4.28, $p < 0.001$ [−0.248, −0.061]		
After×Followers	z =−7.34, $p < 0.001$ [−0.040, −0.019]		
ATT×Followers	z =19.83, $p < 0.001$ [0.101, 0.132]		
Intercept	z =79.03, $p < 0.001$ [1.268, 1.353]	z =112.14, $p < 0.001$ [2.362, 2.473]	z =12.78, $p < 0.001$ [0.233, 0.350]
Post-level RE	✓	✓	✓
#Observations	654,400	327,120	327,280

	Interaction	High followees	Low followees
Display	z =51.19, $p < 0.001$ [1.134, 1.254]	z =36.81, $p < 0.001$ [1.080, 1.242]	z =35.67, $p < 0.001$ [1.139, 1.317]
After	z =25.27, $p < 0.001$ [0.095, 0.117]	z =19.55, $p < 0.001$ [0.101, 0.132]	z =16.05, $p < 0.001$ [0.080, 0.110]
Display×After	z =−211.04, $p < 0.001$ [−0.957, −0.934]	z =−156.96, $p < 0.001$ [−1.009, −0.976]	z =−141.74, $p < 0.001$ [−0.913, −0.880]
PostAge	z =−324.16, $p < 0.001$ [−0.804, −0.791]	z =−234.96, $p < 0.001$ [−0.823, −0.805]	z =−223.52, $p < 0.001$ [−0.788, −0.770]
Followees	z =8.13, $p < 0.001$ [0.089, 0.171]		
Display×Followees	z =3.18, p =0.001 [0.014, 0.138]		
After×Followees	z =−0.09, p =0.931 [−0.008, 0.007]		
ATT×Followees	z =−7.77, $p < 0.001$ [−0.045, −0.023]		
Intercept	z =78.06, $p < 0.001$ [1.251, 1.336]	z =63.57, $p < 0.001$ [1.385, 1.502]	z =47.52, $p < 0.001$ [1.084, 1.208]
Post-level RE	✓	✓	✓
#Observations	654,400	327,152	327,248

Table S29: Regression results for two-period ATT estimations across post characteristics including *Words*, *Media*, *Positive* and *Negative*. The estimation models incorporate interaction terms and subsets separately. Post-specific random effects are included. Reported are coefficient estimates with standard errors in parentheses. * $p < 0.01$, ** $p < 0.005$, *** $p < 0.001$. Exact z statistics, p values and 99% CIs are reported in Table S30.

	Interaction	High words	Low words
Display	1.168***	1.018***	1.389***
	(0.024)	(0.032)	(0.034)
After	0.103***	0.058***	0.159***
	(0.004)	(0.006)	(0.006)
Display×After	−0.933***	−0.864***	−1.034***
	(0.005)	(0.006)	(0.007)
PostAge	−0.797***	−0.779***	−0.816***
	(0.002)	(0.003)	(0.004)
Words	0.205***		
	(0.017)		
Display×Words	−0.206***		
	(0.024)		
After×Words	−0.032***		
	(0.003)		
ATT×Words	0.098***		
	(0.004)		
Intercept	1.325***	1.466***	1.112***
	(0.017)	(0.023)	(0.024)
Post-level RE	✓	✓	✓
#Observations	654,400	325,856	328,544

	Interaction	Media	No media
Display	1.092***	1.213***	1.107***
	(0.044)	(0.027)	(0.047)
After	0.054***	0.131***	0.035***
	(0.007)	(0.005)	(0.009)
Display×After	−0.766***	−1.006***	−0.764***
	(0.009)	(0.005)	(0.009)
PostAge	−0.797***	−0.802***	−0.782***
	(0.002)	(0.003)	(0.005)
Media	0.612***		
	(0.036)		
Display×Media	0.126		
	(0.052)		
After×Media	0.070***		
	(0.008)		
ATT×Media	−0.240***		
	(0.010)		
Intercept	0.855***	1.469***	0.846***
	(0.031)	(0.019)	(0.033)
Post-level RE	✓	✓	✓
#Observations	654,400	475,008	179,392

	Interaction	High positive	Low positive
Display	1.189***	1.422***	0.975***
	(0.023)	(0.033)	(0.033)
After	0.107***	0.121***	0.093***
	(0.004)	(0.006)	(0.006)
Display×After	−0.948***	−0.967***	−0.931***
	(0.004)	(0.007)	(0.006)
PostAge	−0.797***	−0.789***	−0.804***
	(0.002)	(0.004)	(0.003)
Positive	−0.372***		
	(0.016)		
Display×Positive	0.203***		
	(0.023)		
After×Positive	0.012***		
	(0.003)		
ATT×Positive	−0.001		
	(0.005)		
Intercept	1.304***	0.960***	1.630***
	(0.017)	(0.024)	(0.023)
Post-level RE	✓	✓	✓
#Observations	654,400	327,200	327,200

	Interaction	High negative	Low negative
Display	1.184***	0.975***	1.425***
	(0.023)	(0.032)	(0.033)
After	0.106***	0.095***	0.118***
	(0.004)	(0.006)	(0.006)
Display×After	−0.948***	−0.941***	−0.955***
	(0.004)	(0.006)	(0.007)
PostAge	−0.797***	−0.794***	−0.800***
	(0.002)	(0.003)	(0.004)
Negative	0.381***		
	(0.016)		
Display×Negative	−0.239***		
	(0.023)		
After×Negative	−0.008		
	(0.003)		
ATT×Negative	0.006		
	(0.005)		
Intercept	1.321***	1.649***	0.937***
	(0.017)	(0.023)	(0.024)
Post-level RE	✓	✓	✓
#Observations	654,400	327,200	327,200

Table S30: Details of z statistics, p values and 99% CIs (in brackets) for the coefficient estimates reported in Table S29.

	Interaction	High words	Low words
Display	z =49.26, p < 0.001 [1.107, 1.229]	z =31.76, p < 0.001 [0.936, 1.101]	z =40.88, p < 0.001 [1.301, 1.476]
After	z =24.43, p < 0.001 [0.092, 0.114]	z =10.13, p < 0.001 [0.043, 0.072]	z =25.73, p < 0.001 [0.143, 0.175]
Display×After	z =−206.44, p < 0.001 [−0.944, −0.921]	z =−141.64, p < 0.001 [−0.880, −0.849]	z =−157.45, p < 0.001 [−1.051, −1.017]
PostAge	z =−324.26, p < 0.001 [−0.804, −0.791]	z =−231.20, p < 0.001 [−0.788, −0.770]	z =−227.49, p < 0.001 [−0.825, −0.807]
Words	z =12.33, p < 0.001 [0.162, 0.247]		
Display×Words	z =−8.68, p < 0.001 [−0.267, −0.145]		
After×Words	z =−9.98, p < 0.001 [−0.041, −0.024]		
ATT×Words	z =21.84, p < 0.001 [0.086, 0.109]		
Intercept	z =78.80, p < 0.001 [1.282, 1.368]	z =64.75, p < 0.001 [1.408, 1.524]	z =45.82, p < 0.001 [1.050, 1.175]
Post-level RE	✓	✓	✓
#Observations	654,400	325,856	328,544

	Interaction	Media	No media
Display	z =24.65, p < 0.001 [0.978, 1.206]	z =45.67, p < 0.001 [1.145, 1.282]	z =23.43, p < 0.001 [0.985, 1.229]
After	z =7.79, p < 0.001 [0.036, 0.072]	z =27.28, p < 0.001 [0.119, 0.143]	z =4.04, p < 0.001 [0.013, 0.057]
Display×After	z =−85.52, p < 0.001 [−0.789, −0.743]	z =−197.12, p < 0.001 [−1.019, −0.993]	z =−82.34, p < 0.001 [−0.788, −0.741]
PostAge	z =−324.49, p < 0.001 [−0.804, −0.791]	z =−287.16, p < 0.001 [−0.809, −0.795]	z =−151.91, p < 0.001 [−0.796, −0.769]
Media	z =16.90, p < 0.001 [0.519, 0.706]		
Display×Media	z =2.42, p =0.015 [−0.008, 0.260]		
After×Media	z =9.39, p < 0.001 [0.051, 0.090]		
ATT×Media	z =−23.18, p < 0.001 [−0.266, −0.213]		
Intercept	z =27.85, p < 0.001 [0.776, 0.935]	z =77.25, p < 0.001 [1.420, 1.518]	z =25.69, p < 0.001 [0.761, 0.931]
Post-level RE	✓	✓	✓
#Observations	654,400	475,008	179,392

	Interaction	High positive	Low positive
Display	z =51.18, p < 0.001 [1.129, 1.249]	z =42.79, p < 0.001 [1.336, 1.508]	z =29.95, p < 0.001 [0.891, 1.059]
After	z =25.34, p < 0.001 [0.096, 0.117]	z =19.65, p < 0.001 [0.105, 0.137]	z =16.16, p < 0.001 [0.078, 0.107]
Display×After	z =−211.47, p < 0.001 [−0.959, −0.936]	z =−147.49, p < 0.001 [−0.984, −0.950]	z =−151.97, p < 0.001 [−0.947, −0.915]
PostAge	z =−324.05, p < 0.001 [−0.803, −0.791]	z =−218.80, p < 0.001 [−0.798, −0.780]	z =−239.20, p < 0.001 [−0.813, −0.795]
Positive	z =−23.05, p < 0.001 [−0.413, −0.330]		
Display×Positive	z =8.72, p < 0.001 [0.143, 0.264]		
After×Positive	z =3.47, p < 0.001 [0.003, 0.021]		
ATT×Positive	z =−0.15, p =0.879 [−0.013, 0.012]		
Intercept	z =78.95, p < 0.001 [1.261, 1.346]	z =40.84, p < 0.001 [0.900, 1.021]	z =70.15, p < 0.001 [1.570, 1.689]
Post-level RE	✓	✓	✓
#Observations	654,400	327,200	327,200

	Interaction	High negative	Low negative
Display	z =50.85, p < 0.001 [1.124, 1.244]	z =30.04, p < 0.001 [0.892, 1.059]	z =42.83, p < 0.001 [1.340, 1.511]
After	z =25.21, p < 0.001 [0.095, 0.117]	z =16.65, p < 0.001 [0.081, 0.110]	z =19.09, p < 0.001 [0.102, 0.134]
Display×After	z =−211.57, p < 0.001 [−0.959, −0.936]	z =−153.05, p < 0.001 [−0.957, −0.925]	z =−146.22, p < 0.001 [−0.972, −0.939]
PostAge	z =−324.02, p < 0.001 [−0.803, −0.791]	z =−235.73, p < 0.001 [−0.803, −0.785]	z =−222.32, p < 0.001 [−0.809, −0.791]
Negative	z =23.31, p < 0.001 [0.339, 0.423]		
Display×Negative	z =−10.20, p < 0.001 [−0.299, −0.179]		
After×Negative	z =−2.51, p =0.012 [−0.017, 0.000]		
ATT×Negative	z =1.34, p =0.181 [−0.006, 0.018]		
Intercept	z =79.86, p < 0.001 [1.279, 1.364]	z =71.34, p < 0.001 [1.590, 1.709]	z =39.72, p < 0.001 [0.876, 0.998]
Post-level RE	✓	✓	✓
#Observations	654,400	327,200	327,200

Table S31: Regression results for two-period ATT estimations across post characteristics including $Economy$, $Health$, $Politics$ and $Science$. The estimation models incorporate interaction terms and subsets separately. Post-specific random effects are included. Reported are coefficient estimates with standard errors in parentheses. * $p < 0.01$, ** $p < 0.005$, *** $p < 0.001$. Exact z statistics, p values and 99% CIs are reported in Table S32.

	Interaction	With economy	Without economy
Display	1.172***	1.367***	1.171***
	(0.025)	(0.068)	(0.025)
After	0.109***	0.067***	0.111***
	(0.004)	(0.013)	(0.004)
Display×After	−0.959***	−0.856***	−0.959***
	(0.005)	(0.014)	(0.005)
PostAge	−0.797***	−0.782***	−0.799***
	(0.002)	(0.008)	(0.003)
Economy	−0.606***		
	(0.049)		
Display×Economy	0.191*		
	(0.071)		
After×Economy	−0.023		
	(0.010)		
ATT×Economy	0.101***		
	(0.014)		
Intercept	1.371***	0.772***	1.370***
	(0.018)	(0.048)	(0.018)
Post-level RE	✓	✓	✓
#Observations	654,400	80,416	573,984

	Interaction	With health	Without health
Display	1.231***	0.922***	1.233***
	(0.025)	(0.070)	(0.025)
After	0.100***	0.031*	0.115***
	(0.004)	(0.012)	(0.004)
Display×After	−0.963***	−0.797***	−0.963***
	(0.005)	(0.013)	(0.005)
PostAge	−0.798***	−0.672***	−0.813***
	(0.002)	(0.007)	(0.003)
Health	0.514***		
	(0.055)		
Display×Health	−0.299***		
	(0.079)		
After×Health	0.061***		
	(0.010)		
ATT×Health	0.162***		
	(0.014)		
Intercept	1.242***	1.877***	1.228***
	(0.017)	(0.049)	(0.018)
Post-level RE	✓	✓	✓
#Observations	654,400	63,040	591,360

	Interaction	With politics	With politics
Display	1.315***	0.869***	1.320***
	(0.027)	(0.043)	(0.028)
After	0.136***	0.035***	0.135***
	(0.005)	(0.008)	(0.005)
Display×After	−0.991***	−0.838***	−0.991***
	(0.005)	(0.008)	(0.005)
PostAge	−0.797***	−0.800***	−0.796***
	(0.002)	(0.005)	(0.003)
Politics	0.631***		
	(0.038)		
Display×Politics	−0.437***		
	(0.053)		
After×Politics	−0.106***		
	(0.007)		
ATT×Politics	0.153***		
	(0.010)		
Intercept	1.130***	1.771***	1.123***
	(0.019)	(0.031)	(0.020)
Post-level RE	✓	✓	✓
#Observations	654,400	167,040	487,360

	Interaction	With science	Without science
Display	1.122***	1.684***	1.122***
	(0.025)	(0.063)	(0.025)
After	0.093***	0.180***	0.097***
	(0.004)	(0.012)	(0.004)
Display×After	−0.949***	−0.951***	−0.949***
	(0.005)	(0.013)	(0.005)
PostAge	−0.797***	−0.769***	−0.800***
	(0.002)	(0.007)	(0.003)
Science	−1.092***		
	(0.047)		
Display×Science	0.561***		
	(0.068)		
After×Science	0.114***		
	(0.010)		
ATT×Science	−0.003		
	(0.014)		
Intercept	1.443***	0.371***	1.440***
	(0.018)	(0.044)	(0.018)
Post-level RE	✓	✓	✓
#Observations	654,400	89,600	564,800

Table S32: Details of z statistics, p values and 99% CIs (in brackets) for the coefficient estimates reported in Table S31.

	Interaction	With economy	Without economy
Display	$z=47.06$, $p<0.001$<br>[1.108, 1.236]	$z=20.06$, $p<0.001$<br>[1.191, 1.542]	$z=47.20$, $p<0.001$<br>[1.107, 1.235]
After	$z=24.93$, $p<0.001$<br>[0.098, 0.120]	$z=5.21$, $p<0.001$<br>[0.034, 0.100]	$z=25.00$, $p<0.001$<br>[0.100, 0.123]
Display×After	$z=-201.78$, $p<0.001$<br>[−0.971, −0.947]	$z=-62.59$, $p<0.001$<br>[−0.892, −0.821]	$z=-202.51$, $p<0.001$<br>[−0.971, −0.947]
PostAge	$z=-324.11$, $p<0.001$<br>[−0.803, −0.791]	$z=-103.37$, $p<0.001$<br>[−0.801, −0.762]	$z=-307.31$, $p<0.001$<br>[−0.806, −0.792]
Economy	$z=-12.32$, $p<0.001$<br>[−0.733, −0.480]		
Display×Economy	$z=2.69$, $p=0.007$<br>[0.008, 0.374]		
After×Economy	$z=-2.22$, $p=0.026$<br>[−0.050, 0.004]		
ATT×Economy	$z=7.17$, $p<0.001$<br>[0.065, 0.138]		
Intercept	$z=77.39$, $p<0.001$<br>[1.325, 1.417]	$z=16.23$, $p<0.001$<br>[0.650, 0.895]	$z=77.51$, $p<0.001$<br>[1.324, 1.415]
Post-level RE	✓	✓	✓
#Observations	654,400	80,416	573,984

	Interaction	With health	Without health
Display	$z=50.15$, $p<0.001$<br>[1.168, 1.294]	$z=13.22$, $p<0.001$<br>[0.743, 1.102]	$z=49.83$, $p<0.001$<br>[1.169, 1.297]
After	$z=22.91$, $p<0.001$<br>[0.089, 0.111]	$z=2.65$, $p=0.008$<br>[0.001, 0.061]	$z=25.71$, $p<0.001$<br>[0.104, 0.127]
Display×After	$z=-203.34$, $p<0.001$<br>[−0.975, −0.950]	$z=-63.25$, $p<0.001$<br>[−0.830, −0.765]	$z=-201.74$, $p<0.001$<br>[−0.976, −0.951]
PostAge	$z=-324.35$, $p<0.001$<br>[−0.804, −0.791]	$z=-97.54$, $p<0.001$<br>[−0.689, −0.654]	$z=-309.47$, $p<0.001$<br>[−0.820, −0.806]
Health	$z=9.36$, $p<0.001$<br>[0.372, 0.655]		
Display×Health	$z=-3.78$, $p<0.001$<br>[−0.503, −0.095]		
After×Health	$z=5.88$, $p<0.001$<br>[0.034, 0.088]		
ATT×Health	$z=11.18$, $p<0.001$<br>[0.125, 0.199]		
Intercept	$z=71.17$, $p<0.001$<br>[1.197, 1.287]	$z=38.50$, $p<0.001$<br>[1.751, 2.003]	$z=69.73$, $p<0.001$<br>[1.182, 1.273]
Post-level RE	✓	✓	✓
#Observations	654,400	63,040	591,360

	Interaction	With politics	With politics
Display	$z=48.71$, $p<0.001$<br>[1.245, 1.384]	$z=20.27$, $p<0.001$<br>[0.759, 0.980]	$z=47.77$, $p<0.001$<br>[1.249, 1.391]
After	$z=29.12$, $p<0.001$<br>[0.124, 0.148]	$z=4.45$, $p<0.001$<br>[0.015, 0.055]	$z=27.18$, $p<0.001$<br>[0.122, 0.147]
Display×After	$z=-188.22$, $p<0.001$<br>[−1.005, −0.977]	$z=-98.92$, $p<0.001$<br>[−0.860, −0.816]	$z=-187.94$, $p<0.001$<br>[−1.005, −0.977]
PostAge	$z=-324.02$, $p<0.001$<br>[−0.803, −0.790]	$z=-171.95$, $p<0.001$<br>[−0.812, −0.788]	$z=-274.71$, $p<0.001$<br>[−0.803, −0.788]
Politics	$z=16.73$, $p<0.001$<br>[0.534, 0.728]		
Display×Politics	$z=-8.17$, $p<0.001$<br>[−0.575, −0.299]		
After×Politics	$z=-14.52$, $p<0.001$<br>[−0.124, −0.087]		
ATT×Politics	$z=15.35$, $p<0.001$<br>[0.128, 0.179]		
Intercept	$z=59.06$, $p<0.001$<br>[1.080, 1.179]	$z=57.87$, $p<0.001$<br>[1.692, 1.850]	$z=57.33$, $p<0.001$<br>[1.073, 1.174]
Post-level RE	✓	✓	✓
#Observations	654,400	167,040	487,360

	Interaction	With science	Without science
Display	$z=44.89$, $p<0.001$<br>[1.058, 1.187]	$z=26.89$, $p<0.001$<br>[1.523, 1.845]	$z=44.89$, $p<0.001$<br>[1.058, 1.187]
After	$z=21.40$, $p<0.001$<br>[0.082, 0.104]	$z=14.85$, $p<0.001$<br>[0.149, 0.212]	$z=21.64$, $p<0.001$<br>[0.085, 0.108]
Display×After	$z=-199.20$, $p<0.001$<br>[−0.961, −0.937]	$z=-74.71$, $p<0.001$<br>[−0.984, −0.918]	$z=-198.51$, $p<0.001$<br>[−0.961, −0.937]
PostAge	$z=-324.07$, $p<0.001$<br>[−0.803, −0.790]	$z=-108.95$, $p<0.001$<br>[−0.787, −0.751]	$z=-305.21$, $p<0.001$<br>[−0.807, −0.794]
Science	$z=-23.11$, $p<0.001$<br>[−1.214, −0.970]		
Display×Science	$z=8.31$, $p<0.001$<br>[0.387, 0.735]		
After×Science	$z=10.88$, $p<0.001$<br>[0.087, 0.141]		
ATT×Science	$z=-0.18$, $p=0.856$<br>[−0.038, 0.033]		
Intercept	$z=81.20$, $p<0.001$<br>[1.397, 1.489]	$z=8.39$, $p<0.001$<br>[0.257, 0.484]	$z=80.94$, $p<0.001$<br>[1.394, 1.486]
Post-level RE	✓	✓	✓
#Observations	654,400	89,600	564,800

Supplementary Note 8: Analysis of Overall Reduction

Table S33 shows the results of two-tailed KS tests for overall reduction of reposts across post ages.

Table S33: The results of two-tailed KS tests for overall reduction of reposts across post ages.

Post age	Overall reduction (mean ratio)	KS	p-value
2	–0.523	0.143	$<$ 0.001
4	–0.461	0.121	$<$ 0.001
6	–0.409	0.103	$<$ 0.001
8	–0.365	0.089	$<$ 0.001
10	–0.326	0.077	$<$ 0.001
12	–0.289	0.067	$<$ 0.001
14	–0.253	0.057	$<$ 0.001
16	–0.218	0.047	$<$ 0.001
18	–0.184	0.039	$<$ 0.001
20	–0.149	0.030	$<$ 0.001
22	–0.113	0.022	$<$ 0.001
24	–0.077	0.015	$<$ 0.001
26	–0.058	0.011	0.024
28	–0.045	0.008	0.178
30	–0.032	0.006	0.518
32	–0.021	0.004	0.928
34	–0.011	0.002	1.000
36	0.000	0.000	1.000

Supplementary Note 9: Analysis of Reposting Mechanisms

9.1 Collection of Reposter Information

We further examined how the display of community notes influenced different types of reposters. Due to the rate limits of the X (formerly Twitter) API, collecting comprehensive information on all reposters in our dataset was challenging. Given this, we randomly selected 2,000 posts with displayed notes and 2,000 posts without displayed notes to analyze the heterogeneity in the treatment effects of community notes among different types of reposters.

For each post, we used the X (formerly Twitter) Pro API's search/all endpoint to gather information about reposters during each hour within a time window of -4 to $+12$ hours relative to the post's publication. Reposts from the pre-display period (-4h until the note appears) are used to assess pre-trends, while the ATT estimates are identified from reposts occurring after the display of community notes. Due to API rate limits, we sampled reposters randomly within a 5-minute interval for each one-hour time block. Additionally, for each identified reposter, we used the X (formerly Twitter) Pro API's count/all endpoint to retrieve the total number of interactions (reposts) the reposter had with the original poster of the post over the 28 days *preceding* the publication of the post targeted by a community note. The metric thus does not include interactions with the post itself prior to the addition of a note. Based on this data, we construct a dummy variable set to 1 if the reposter had interacted with the creator of the post prior to reposting the specific post that was flagged with a community note.

Moreover, we used the dataset from [21] to assign each reposter a partisan score, which reflects the extent to which a user tends to follow politicians from the left or right of the political spectrum, and a misinformation exposure score, which measures the likelihood that the politicians and public organizations followed by the user disseminate false information. A reposter is classified as right-leaning if their partisan score is greater than 0, and left-leaning if their partisan score is less than 0. Similarly, a reposter is considered to have high exposure to misinformation if their misinformation

exposure score exceeds 0.5, and low exposure if the score is 0.5 or below.

We successfully collected 222,068 reposts with reposter information for 3,163 source posts. Each reposter has four characteristics: (i) the prior interaction status, indicating whether the reposter had prior interactions with the same author of the misleading post, (ii) the verified status of the reposter, (iii) the political leaning of the reposter, and (iv) the exposure score of the reposter to misinformation. The distributions of the propensity score in the collected posts in the treatment and control groups maintained balanced ($t = 0.113, p = 0.910$; see Figure S9).

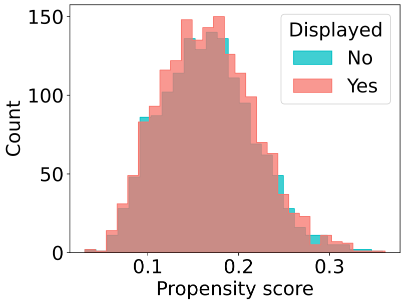

Figure S9: **Propensity score distributions.** The distributions of the propensity score for collected posts in the treatment and control groups are shown in the plot, respectively.

9.2 Reposter Dataset

An overview of the dataset for the reposter analysis is shown in Table S34. For political orientations, we defined two binary variables, i.e., ***LeftLeaning*** and ***RightLeaning***, to indicate whether the (re)poster is politically left(right)-leaning (= 1) or not (= 0). Moreover, we defined another two binary variables, i.e., ***HighMisinfoExposure*** and ***LowMisinfoExposure***, to indicate whether the (re)poster has high(low) exposure to misinformation (= 1) or not (= 0). Additionally, ***Verified*** is a binary variable showing that whether the reposter is verified (= 1) or not (= 0). ***PriorInteraction*** is a binary variable indicating whether the reposter had prior interactions with the authors of misleading posts (= 1) or not (= 0).

Table S34: Overview of the dataset for reposter analysis. Descriptive statistics are reported for the whole dataset (column (1)), the treated posts that received at least one displayed helpful note (column (2)), and the control posts that did not received a displayed helpful note during our observation period (column (3)).

		Posts with displayed notes	
	All	Yes	No
#Reposts	222,068	106,797	115,271
#Posts	3,163	1,676	1,487
#Posters	1,979	1,094	1,115
Poster characteristics			
LeftLeaning	25.5 %	24.6 %	26.4 %
RightLeaning	30.9 %	31.6 %	30.2 %
HighMisinfoExposure	37.3 %	38.2 %	36.2 %
LowMisinfoExposure	20.6 %	19.3 %	22.2 %
#Reposts across reposter characteristics			
PriorInteraction	122,180	54,697	67,483
Verified	13,461	6,253	7,208
LeftLeaning	46,916	21,532	25,384
RightLeaning	67,694	32,237	35,457
HighMisinfoExposure	86,867	41,587	45,280
LowMisinfoExposure	135,201	65,210	69,991

9.3 Parallel Trend and Treatment Effect

To validate the robustness of the parallel trend and the treatment effect of community notes, we repeated our multi-period and two-period DiD estimations for the collected post subset. Table S35 reports the results for multi-period DiD estimation, showing that the DiD terms during before-display period were not statistically significantly different from zero (each $p > 0.01$). This indicates that the changes of reposts in the treatment group and control group for the post subset remained parallel before the display of community notes. Column (1) of Table S37 reports the results for two-period DiD estimation, where the ATT was estimated as –0.633 (99% CI: [–0.651, –0.614]; $z = -50.57$, $p < 0.001$). This suggests that the treatment effect of community notes in the subset of posts for reposter analysis is consistent with the overall treatment effect (i.e., –0.612 (99% CI: [–0.617, –0.608]; $z = -211.71$, $p < 0.001$). Additionally, we expanded the two-period DiD estimation by incorporating the political leanings and misinformation exposures of posters. As shown in Column (2) of Table S37, the ATT estimate remained identical to that in Column (1).

Table S35: Regression results for parallel test and multi-period ATTs estimation in the subset of posts for reposter analysis. Post-specific random effects are included. Reported are coefficient estimates with standard errors in parentheses. * $p < 0.01$, ** $p < 0.005$, *** $p < 0.001$. Exact z statistics, p values and 99% CIs are reported in Table S36.

	Multi-period
Display	0.588***
	(0.071)
Before:4	0.242***
	(0.038)
Before:3	0.145***
	(0.036)
Before:2	0.044
	(0.034)
After:1	–0.131***
	(0.035)
After:2	–0.170***
	(0.037)
After:3	–0.227***
	(0.040)
After:4	–0.240***
	(0.043)
After:5	–0.257***
	(0.046)
After:6	–0.329***
	(0.050)
After:7	–0.357***
	(0.055)
After:8	–0.440***
	(0.060)
After:9	–0.472***
	(0.064)
After:10	–0.520***
	(0.069)
After:11	–0.596***
	(0.074)
After:12	–0.617***
	(0.079)

	Continued
Display×Before:4	0.038
	(0.044)
Display×Before:3	0.020
	(0.044)
Display×Before:2	0.064
	(0.044)
Display×After:1	–0.619***
	(0.047)
Display×After:2	–0.879***
	(0.048)
Display×After:3	–0.913***
	(0.049)
Display×After:4	–1.032***
	(0.049)
Display×After:5	–1.045***
	(0.049)
Display×After:6	–1.044***
	(0.050)
Display×After:7	–1.096***
	(0.051)
Display×After:8	–1.029***
	(0.051)
Display×After:9	–1.061***
	(0.052)
Display×After:10	–1.084***
	(0.052)
Display×After:11	–1.039***
	(0.053)
Display×After:12	–1.065***
	(0.054)
PostAge	–0.170***
	(0.042)

	Continued
Intercept	0.160*
	(0.059)
Post-level RE	✓
#Observations	50,608
#Posts	3,163

Table S36: Details of z statistics, p values and 99% CIs (in brackets) for the coefficient estimates reported in Table S35.

	Multi-period
Display	z =8.23, $p < 0.001$ [0.404, 0.771]
Before:4	z =6.43, $p < 0.001$ [0.145, 0.338]
Before:3	z =4.09, $p < 0.001$ [0.054, 0.237]
Before:2	z =1.29, p =0.197 [−0.044, 0.133]
After:1	z =−3.72, $p < 0.001$ [−0.222, −0.040]
After:2	z =−4.59, $p < 0.001$ [−0.266, −0.075]
After:3	z =−5.69, $p < 0.001$ [−0.329, −0.124]
After:4	z =−5.61, $p < 0.001$ [−0.351, −0.130]
After:5	z =−5.53, $p < 0.001$ [−0.376, −0.137]
After:6	z =−6.52, $p < 0.001$ [−0.459, −0.199]
After:7	z =−6.50, $p < 0.001$ [−0.498, −0.215]
After:8	z =−7.39, $p < 0.001$ [−0.593, −0.287]
After:9	z =−7.35, $p < 0.001$ [−0.638, −0.307]
After:10	z =−7.51, $p < 0.001$ [−0.698, −0.342]
After:11	z =−8.01, $p < 0.001$ [−0.787, −0.404]
After:12	z =−7.76, $p < 0.001$ [−0.821, −0.412]

	Continued
Display×Before:4	z =0.88, p =0.380 [−0.074, 0.151]
Display×Before:3	z =0.45, p =0.653 [−0.094, 0.133]
Display×Before:2	z =1.44, p =0.149 [−0.050, 0.178]
Display×After:1	z =−13.24, $p < 0.001$ [−0.739, −0.498]
Display×After:2	z =−18.35, $p < 0.001$ [−1.003, −0.756]
Display×After:3	z =−18.82, $p < 0.001$ [−1.038, −0.788]
Display×After:4	z =−20.97, $p < 0.001$ [−1.159, −0.905]
Display×After:5	z =−21.16, $p < 0.001$ [−1.173, −0.918]
Display×After:6	z =−20.91, $p < 0.001$ [−1.173, −0.916]
Display×After:7	z =−21.62, $p < 0.001$ [−1.226, −0.965]
Display×After:8	z =−20.10, $p < 0.001$ [−1.161, −0.897]
Display×After:9	z =−20.49, $p < 0.001$ [−1.194, −0.928]
Display×After:10	z =−20.67, $p < 0.001$ [−1.219, −0.949]
Display×After:11	z =−19.56, $p < 0.001$ [−1.176, −0.903]
Display×After:12	z =−19.85, $p < 0.001$ [−1.203, −0.926]
PostAge	z =−4.06, $p < 0.001$ [−0.279, −0.062]

	Continued
Intercept	z =2.69, p =0.007 [0.007, 0.312]
Post-level RE	✓
#Observations	50,608
#Posts	3,163

Table S37: Regression results for two-period ATT estimation in the subset of posts for reposter analysis. Post-specific random effects are included. Reported are coefficient estimates with standard errors in parentheses. * $p < 0.01$, ** $p < 0.005$, *** $p < 0.001$. Exact z statistics, p values and 99% CIs are reported in Table S38.

	(1) Main	(2) Extended
Display	0.631***	0.621***
	(0.067)	(0.065)
After	−0.007	−0.009
	(0.019)	(0.019)
Display×After	−1.002***	−1.002***
	(0.020)	(0.020)
PostAge	−0.590***	−0.588***
	(0.011)	(0.011)
Verified		0.531***
		(0.084)
AccountAge		−0.103**
		(0.036)
Followers		0.268***
		(0.041)
Followees		0.079*
		(0.031)
Words		−0.066
		(0.034)
Media		0.435***
		(0.075)
Economy		−0.214
		(0.099)
Health		0.211
		(0.104)
Politics		0.002
		(0.077)
Science		−0.339***
		(0.101)
Positive		−0.068
		(0.041)
Negative		0.133***
		(0.040)
MFRO		−0.141***
		(0.032)
$\text{LeftLeaning}_{\text{poster}}$		0.268
		(0.183)
$\text{RightLeaning}_{\text{poster}}$		0.397
		(0.187)
$\text{HighMisinfoExposure}_{\text{poster}}$		−0.124
		(0.184)
$\text{LowMisinfoExposure}_{\text{poster}}$		−0.324
		(0.193)
Intercept	−0.096	−0.885***
	(0.050)	(0.111)
Post-level RE	✓	✓
#Observations	50,608	50,608
#Posts	3,163	3,163

Table S38: Details of z statistics, p values and 99% CIs (in brackets) for the coefficient estimates reported in Table S37.

	(1) Main	(2) Extended
Display	z =9.43, p < 0.001 [0.459, 0.803]	z =9.55, p < 0.001 [0.453, 0.788]
After	z =−0.35, p =0.728 [−0.055, 0.042]	z =−0.45, p =0.652 [−0.057, 0.040]
Display×After	z =−50.57, p < 0.001 [−1.053, −0.951]	z =−50.58, p < 0.001 [−1.053, −0.951]
PostAge	z =−51.93, p < 0.001 [−0.620, −0.561]	z =−51.86, p < 0.001 [−0.618, −0.559]
Verified		z =6.30, p < 0.001 [0.314, 0.748]
AccountAge		z =−2.86, p =0.004 [−0.195, −0.010]
Followers		z =6.47, p < 0.001 [0.161, 0.374]
Followees		z =2.58, p =0.010 [0.000, 0.158]
Words		z =−1.94, p =0.052 [−0.154, 0.022]
Media		z =5.78, p < 0.001 [0.241, 0.629]
Economy		z =−2.17, p =0.030 [−0.469, 0.040]
Health		z =2.03, p =0.043 [−0.057, 0.478]
Politics		z =0.03, p =0.974 [−0.195, 0.200]
Science		z =−3.36, p < 0.001 [−0.598, −0.079]
Positive		z =−1.65, p =0.099 [−0.175, 0.038]
Negative		z =3.33, p < 0.001 [0.030, 0.236]
MFRO		z =−4.44, p < 0.001 [−0.222, −0.059]
$\text{LeftLeaning}_{\text{poster}}$		z =1.46, p =0.143 [−0.203, 0.739]
$\text{RightLeaning}_{\text{poster}}$		z =2.13, p =0.033 [−0.084, 0.877]
$\text{HighMisinfoExposure}_{\text{poster}}$		z =−0.67, p =0.501 [−0.597, 0.349]
$\text{LowMisinfoExposure}_{\text{poster}}$		z =−1.68, p =0.093 [−0.820, 0.173]
Intercept	z =−1.93, p =0.054 [−0.225, 0.032]	z =−7.94, p < 0.001 [−1.172, −0.598]
Post-level RE	✓	✓
#Observations	50,608	50,608
#Posts	3,163	3,163

9.4 ATTs Across Reposter Characteristics

Table S39 reports the regression results across the four reposter characteristics. The ATT estimate for connected reposters was –0.586 (99% CI: [–0.608, –0.561]; $z = -40.10$, $p < 0.001$); the ATT estimate for disconnected reposters was –0.682 (99% CI: [–0.703, –0.660]; $z = -43.70$, $p < 0.001$). The ATT estimate for verified reposters was –0.595 (99% CI: [–0.640, –0.545]; $z = -20.00$, $p < 0.001$); the ATT estimate for non-verified reposters was –0.634 (99% CI: [–0.653, –0.615]; $z = -50.24$, $p < 0.001$). The ATT estimate for left-leaning reposters was –0.625 (99% CI: [–0.654, –0.595]; $z = -32.19$, $p < 0.001$); the ATT estimate for right-leaning reposters was –0.626 (99% CI: [–0.652, –0.598]; $z = -35.34$, $p < 0.001$). The ATT estimate for high-misinfo-exposure reposters was –0.633 (99% CI: [–0.655, –0.609]; $z = -40.63$, $p < 0.001$); the ATT estimate for low-misinfo-exposure reposters was –0.628 (99% CI: [–0.649, –0.606]; $z = -44.24$, $p < 0.001$). The efficacy of community notes was 16.4% larger for disconnected reposters compared to connected reposters.

Table S39: Regression results for two-period ATT estimations across reposter characteristics. Post-specific random effects are included. Reported are coefficient estimates with standard errors in parentheses. * $p < 0.01$, ** $p < 0.005$, *** $p < 0.001$. Exact z statistics, p values and 99% CIs are reported in Table S40.

	PriorInteraction		Verified		Political leaning		Misinformation exposure	
	(1) Yes	(2) No	(3) Yes	(4) No	(5) Left	(6) Right	(7) High	(8) Low
Display	0.334***	0.865***	0.463***	0.637***	0.484***	0.742***	0.688***	0.604***
	(0.078)	(0.075)	(0.082)	(0.067)	(0.078)	(0.079)	(0.074)	(0.069)
After	−0.043	0.072**	−0.111*	−0.001	−0.017	−0.005	−0.005	−0.006
	(0.021)	(0.025)	(0.042)	(0.019)	(0.029)	(0.027)	(0.024)	(0.021)
Display × After	−0.881***	−1.145***	−0.904***	−1.006***	−0.981***	−0.984***	−1.001***	−0.989***
	(0.022)	(0.026)	(0.045)	(0.020)	(0.030)	(0.028)	(0.025)	(0.022)
PostAge	−0.632***	−0.502***	−0.570***	−0.582***	−0.557***	−0.579***	−0.577***	−0.547***
	(0.013)	(0.015)	(0.026)	(0.012)	(0.018)	(0.016)	(0.015)	(0.013)
Intercept	−0.988***	−1.275***	−3.013***	−0.181***	−1.965***	−1.837***	−1.251***	−0.696***
	(0.058)	(0.057)	(0.071)	(0.050)	(0.061)	(0.062)	(0.056)	(0.052)
Post-level RE	✓	✓	✓	✓	✓	✓	✓	✓
#Observations	50,608	50,608	50,608	50,608	50,608	50,608	50,608	50,608

Table S40: Details of z statistics, p values and 99% CIs (in brackets) for the coefficient estimates reported in Table S39.

	PriorInteraction		Verified		Political leaning		Misinformation exposure	
	(1) Yes	(2) No	(3) Yes	(4) No	(5) Left	(6) Right	(7) High	(8) Low
Display	z =4.31, $p < 0.001$ [0.134, 0.534]	z =11.50, $p < 0.001$ [0.671, 1.058]	z =5.63, $p < 0.001$ [0.251, 0.674]	z =9.44, $p < 0.001$ [0.463, 0.810]	z =6.20, $p < 0.001$ [0.283, 0.685]	z =9.35, $p < 0.001$ [0.538, 0.947]	z =9.35, $p < 0.001$ [0.499, 0.877]	z =8.71, $p < 0.001$ [0.425, 0.782]
After	z =−2.06, p =0.039 [−0.096, 0.011]	z =2.84, p =0.004 [0.007, 0.136]	z =−2.66, p =0.008 [−0.219, −0.003]	z =−0.04, p =0.966 [−0.050, 0.048]	z =−0.58, p =0.562 [−0.091, 0.058]	z =−0.19, p =0.846 [−0.074, 0.064]	z =−0.22, p =0.828 [−0.066, 0.056]	z =−0.27, p =0.784 [−0.061, 0.049]
Display×After	z =−40.10, $p < 0.001$ [−0.937, −0.824]	z =−43.70, $p < 0.001$ [−1.213, −1.078]	z =−20.00, $p < 0.001$ [−1.021, −0.788]	z =−50.24, $p < 0.001$ [−1.057, −0.954]	z =−32.19, $p < 0.001$ [−1.060, −0.903]	z =−35.34, $p < 0.001$ [−1.055, −0.912]	z =−40.63, $p < 0.001$ [−1.065, −0.938]	z =−44.24, $p < 0.001$ [−1.047, −0.932]
PostAge	z =−48.31, $p < 0.001$ [−0.665, −0.598]	z =−33.69, $p < 0.001$ [−0.541, −0.464]	z =−21.69, $p < 0.001$ [−0.638, −0.502]	z =−50.60, $p < 0.001$ [−0.611, −0.552]	z =−30.93, $p < 0.001$ [−0.604, −0.511]	z =−35.40, $p < 0.001$ [−0.622, −0.537]	z =−39.77, $p < 0.001$ [−0.614, −0.539]	z =−42.26, $p < 0.001$ [−0.581, −0.514]
Intercept	z =−16.99, $p < 0.001$ [−1.138, −0.838]	z =−22.25, $p < 0.001$ [−1.423, −1.128]	z =−42.59, $p < 0.001$ [−3.195, −2.831]	z =−3.59, $p < 0.001$ [−0.310, −0.051]	z =−32.14, $p < 0.001$ [−2.122, −1.807]	z =−29.82, $p < 0.001$ [−1.995, −1.678]	z =−22.33, $p < 0.001$ [−1.396, −1.107]	z =−13.33, $p < 0.001$ [−0.830, −0.561]
Post-level RE	✓	✓	✓	✓	✓	✓	✓	✓
#Observations	50,608	50,608	50,608	50,608	50,608	50,608	50,608	50,608

9.5 Sensitivity Across Poster and Reposter Characteristics

Figure S10–Figure S13 show the estimated ATTs across different groups of reposters based on posts separated by poster and post characteristics. We found that the trend of larger efficacy of community notes for disconnected reposters than for connected reposters was present across all poster and post characteristics.

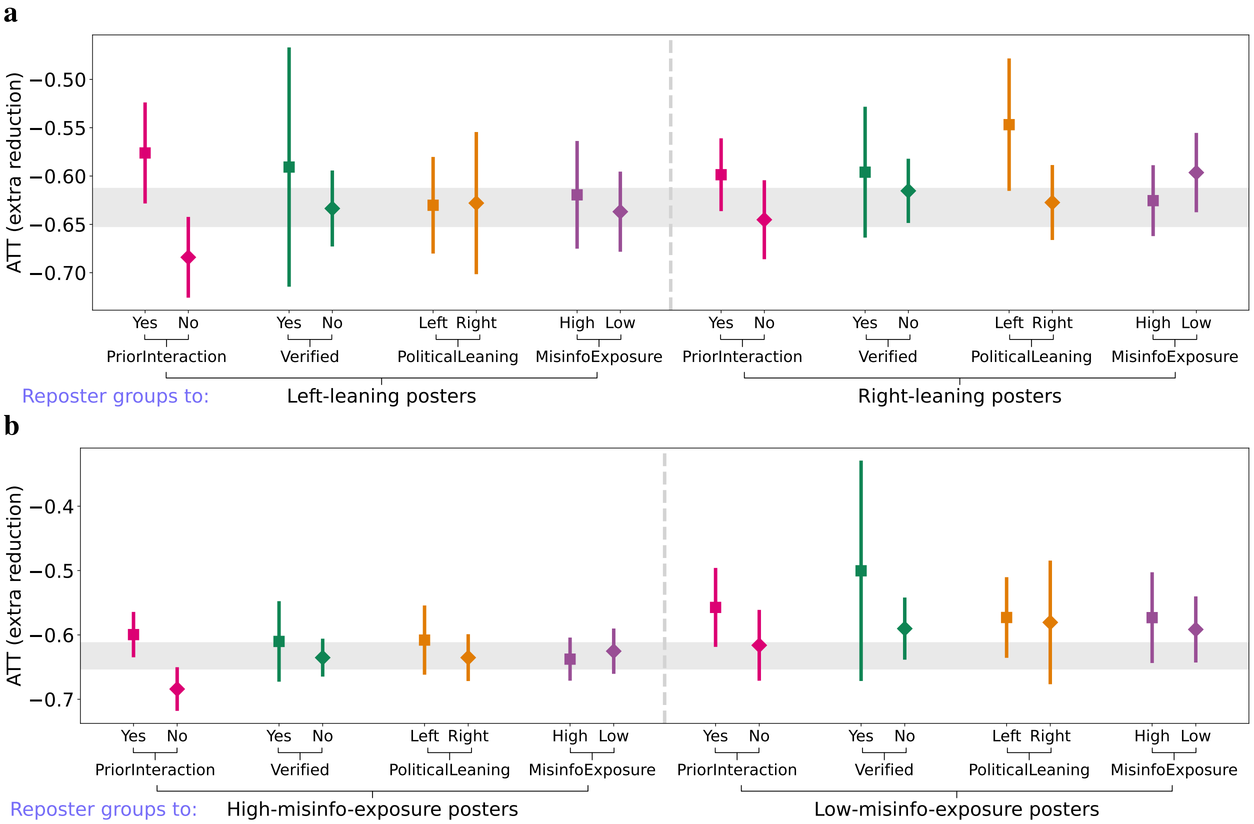

Figure S10: **The estimated ATTs across different groups of reposters based on posts separated by poster characteristics.** **(a)** Left-leaning vs. right-leaning posters. **(b)** High-misinfo-exposure vs. low-misinfo-exposure posters. Shown are mean values with 99% CIs. The full estimation results are reported in Table S41–Table S47.

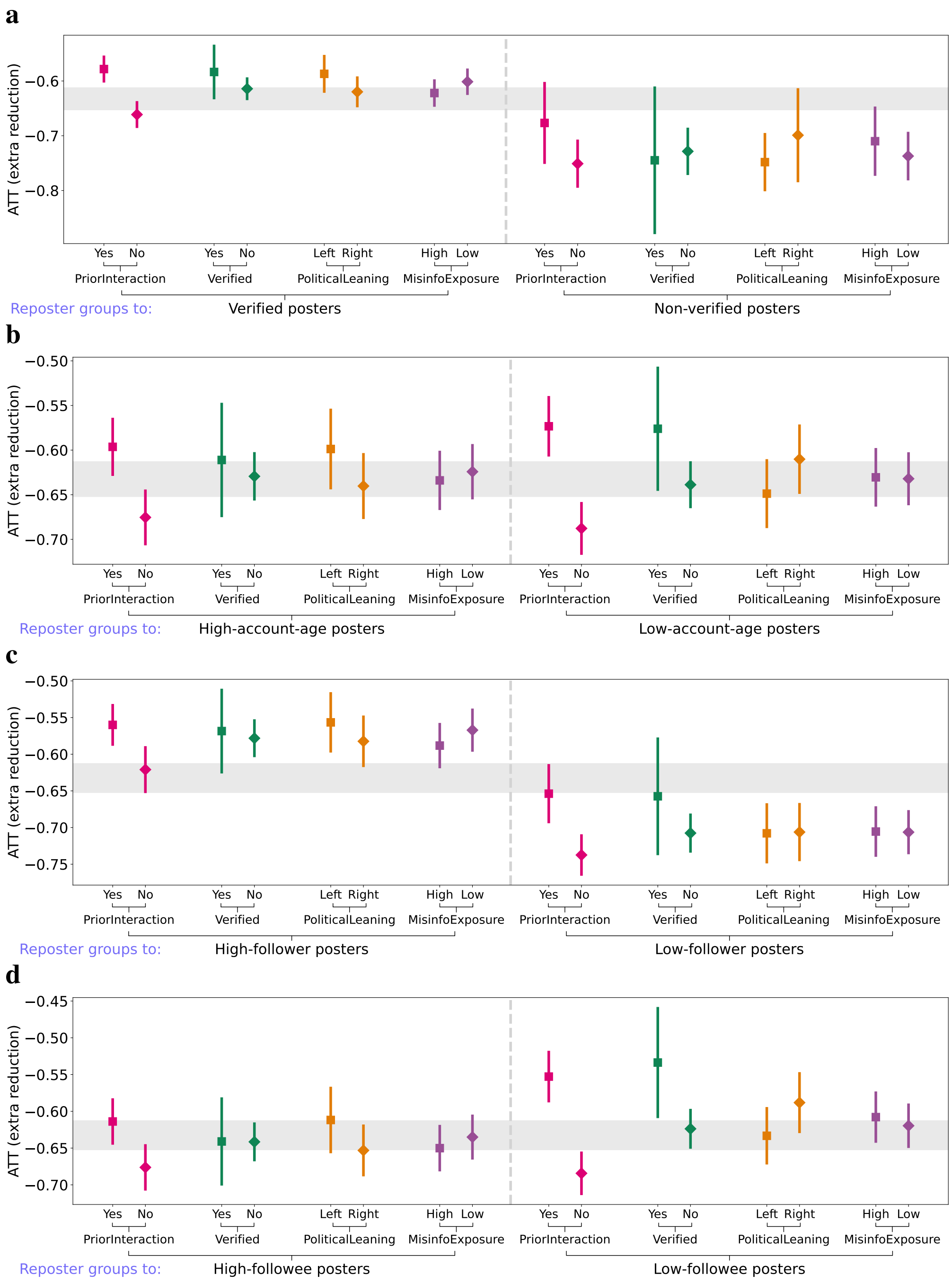

Figure S11: **The estimated ATTs across different groups of reposters based on posts separated by poster characteristics.** **(a)** Verified vs. non-verified posters. **(b)** High-account-age vs. low-account-age posters. **(c)** High-follower vs. low-follower posters. **(d)** High-followee vs. low-followee posters. Shown are mean values with 99% CIs. The full estimation results are reported in Table S49–Table S63.

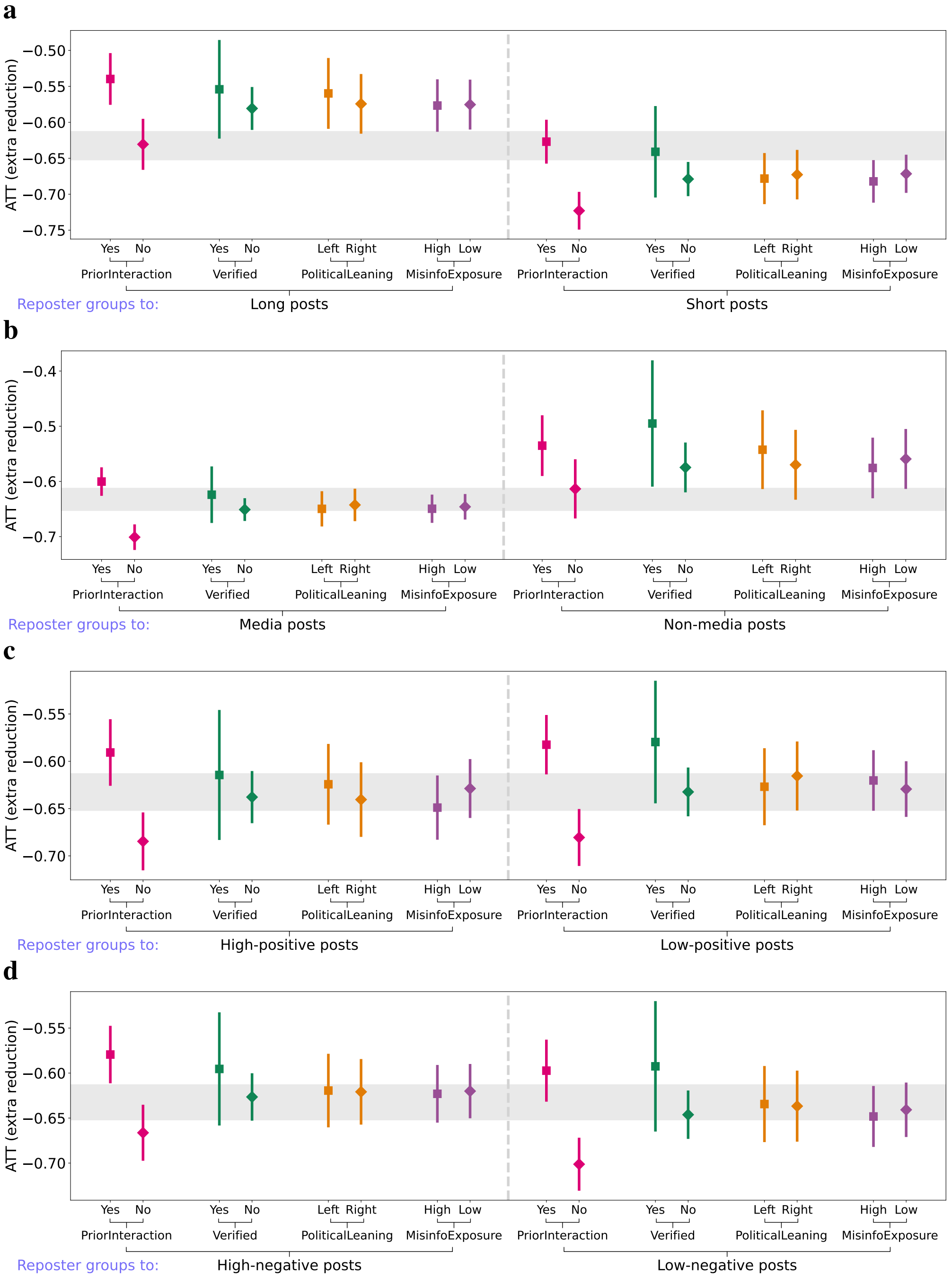

Figure S12: **The estimated ATTs across different groups of reposters based on posts separated by post characteristics.** **(a)** Long vs. short posts. **(b)** Media vs. non-media posts. **(c)** High-positive vs. low-positive posts. **(d)** High-negative vs. low-negative posts. Shown are mean values with 99% CIs. The full estimation results are reported in Table S65–Table S79.

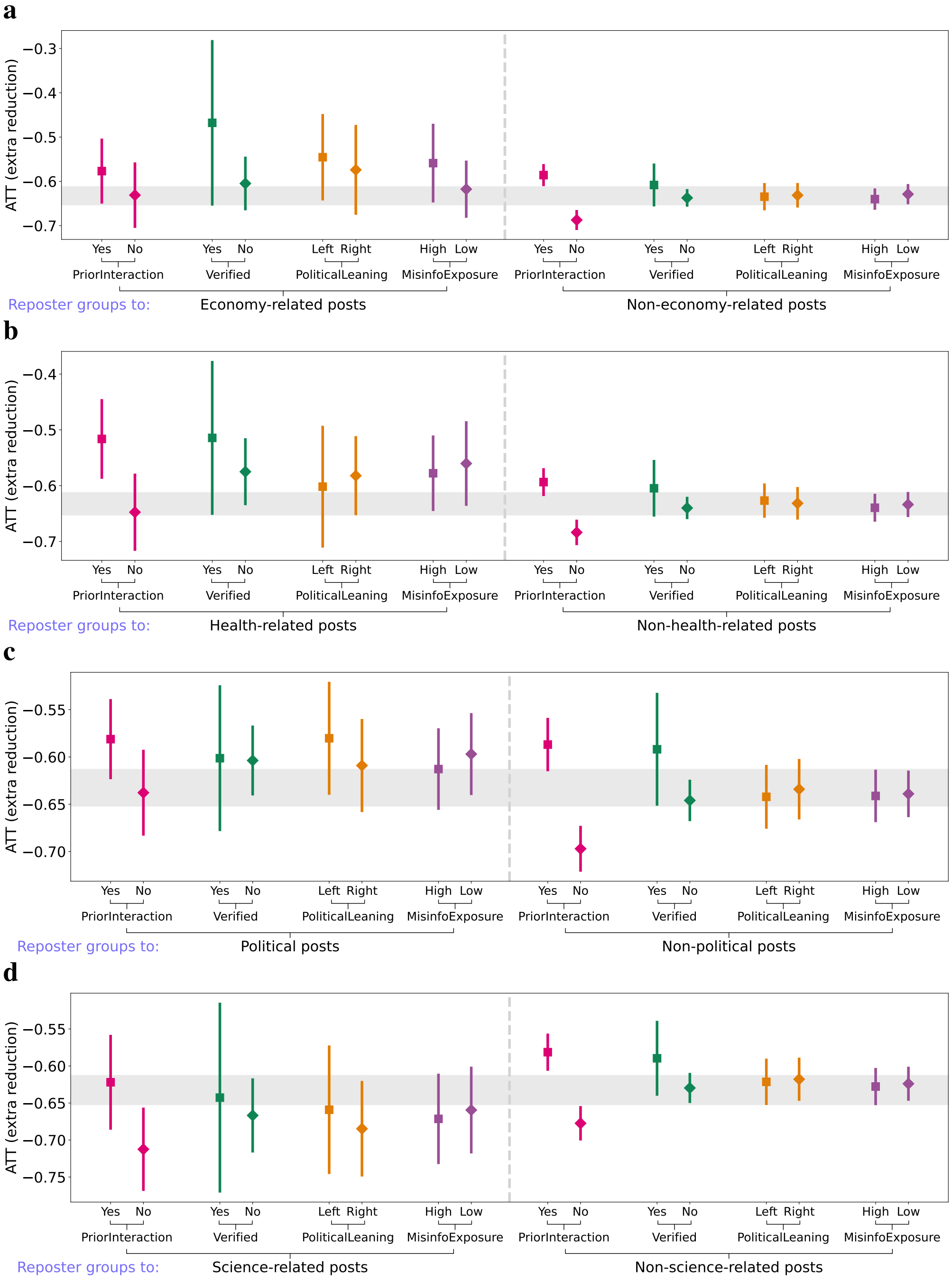

Figure S13: **The estimated ATTs across different groups of reposters based on posts separated by post characteristics.** **(a)** Economy-related vs. non-economy-related posts. **(b)** Health-related vs. non-health-related posts. **(c)** Political vs. non-political posts. **(d)** Science-related vs. non-science-related posts. Shown are mean values with 99% CIs. The full estimation results are reported in Table S81–Table S95.

Table S41: Regression results for two-period ATT estimations, based on posts from left-leaning posters and across reposter characteristics. Post-specific random effects are included. Reported are coefficient estimates with standard errors in parentheses. * $p < 0.01$, ** $p < 0.005$, *** $p < 0.001$. Exact z statistics, p values and 99% CIs are reported in Table S42.

	PriorInteraction		Verified		Political leaning		Misinformation exposure	
	(1)	(2)	(3)	(4)	(5)	(6)	(7)	(8)
	Yes	No	Yes	No	Left	Right	High	Low
Display	0.314	0.958***	0.470**	0.680***	0.419**	1.053***	0.823***	0.624***
	(0.145)	(0.146)	(0.165)	(0.130)	(0.144)	(0.155)	(0.139)	(0.133)
After	−0.053	0.092	−0.087	0.009	−0.032	0.037	0.018	−0.001
	(0.045)	(0.049)	(0.104)	(0.039)	(0.049)	(0.073)	(0.054)	(0.042)
Display × After	−0.858***	−1.152***	−0.893***	−1.004***	−0.995***	−0.989***	−0.966***	−1.013***
	(0.048)	(0.051)	(0.117)	(0.042)	(0.052)	(0.077)	(0.057)	(0.044)
PostAge	−0.695***	−0.555***	−0.583***	−0.620***	−0.627***	−0.567***	−0.582***	−0.606***
	(0.029)	(0.030)	(0.064)	(0.024)	(0.031)	(0.045)	(0.034)	(0.026)
Intercept	−1.268***	−1.325***	−3.507***	−0.365***	−1.280***	−2.887***	−1.818***	−0.640***
	(0.108)	(0.110)	(0.150)	(0.096)	(0.109)	(0.130)	(0.108)	(0.099)
Post-level RE	✓	✓	✓	✓	✓	✓	✓	✓
#Observations	12,896	12,896	12,896	12,896	12,896	12,896	12,896	12,896

Table S42: Details of z statistics, p values and 99% CIs (in brackets) for the coefficient estimates reported in Table S41.

	PriorInteraction		Verified		Political leaning		Misinformation exposure	
	(1)	(2)	(3)	(4)	(5)	(6)	(7)	(8)
	Yes	No	Yes	No	Left	Right	High	Low
Display	z =2.17, p =0.030<br>[−0.059, 0.687]	z =6.54, p < 0.001<br>[0.581, 1.335]	z =2.85, p =0.004<br>[0.046, 0.895]	z =5.23, p < 0.001<br>[0.345, 1.015]	z =2.91, p =0.004<br>[0.048, 0.791]	z =6.79, p < 0.001<br>[0.654, 1.453]	z =5.93, p < 0.001<br>[0.465, 1.180]	z =4.70, p < 0.001<br>[0.282, 0.966]
After	z =−1.18, p =0.240<br>[−0.168, 0.063]	z =1.88, p =0.061<br>[−0.034, 0.218]	z =−0.83, p =0.404<br>[−0.354, 0.181]	z =0.22, p =0.822<br>[−0.093, 0.111]	z =−0.65, p =0.517<br>[−0.159, 0.095]	z =0.51, p =0.613<br>[−0.150, 0.224]	z =0.33, p =0.738<br>[−0.121, 0.158]	z =−0.03, p =0.973<br>[−0.109, 0.107]
Display×After	z =−17.96, p < 0.001<br>[−0.981, −0.735]	z =−22.48, p < 0.001<br>[−1.284, −1.020]	z =−7.62, p < 0.001<br>[−1.195, −0.591]	z =−24.15, p < 0.001<br>[−1.111, −0.897]	z =−18.99, p < 0.001<br>[−1.130, −0.860]	z =−12.91, p < 0.001<br>[−1.186, −0.791]	z =−17.03, p < 0.001<br>[−1.112, −0.820]	z =−22.89, p < 0.001<br>[−1.127, −0.899]
PostAge	z =−23.76, p < 0.001<br>[−0.770, −0.619]	z =−18.66, p < 0.001<br>[−0.632, −0.479]	z =−9.17, p < 0.001<br>[−0.746, −0.419]	z =−25.55, p < 0.001<br>[−0.683, −0.558]	z =−19.99, p < 0.001<br>[−0.708, −0.546]	z =−12.70, p < 0.001<br>[−0.681, −0.452]	z =−17.19, p < 0.001<br>[−0.670, −0.495]	z =−23.26, p < 0.001<br>[−0.673, −0.539]
Intercept	z =−11.72, p < 0.001<br>[−1.547, −0.989]	z =−12.01, p < 0.001<br>[−1.610, −1.041]	z =−23.32, p < 0.001<br>[−3.895, −3.120]	z =−3.80, p < 0.001<br>[−0.613, −0.118]	z =−11.79, p < 0.001<br>[−1.559, −1.000]	z =−22.24, p < 0.001<br>[−3.222, −2.553]	z =−16.86, p < 0.001<br>[−2.096, −1.541]	z =−6.50, p < 0.001<br>[−0.894, −0.387]
Post-level RE	✓	✓	✓	✓	✓	✓	✓	✓
#Observations	12,896	12,896	12,896	12,896	12,896	12,896	12,896	12,896

Table S43: Regression results for two-period ATT estimations, based on posts from right-leaning posters and across reposter characteristics. Post-specific random effects are included. Reported are coefficient estimates with standard errors in parentheses. * $p < 0.01$, ** $p < 0.005$, *** $p < 0.001$. Exact z statistics, p values and 99% CIs are reported in Table S44.

	PriorInteraction		Verified		Political leaning		Misinformation exposure	
	(1)	(2)	(3)	(4)	(5)	(6)	(7)	(8)
	Yes	No	Yes	No	Left	Right	High	Low
Display	0.089	0.838***	0.234	0.474***	0.438**	0.473***	0.490***	0.445***
	(0.138)	(0.133)	(0.134)	(0.121)	(0.138)	(0.130)	(0.125)	(0.127)
After	−0.002	0.056	−0.068	0.008	0.024	−0.012	−0.006	0.025
	(0.034)	(0.043)	(0.061)	(0.032)	(0.055)	(0.039)	(0.036)	(0.037)
Display × After	−0.913***	−1.036***	−0.906***	−0.955***	−0.791***	−0.987***	−0.982***	−0.907***
	(0.036)	(0.045)	(0.065)	(0.033)	(0.059)	(0.040)	(0.038)	(0.039)
PostAge	−0.634***	−0.517***	−0.652***	−0.594***	−0.530***	−0.618***	−0.610***	−0.552***
	(0.021)	(0.026)	(0.039)	(0.019)	(0.034)	(0.024)	(0.022)	(0.023)
Intercept	−0.457***	−1.178***	−2.208***	0.110	−2.252***	−0.741***	−0.499***	−0.716***
	(0.104)	(0.102)	(0.109)	(0.091)	(0.111)	(0.099)	(0.095)	(0.096)
Post-level RE	✓	✓	✓	✓	✓	✓	✓	✓
#Observations	15,648	15,648	15,648	15,648	15,648	15,648	15,648	15,648

Table S44: Details of z statistics, p values and 99% CIs (in brackets) for the coefficient estimates reported in Table S43.

	PriorInteraction		Verified		Political leaning		Misinformation exposure	
	(1)	(2)	(3)	(4)	(5)	(6)	(7)	(8)
	Yes	No	Yes	No	Left	Right	High	Low
Display	z =0.64, p =0.520	z =6.30, $p < 0.001$	z =1.74, p =0.082	z =3.93, $p < 0.001$	z =3.17, p =0.002	z =3.64, $p < 0.001$	z =3.91, $p < 0.001$	z =3.50, $p < 0.001$
	[−0.267, 0.445]	[0.496, 1.181]	[−0.112, 0.580]	[0.163, 0.784]	[0.083, 0.794]	[0.138, 0.808]	[0.168, 0.812]	[0.117, 0.772]
After	z =−0.07, p =0.942	z =1.28, p =0.199	z =−1.12, p =0.264	z =0.24, p =0.808	z =0.44, p =0.658	z =−0.31, p =0.756	z =−0.16, p =0.874	z =0.68, p =0.499
	[−0.090, 0.085]	[−0.056, 0.167]	[−0.224, 0.089]	[−0.074, 0.090]	[−0.118, 0.167]	[−0.111, 0.087]	[−0.099, 0.087]	[−0.071, 0.122]
Display×After	z =−25.10, $p < 0.001$	z =−23.22, $p < 0.001$	z =−13.96, $p < 0.001$	z =−28.54, $p < 0.001$	z =−13.51, $p < 0.001$	z =−24.47, $p < 0.001$	z =−25.87, $p < 0.001$	z =−23.02, $p < 0.001$
	[−1.007, −0.819]	[−1.151, −0.921]	[−1.074, −0.739]	[−1.042, −0.869]	[−0.942, −0.641]	[−1.091, −0.883]	[−1.080, −0.884]	[−1.009, −0.806]
PostAge	z =−29.74, $p < 0.001$	z =−20.25, $p < 0.001$	z =−16.69, $p < 0.001$	z =−30.88, $p < 0.001$	z =−15.61, $p < 0.001$	z =−26.05, $p < 0.001$	z =−27.48, $p < 0.001$	z =−24.14, $p < 0.001$
	[−0.688, −0.579]	[−0.583, −0.451]	[−0.753, −0.551]	[−0.643, −0.544]	[−0.618, −0.443]	[−0.679, −0.557]	[−0.667, −0.553]	[−0.611, −0.493]
Intercept	z =−4.41, $p < 0.001$	z =−11.53, $p < 0.001$	z =−20.20, $p < 0.001$	z =1.21, p =0.227	z =−20.25, $p < 0.001$	z =−7.50, $p < 0.001$	z =−5.26, $p < 0.001$	z =−7.42, $p < 0.001$
	[−0.724, −0.190]	[−1.442, −0.915]	[−2.489, −1.926]	[−0.124, 0.343]	[−2.539, −1.966]	[−0.996, −0.487]	[−0.744, −0.255]	[−0.964, −0.467]
Post-level RE	✓	✓	✓	✓	✓	✓	✓	✓
#Observations	15,648	15,648	15,648	15,648	15,648	15,648	15,648	15,648

Table S45: Regression results for two-period ATT estimations, based on posts from high-misinformation-exposure posters and across reposter characteristics. Post-specific random effects are included. Reported are coefficient estimates with standard errors in parentheses. * $p < 0.01$, ** $p < 0.005$, *** $p < 0.001$. Exact z statistics, p values and 99% CIs are reported in Table S46.

	PriorInteraction		Verified		Political leaning		Misinformation exposure	
	(1)	(2)	(3)	(4)	(5)	(6)	(7)	(8)
	Yes	No	Yes	No	Left	Right	High	Low
Display	0.104	0.896***	0.291	0.506***	0.503***	0.431***	0.484***	0.507***
	(0.125)	(0.122)	(0.126)	(0.109)	(0.126)	(0.122)	(0.115)	(0.114)
After	−0.050	0.083	−0.080	−0.011	−0.027	−0.026	−0.017	−0.006
	(0.032)	(0.040)	(0.058)	(0.030)	(0.051)	(0.037)	(0.034)	(0.035)
Display × After	−0.915***	−1.152***	−0.942***	−1.009***	−0.937***	−1.009***	−1.015***	−0.982***
	(0.034)	(0.042)	(0.062)	(0.031)	(0.053)	(0.039)	(0.036)	(0.036)
PostAge	−0.623***	−0.524***	−0.633***	−0.588***	−0.549***	−0.597***	−0.593***	−0.552***
	(0.020)	(0.024)	(0.037)	(0.018)	(0.031)	(0.023)	(0.021)	(0.021)
Intercept	−0.536***	−1.229***	−2.397***	0.060	−2.132***	−0.949***	−0.638***	−0.686***
	(0.094)	(0.094)	(0.104)	(0.082)	(0.101)	(0.093)	(0.088)	(0.087)
Post-level RE	✓	✓	✓	✓	✓	✓	✓	✓
#Observations	18,864	18,864	18,864	18,864	18,864	18,864	18,864	18,864

Table S46: Details of z statistics, p values and 99% CIs (in brackets) for the coefficient estimates reported in Table S45.

	PriorInteraction		Verified		Political leaning		Misinformation exposure	
	(1)	(2)	(3)	(4)	(5)	(6)	(7)	(8)
	Yes	No	Yes	No	Left	Right	High	Low
Display	z =0.84, p =0.403	z =7.33, $p < 0.001$	z =2.31, p =0.021	z =4.63, $p < 0.001$	z =3.98, $p < 0.001$	z =3.53, $p < 0.001$	z =4.20, $p < 0.001$	z =4.45, $p < 0.001$
	[−0.217, 0.426]	[0.581, 1.210]	[−0.033, 0.615]	[0.225, 0.788]	[0.178, 0.828]	[0.117, 0.745]	[0.187, 0.782]	[0.213, 0.801]
After	z =−1.55, p =0.122	z =2.04, p =0.041	z =−1.39, p =0.166	z =−0.35, p =0.724	z =−0.52, p =0.601	z =−0.72, p =0.474	z =−0.51, p =0.613	z =−0.16, p =0.873
	[−0.132, 0.033]	[−0.021, 0.187]	[−0.230, 0.069]	[−0.088, 0.067]	[−0.158, 0.104]	[−0.121, 0.069]	[−0.105, 0.071]	[−0.095, 0.084]
Display×After	z =−26.82, $p < 0.001$	z =−27.70, $p < 0.001$	z =−15.14, $p < 0.001$	z =−32.22, $p < 0.001$	z =−17.60, $p < 0.001$	z =−26.07, $p < 0.001$	z =−28.33, $p < 0.001$	z =−26.96, $p < 0.001$
	[−1.003, −0.827]	[−1.260, −1.045]	[−1.102, −0.782]	[−1.089, −0.928]	[−1.074, −0.800]	[−1.108, −0.909]	[−1.107, −0.923]	[−1.075, −0.888]
PostAge	z =−31.08, $p < 0.001$	z =−22.02, $p < 0.001$	z =−17.04, $p < 0.001$	z =−32.65, $p < 0.001$	z =−17.56, $p < 0.001$	z =−26.25, $p < 0.001$	z =−28.22, $p < 0.001$	z =−26.04, $p < 0.001$
	[−0.674, −0.571]	[−0.585, −0.462]	[−0.729, −0.537]	[−0.634, −0.541]	[−0.630, −0.469]	[−0.655, −0.538]	[−0.647, −0.539]	[−0.606, −0.497]
Intercept	z =−5.70, $p < 0.001$	z =−13.04, $p < 0.001$	z =−23.06, $p < 0.001$	z =0.73, p =0.463	z =−21.00, $p < 0.001$	z =−10.19, $p < 0.001$	z =−7.27, $p < 0.001$	z =−7.91, $p < 0.001$
	[−0.779, −0.294]	[−1.471, −0.986]	[−2.665, −2.129]	[−0.152, 0.273]	[−2.393, −1.870]	[−1.189, −0.709]	[−0.864, −0.412]	[−0.910, −0.463]
Post-level RE	✓	✓	✓	✓	✓	✓	✓	✓
#Observations	18,864	18,864	18,864	18,864	18,864	18,864	18,864	18,864

Table S47: Regression results for two-period ATT estimations, based on posts from low-misinformation-exposure posters and across reposter characteristics. Post-specific random effects are included. Reported are coefficient estimates with standard errors in parentheses. * $p < 0.01$, ** $p < 0.005$, *** $p < 0.001$. Exact z statistics, p values and 99% CIs are reported in Table S48.

	PriorInteraction		Verified		Political leaning		Misinformation exposure	
	(1)	(2)	(3)	(4)	(5)	(6)	(7)	(8)
	Yes	No	Yes	No	Left	Right	High	Low
Display	0.334	0.791***	0.374	0.599***	0.295	1.156***	0.828***	0.501***
	(0.166)	(0.161)	(0.183)	(0.145)	(0.160)	(0.173)	(0.155)	(0.150)
After	0.022	0.040	−0.075	0.025	−0.008	0.096	0.057	0.019
	(0.050)	(0.052)	(0.117)	(0.043)	(0.052)	(0.084)	(0.061)	(0.046)
Display × After	−0.815***	−0.957***	−0.694***	−0.892***	−0.851***	−0.869***	−0.851***	−0.895***
	(0.054)	(0.056)	(0.133)	(0.046)	(0.057)	(0.089)	(0.064)	(0.049)
PostAge	−0.737***	−0.552***	−0.630***	−0.634***	−0.623***	−0.657***	−0.629***	−0.618***
	(0.033)	(0.032)	(0.073)	(0.027)	(0.034)	(0.052)	(0.038)	(0.029)
Intercept	−1.465***	−1.270***	−3.592***	−0.413***	−1.233***	−3.113***	−1.954***	−0.678***
	(0.122)	(0.120)	(0.168)	(0.106)	(0.118)	(0.147)	(0.120)	(0.110)
Post-level RE	✓	✓	✓	✓	✓	✓	✓	✓
#Observations	10,448	10,448	10,448	10,448	10,448	10,448	10,448	10,448

Table S48: Details of z statistics, p values and 99% CIs (in brackets) for the coefficient estimates reported in Table S47.

	PriorInteraction		Verified		Political leaning		Misinformation exposure	
	(1)	(2)	(3)	(4)	(5)	(6)	(7)	(8)
	Yes	No	Yes	No	Left	Right	High	Low
Display	z =2.02, p =0.044	z =4.90, $p < 0.001$	z =2.04, p =0.041	z =4.13, $p < 0.001$	z =1.84, p =0.065	z =6.67, $p < 0.001$	z =5.33, $p < 0.001$	z =3.34, $p < 0.001$
	[−0.092, 0.761]	[0.376, 1.207]	[−0.097, 0.844]	[0.225, 0.973]	[−0.117, 0.707]	[0.709, 1.603]	[0.428, 1.228]	[0.115, 0.888]
After	z =0.45, p =0.655	z =0.76, p =0.447	z =−0.64, p =0.522	z =0.59, p =0.553	z =−0.15, p =0.877	z =1.14, p =0.254	z =0.94, p =0.349	z =0.42, p =0.675
	[−0.106, 0.151]	[−0.095, 0.175]	[−0.375, 0.226]	[−0.085, 0.136]	[−0.143, 0.126]	[−0.120, 0.312]	[−0.099, 0.213]	[−0.098, 0.137]
Display×After	z =−15.17, $p < 0.001$	z =−17.23, $p < 0.001$	z =−5.22, $p < 0.001$	z =−19.53, $p < 0.001$	z =−14.96, $p < 0.001$	z =−9.79, $p < 0.001$	z =−13.30, $p < 0.001$	z =−18.36, $p < 0.001$
	[−0.953, −0.676]	[−1.101, −0.814]	[−1.036, −0.352]	[−1.010, −0.774]	[−0.997, −0.704]	[−1.097, −0.640]	[−1.016, −0.686]	[−1.021, −0.770]
PostAge	z =−22.36, $p < 0.001$	z =−17.04, $p < 0.001$	z =−8.61, $p < 0.001$	z =−23.73, $p < 0.001$	z =−18.53, $p < 0.001$	z =−12.60, $p < 0.001$	z =−16.40, $p < 0.001$	z =−21.57, $p < 0.001$
	[−0.822, −0.652]	[−0.635, −0.468]	[−0.819, −0.442]	[−0.703, −0.565]	[−0.709, −0.536]	[−0.791, −0.523]	[−0.728, −0.530]	[−0.692, −0.544]
Intercept	z =−12.01, $p < 0.001$	z =−10.61, $p < 0.001$	z =−21.36, $p < 0.001$	z =−3.90, $p < 0.001$	z =−10.42, $p < 0.001$	z =−21.24, $p < 0.001$	z =−16.28, $p < 0.001$	z =−6.18, $p < 0.001$
	[−1.779, −1.151]	[−1.579, −0.962]	[−4.025, −3.159]	[−0.685, −0.140]	[−1.538, −0.929]	[−3.491, −2.736]	[−2.263, −1.645]	[−0.960, −0.395]
Post-level RE	✓	✓	✓	✓	✓	✓	✓	✓
#Observations	10,448	10,448	10,448	10,448	10,448	10,448	10,448	10,448

Table S49: Regression results for two-period ATT estimations, based on posts from verified posters and across reposter characteristics. Post-specific random effects are included. Reported are coefficient estimates with standard errors in parentheses. * $p < 0.01$, ** $p < 0.005$, *** $p < 0.001$. Exact z statistics, p values and 99% CIs are reported in Table S50.

	PriorInteraction		Verified		Political leaning		Misinformation exposure	
	(1)	(2)	(3)	(4)	(5)	(6)	(7)	(8)
	Yes	No	Yes	No	Left	Right	High	Low
Display	0.302***	0.762***	0.367***	0.533***	0.387***	0.626***	0.576***	0.501***
	(0.082)	(0.083)	(0.086)	(0.074)	(0.086)	(0.086)	(0.080)	(0.076)
After	−0.050	0.071*	−0.133**	−0.006	−0.036	−0.016	−0.015	−0.014
	(0.022)	(0.027)	(0.043)	(0.020)	(0.031)	(0.028)	(0.025)	(0.022)
Display × After	−0.863***	−1.083***	−0.876***	−0.952***	−0.884***	−0.967***	−0.973***	−0.920***
	(0.023)	(0.028)	(0.046)	(0.021)	(0.032)	(0.029)	(0.026)	(0.023)
PostAge	−0.628***	−0.503***	−0.571***	−0.588***	−0.558***	−0.579***	−0.579***	−0.555***
	(0.013)	(0.016)	(0.027)	(0.012)	(0.019)	(0.017)	(0.015)	(0.014)
Intercept	−0.608***	−1.254***	−2.661***	−0.032	−1.911***	−1.479***	−0.982***	−0.592***
	(0.061)	(0.064)	(0.072)	(0.055)	(0.067)	(0.066)	(0.060)	(0.057)
Post-level RE	✓	✓	✓	✓	✓	✓	✓	✓
#Observations	41,040	41,040	41,040	41,040	41,040	41,040	41,040	41,040

Table S50: Details of z statistics, p values and 99% CIs (in brackets) for the coefficient estimates reported in Table S49.

	PriorInteraction		Verified		Political leaning		Misinformation exposure	
	(1)	(2)	(3)	(4)	(5)	(6)	(7)	(8)
	Yes	No	Yes	No	Left	Right	High	Low
Display	z =3.71, $p < 0.001$	z =9.16, $p < 0.001$	z =4.26, $p < 0.001$	z =7.19, $p < 0.001$	z =4.53, $p < 0.001$	z =7.32, $p < 0.001$	z =7.24, $p < 0.001$	z =6.56, $p < 0.001$
	[0.092, 0.513]	[0.548, 0.976]	[0.145, 0.588]	[0.342, 0.724]	[0.167, 0.608]	[0.406, 0.846]	[0.371, 0.781]	[0.304, 0.697]
After	z =−2.32, p =0.020	z =2.62, p =0.009	z =−3.09, p =0.002	z =−0.30, p =0.762	z =−1.17, p =0.243	z =−0.60, p =0.549	z =−0.63, p =0.529	z =−0.61, p =0.540
	[−0.105, 0.005]	[0.001, 0.140]	[−0.244, −0.022]	[−0.057, 0.045]	[−0.115, 0.043]	[−0.087, 0.054]	[−0.079, 0.048]	[−0.071, 0.044]
Display×After	z =−38.10, $p < 0.001$	z =−38.57, $p < 0.001$	z =−18.88, $p < 0.001$	z =−45.53, $p < 0.001$	z =−27.24, $p < 0.001$	z =−33.64, $p < 0.001$	z =−37.83, $p < 0.001$	z =−39.15, $p < 0.001$
	[−0.922, −0.805]	[−1.155, −1.010]	[−0.996, −0.756]	[−1.006, −0.898]	[−0.968, −0.801]	[−1.041, −0.893]	[−1.039, −0.907]	[−0.980, −0.859]
PostAge	z =−46.63, $p < 0.001$	z =−31.31, $p < 0.001$	z =−21.08, $p < 0.001$	z =−48.71, $p < 0.001$	z =−28.85, $p < 0.001$	z =−34.21, $p < 0.001$	z =−38.22, $p < 0.001$	z =−40.49, $p < 0.001$
	[−0.663, −0.593]	[−0.545, −0.462]	[−0.640, −0.501]	[−0.619, −0.557]	[−0.608, −0.509]	[−0.622, −0.535]	[−0.618, −0.540]	[−0.590, −0.520]
Intercept	z =−9.95, $p < 0.001$	z =−19.74, $p < 0.001$	z =−36.75, $p < 0.001$	z =−0.57, p =0.568	z =−28.47, $p < 0.001$	z =−22.50, $p < 0.001$	z =−16.23, $p < 0.001$	z =−10.31, $p < 0.001$
	[−0.766, −0.451]	[−1.417, −1.090]	[−2.848, −2.475]	[−0.174, 0.111]	[−2.084, −1.738]	[−1.648, −1.310]	[−1.137, −0.826]	[−0.740, −0.444]
Post-level RE	✓	✓	✓	✓	✓	✓	✓	✓
#Observations	41,040	41,040	41,040	41,040	41,040	41,040	41,040	41,040

Table S51: Regression results for two-period ATT estimations, based on posts from non-verified posters and across reposter characteristics. Post-specific random effects are included. Reported are coefficient estimates with standard errors in parentheses. * $p < 0.01$, ** $p < 0.005$, *** $p < 0.001$. Exact z statistics, p values and 99% CIs are reported in Table S52.

	PriorInteraction		Verified		Political leaning		Misinformation exposure	
	(1)	(2)	(3)	(4)	(5)	(6)	(7)	(8)
	Yes	No	Yes	No	Left	Right	High	Low
Display	0.350	1.289***	1.116***	1.066***	0.871***	1.269***	1.177***	1.037***
	(0.193)	(0.175)	(0.253)	(0.157)	(0.189)	(0.191)	(0.177)	(0.165)
After	−0.018	0.061	0.144	0.013	0.055	0.065	0.035	0.026
	(0.080)	(0.065)	(0.188)	(0.058)	(0.077)	(0.106)	(0.081)	(0.062)
Display × After	−1.129***	−1.390***	−1.366***	−1.304***	−1.379***	−1.201***	−1.238***	−1.336***
	(0.090)	(0.068)	(0.205)	(0.062)	(0.082)	(0.111)	(0.085)	(0.065)
PostAge	−0.620***	−0.500***	−0.412***	−0.535***	−0.563***	−0.493***	−0.512***	−0.507***
	(0.052)	(0.038)	(0.100)	(0.035)	(0.047)	(0.061)	(0.048)	(0.037)
Intercept	−2.625***	−1.353***	−4.734***	−0.782***	−2.185***	−3.310***	−2.378***	−1.120***
	(0.154)	(0.132)	(0.257)	(0.118)	(0.147)	(0.166)	(0.142)	(0.124)
Post-level RE	✓	✓	✓	✓	✓	✓	✓	✓
#Observations	9,568	9,568	9,568	9,568	9,568	9,568	9,568	9,568

Table S52: Details of z statistics, p values and 99% CIs (in brackets) for the coefficient estimates reported in Table S51.

	PriorInteraction		Verified		Political leaning		Misinformation exposure	
	(1)	(2)	(3)	(4)	(5)	(6)	(7)	(8)
	Yes	No	Yes	No	Left	Right	High	Low
Display	z =1.81, p =0.070	z =7.35, $p < 0.001$	z =4.41, $p < 0.001$	z =6.78, $p < 0.001$	z =4.61, $p < 0.001$	z =6.65, $p < 0.001$	z =6.67, $p < 0.001$	z =6.29, $p < 0.001$
	[−0.148, 0.847]	[0.837, 1.741]	[0.465, 1.768]	[0.661, 1.470]	[0.385, 1.358]	[0.778, 1.761]	[0.722, 1.632]	[0.612, 1.461]
After	z =−0.23, p =0.822	z =0.93, p =0.350	z =0.76, p =0.445	z =0.22, p =0.825	z =0.72, p =0.472	z =0.61, p =0.539	z =0.43, p =0.665	z =0.42, p =0.672
	[−0.224, 0.188]	[−0.107, 0.229]	[−0.340, 0.627]	[−0.137, 0.163]	[−0.143, 0.253]	[−0.208, 0.338]	[−0.173, 0.243]	[−0.133, 0.185]
Display×After	z =−12.59, $p < 0.001$	z =−20.31, $p < 0.001$	z =−6.67, $p < 0.001$	z =−21.13, $p < 0.001$	z =−16.84, $p < 0.001$	z =−10.86, $p < 0.001$	z =−14.64, $p < 0.001$	z =−20.43, $p < 0.001$
	[−1.360, −0.898]	[−1.567, −1.214]	[−1.893, −0.838]	[−1.463, −1.145]	[−1.590, −1.168]	[−1.486, −0.916]	[−1.456, −1.020]	[−1.505, −1.168]
PostAge	z =−11.91, $p < 0.001$	z =−13.12, $p < 0.001$	z =−4.11, $p < 0.001$	z =−15.47, $p < 0.001$	z =−12.01, $p < 0.001$	z =−8.05, $p < 0.001$	z =−10.65, $p < 0.001$	z =−13.80, $p < 0.001$
	[−0.754, −0.486]	[−0.598, −0.402]	[−0.670, −0.154]	[−0.624, −0.446]	[−0.684, −0.442]	[−0.651, −0.335]	[−0.636, −0.388]	[−0.602, −0.413]
Intercept	z =−17.07, $p < 0.001$	z =−10.22, $p < 0.001$	z =−18.45, $p < 0.001$	z =−6.64, $p < 0.001$	z =−14.88, $p < 0.001$	z =−19.92, $p < 0.001$	z =−16.80, $p < 0.001$	z =−9.03, $p < 0.001$
	[−3.021, −2.229]	[−1.695, −1.012]	[−5.395, −4.073]	[−1.086, −0.479]	[−2.563, −1.806]	[−3.738, −2.882]	[−2.743, −2.014]	[−1.440, −0.801]
Post-level RE	✓	✓	✓	✓	✓	✓	✓	✓
#Observations	9,568	9,568	9,568	9,568	9,568	9,568	9,568	9,568

Table S53: Regression results for two-period ATT estimations, based on posts from older accounts and across reposter characteristics. Post-specific random effects are included. Reported are coefficient estimates with standard errors in parentheses. * $p < 0.01$, ** $p < 0.005$, *** $p < 0.001$. Exact z statistics, p values and 99% CIs are reported in Table S54.

	PriorInteraction		Verified		Political leaning		Misinformation exposure	
	(1)	(2)	(3)	(4)	(5)	(6)	(7)	(8)
	Yes	No	Yes	No	Left	Right	High	Low
Display	0.372***	0.739***	0.448***	0.590***	0.313**	0.816***	0.704***	0.531***
	(0.106)	(0.107)	(0.113)	(0.093)	(0.109)	(0.111)	(0.102)	(0.096)
After	−0.020	0.086	−0.113	0.016	−0.032	0.025	0.018	0.001
	(0.030)	(0.036)	(0.060)	(0.027)	(0.041)	(0.039)	(0.034)	(0.031)
Display × After	−0.907***	−1.125***	−0.944***	−0.993***	−0.913***	−1.022***	−1.005***	−0.979***
	(0.031)	(0.037)	(0.064)	(0.028)	(0.044)	(0.040)	(0.035)	(0.032)
PostAge	−0.667***	−0.527***	−0.632***	−0.603***	−0.558***	−0.642***	−0.622***	−0.558***
	(0.019)	(0.022)	(0.038)	(0.017)	(0.026)	(0.024)	(0.021)	(0.019)
Intercept	−1.032***	−1.355***	−3.022***	−0.241***	−1.860***	−1.977***	−1.333***	−0.747***
	(0.080)	(0.082)	(0.099)	(0.070)	(0.086)	(0.087)	(0.079)	(0.073)
Post-level RE	✓	✓	✓	✓	✓	✓	✓	✓
#Observations	25,296	25,296	25,296	25,296	25,296	25,296	25,296	25,296

Table S54: Details of z statistics, p values and 99% CIs (in brackets) for the coefficient estimates reported in Table S53.

	PriorInteraction		Verified		Political leaning		Misinformation exposure	
	(1)	(2)	(3)	(4)	(5)	(6)	(7)	(8)
	Yes	No	Yes	No	Left	Right	High	Low
Display	z =3.52, p < 0.001	z =6.91, p < 0.001	z =3.95, p < 0.001	z =6.37, p < 0.001	z =2.86, p =0.004	z =7.36, p < 0.001	z =6.92, p < 0.001	z =5.52, p < 0.001
	[0.099, 0.644]	[0.463, 1.014]	[0.156, 0.741]	[0.351, 0.828]	[0.031, 0.594]	[0.530, 1.101]	[0.442, 0.966]	[0.283, 0.778]
After	z =−0.65, p =0.513	z =2.39, p =0.017	z =−1.90, p =0.058	z =0.59, p =0.556	z =−0.79, p =0.431	z =0.65, p =0.515	z =0.52, p =0.602	z =0.04, p =0.968
	[−0.097, 0.058]	[−0.007, 0.179]	[−0.267, 0.040]	[−0.054, 0.086]	[−0.139, 0.074]	[−0.074, 0.124]	[−0.070, 0.105]	[−0.078, 0.080]
Display×After	z =−29.00, p < 0.001	z =−30.13, p < 0.001	z =−14.79, p < 0.001	z =−34.99, p < 0.001	z =−20.91, p < 0.001	z =−25.67, p < 0.001	z =−28.57, p < 0.001	z =−30.61, p < 0.001
	[−0.988, −0.827]	[−1.221, −1.029]	[−1.109, −0.780]	[−1.066, −0.920]	[−1.026, −0.801]	[−1.125, −0.920]	[−1.096, −0.914]	[−1.061, −0.896]
PostAge	z =−35.14, p < 0.001	z =−24.37, p < 0.001	z =−16.79, p < 0.001	z =−36.48, p < 0.001	z =−21.58, p < 0.001	z =−26.90, p < 0.001	z =−29.54, p < 0.001	z =−29.69, p < 0.001
	[−0.716, −0.618]	[−0.583, −0.472]	[−0.729, −0.535]	[−0.646, −0.561]	[−0.625, −0.492]	[−0.703, −0.580]	[−0.676, −0.567]	[−0.607, −0.510]
Intercept	z =−12.88, p < 0.001	z =−16.46, p < 0.001	z =−30.66, p < 0.001	z =−3.44, p < 0.001	z =−21.63, p < 0.001	z =−22.64, p < 0.001	z =−16.96, p < 0.001	z =−10.22, p < 0.001
	[−1.238, −0.826]	[−1.567, −1.143]	[−3.276, −2.768]	[−0.421, −0.061]	[−2.081, −1.638]	[−2.202, −1.752]	[−1.536, −1.131]	[−0.935, −0.558]
Post-level RE	✓	✓	✓	✓	✓	✓	✓	✓
#Observations	25,296	25,296	25,296	25,296	25,296	25,296	25,296	25,296

Table S55: Regression results for two-period ATT estimations, based on posts from younger accounts and across reposter characteristics. Post-specific random effects are included. Reported are coefficient estimates with standard errors in parentheses. * $p < 0.01$, ** $p < 0.005$, *** $p < 0.001$. Exact z statistics, p values and 99% CIs are reported in Table S56.

	PriorInteraction		Verified		Political leaning		Misinformation exposure	
	(1)	(2)	(3)	(4)	(5)	(6)	(7)	(8)
	Yes	No	Yes	No	Left	Right	High	Low
Display	0.293*	0.992***	0.464***	0.685***	0.655***	0.664***	0.668***	0.681***
	(0.114)	(0.106)	(0.118)	(0.098)	(0.111)	(0.114)	(0.106)	(0.100)
After	−0.064	0.059	−0.109	−0.017	0.000	−0.030	−0.025	−0.012
	(0.029)	(0.035)	(0.059)	(0.027)	(0.040)	(0.037)	(0.033)	(0.030)
Display × After	−0.852***	−1.164***	−0.858***	−1.018***	−1.046***	−0.942***	−0.996***	−1.000***
	(0.031)	(0.037)	(0.064)	(0.028)	(0.043)	(0.039)	(0.034)	(0.031)
PostAge	−0.600***	−0.481***	−0.516***	−0.563***	−0.556***	−0.528***	−0.538***	−0.538***
	(0.018)	(0.021)	(0.037)	(0.016)	(0.025)	(0.022)	(0.020)	(0.018)
Intercept	−0.949***	−1.199***	−3.005***	−0.122	−2.068***	−1.703***	−1.173***	−0.646***
	(0.084)	(0.080)	(0.101)	(0.072)	(0.087)	(0.087)	(0.080)	(0.074)
Post-level RE	✓	✓	✓	✓	✓	✓	✓	✓
#Observations	25,312	25,312	25,312	25,312	25,312	25,312	25,312	25,312

Table S56: Details of z statistics, p values and 99% CIs (in brackets) for the coefficient estimates reported in Table S55.

	PriorInteraction		Verified		Political leaning		Misinformation exposure	
	(1)	(2)	(3)	(4)	(5)	(6)	(7)	(8)
	Yes	No	Yes	No	Left	Right	High	Low
Display	z =2.58, p =0.010	z =9.41, p < 0.001	z =3.92, p < 0.001	z =6.99, p < 0.001	z =5.87, p < 0.001	z =5.84, p < 0.001	z =6.29, p < 0.001	z =6.83, p < 0.001
	[0.001, 0.586]	[0.721, 1.264]	[0.159, 0.769]	[0.433, 0.937]	[0.367, 0.942]	[0.371, 0.957]	[0.394, 0.941]	[0.424, 0.938]
After	z =−2.23, p =0.026	z =1.68, p =0.094	z =−1.86, p =0.063	z =−0.62, p =0.534	z =0.01, p =0.991	z =−0.83, p =0.407	z =−0.77, p =0.443	z =−0.42, p =0.675
	[−0.138, 0.010]	[−0.032, 0.149]	[−0.260, 0.042]	[−0.085, 0.052]	[−0.103, 0.104]	[−0.125, 0.064]	[−0.109, 0.059]	[−0.088, 0.064]
Display×After	z =−27.66, p < 0.001	z =−31.68, p < 0.001	z =−13.48, p < 0.001	z =−36.07, p < 0.001	z =−24.55, p < 0.001	z =−24.36, p < 0.001	z =−28.91, p < 0.001	z =−31.96, p < 0.001
	[−0.931, −0.772]	[−1.259, −1.069]	[−1.022, −0.694]	[−1.091, −0.946]	[−1.156, −0.937]	[−1.042, −0.842]	[−1.084, −0.907]	[−1.081, −0.919]
PostAge	z =−33.33, p < 0.001	z =−23.41, p < 0.001	z =−14.11, p < 0.001	z =−35.17, p < 0.001	z =−22.16, p < 0.001	z =−23.53, p < 0.001	z =−26.90, p < 0.001	z =−30.09, p < 0.001
	[−0.646, −0.554]	[−0.534, −0.428]	[−0.611, −0.422]	[−0.604, −0.521]	[−0.621, −0.491]	[−0.586, −0.470]	[−0.589, −0.486]	[−0.584, −0.492]
Intercept	z =−11.27, p < 0.001	z =−15.05, p < 0.001	z =−29.70, p < 0.001	z =−1.69, p =0.091	z =−23.81, p < 0.001	z =−19.64, p < 0.001	z =−14.70, p < 0.001	z =−8.69, p < 0.001
	[−1.165, −0.732]	[−1.404, −0.994]	[−3.266, −2.744]	[−0.309, 0.064]	[−2.292, −1.844]	[−1.926, −1.480]	[−1.378, −0.967]	[−0.837, −0.454]
Post-level RE	✓	✓	✓	✓	✓	✓	✓	✓
#Observations	25,312	25,312	25,312	25,312	25,312	25,312	25,312	25,312

Table S57: Regression results for two-period ATT estimations, based on posts from accounts with higher follower counts and across reposter characteristics. Post-specific random effects are included. Reported are coefficient estimates with standard errors in parentheses. * $p < 0.01$, ** $p < 0.005$, *** $p < 0.001$. Exact z statistics, p values and 99% CIs are reported in Table S58.

	PriorInteraction		Verified		Political leaning		Misinformation exposure	
	(1)	(2)	(3)	(4)	(5)	(6)	(7)	(8)
	Yes	No	Yes	No	Left	Right	High	Low
Display	0.218	0.504***	0.243	0.328***	0.120	0.474***	0.409***	0.280**
	(0.096)	(0.108)	(0.102)	(0.093)	(0.104)	(0.106)	(0.098)	(0.096)
After	−0.047	0.077	−0.057	−0.012	−0.024	−0.010	−0.006	−0.020
	(0.024)	(0.031)	(0.048)	(0.023)	(0.034)	(0.031)	(0.028)	(0.025)
Display × After	−0.821***	−0.970***	−0.840***	−0.863***	−0.813***	−0.873***	−0.887***	−0.837***
	(0.025)	(0.033)	(0.052)	(0.024)	(0.036)	(0.033)	(0.029)	(0.026)
PostAge	−0.635***	−0.507***	−0.662***	−0.598***	−0.555***	−0.634***	−0.620***	−0.553***
	(0.015)	(0.019)	(0.031)	(0.014)	(0.021)	(0.019)	(0.017)	(0.015)
Intercept	0.044	−1.046***	−2.280***	0.425***	−1.361***	−1.068***	−0.541***	−0.131
	(0.072)	(0.082)	(0.084)	(0.070)	(0.080)	(0.081)	(0.074)	(0.072)
Post-level RE	✓	✓	✓	✓	✓	✓	✓	✓
#Observations	25,296	25,296	25,296	25,296	25,296	25,296	25,296	25,296

Table S58: Details of z statistics, p values and 99% CIs (in brackets) for the coefficient estimates reported in Table S57.

	PriorInteraction		Verified		Political leaning		Misinformation exposure	
	(1)	(2)	(3)	(4)	(5)	(6)	(7)	(8)
	Yes	No	Yes	No	Left	Right	High	Low
Display	z =2.27, p =0.023	z =4.67, $p < 0.001$	z =2.38, p =0.017	z =3.53, $p < 0.001$	z =1.15, p =0.249	z =4.47, $p < 0.001$	z =4.19, $p < 0.001$	z =2.92, p =0.003
	[−0.029, 0.465]	[0.226, 0.782]	[−0.020, 0.507]	[0.088, 0.567]	[−0.149, 0.389]	[0.201, 0.747]	[0.157, 0.660]	[0.033, 0.528]
After	z =−1.98, p =0.048	z =2.46, p =0.014	z =−1.18, p =0.239	z =−0.52, p =0.606	z =−0.70, p =0.485	z =−0.33, p =0.741	z =−0.23, p =0.816	z =−0.78, p =0.437
	[−0.109, 0.014]	[−0.004, 0.158]	[−0.181, 0.068]	[−0.070, 0.047]	[−0.111, 0.064]	[−0.091, 0.070]	[−0.078, 0.065]	[−0.084, 0.045]
Display×After	z =−32.70, $p < 0.001$	z =−29.66, $p < 0.001$	z =−16.19, $p < 0.001$	z =−36.36, $p < 0.001$	z =−22.59, $p < 0.001$	z =−26.77, $p < 0.001$	z =−30.50, $p < 0.001$	z =−31.77, $p < 0.001$
	[−0.886, −0.756]	[−1.054, −0.886]	[−0.974, −0.707]	[−0.924, −0.802]	[−0.906, −0.720]	[−0.957, −0.789]	[−0.962, −0.812]	[−0.905, −0.769]
PostAge	z =−42.79, $p < 0.001$	z =−26.90, $p < 0.001$	z =−21.39, $p < 0.001$	z =−43.67, $p < 0.001$	z =−25.84, $p < 0.001$	z =−32.70, $p < 0.001$	z =−35.97, $p < 0.001$	z =−35.95, $p < 0.001$
	[−0.674, −0.597]	[−0.555, −0.458]	[−0.742, −0.582]	[−0.633, −0.562]	[−0.610, −0.499]	[−0.684, −0.584]	[−0.665, −0.576]	[−0.593, −0.513]
Intercept	z =0.61, p =0.542	z =−12.69, $p < 0.001$	z =−27.02, $p < 0.001$	z =6.09, $p < 0.001$	z =−16.92, $p < 0.001$	z =−13.14, $p < 0.001$	z =−7.29, $p < 0.001$	z =−1.81, p =0.070
	[−0.142, 0.230]	[−1.258, −0.833]	[−2.497, −2.062]	[0.245, 0.605]	[−1.568, −1.154]	[−1.278, −0.859]	[−0.732, −0.350]	[−0.317, 0.055]
Post-level RE	✓	✓	✓	✓	✓	✓	✓	✓
#Observations	25,296	25,296	25,296	25,296	25,296	25,296	25,296	25,296

Table S59: Regression results for two-period ATT estimations, based on posts from accounts with lower follower counts and across reposter characteristics. Post-specific random effects are included. Reported are coefficient estimates with standard errors in parentheses. * $p < 0.01$, ** $p < 0.005$, *** $p < 0.001$. Exact z statistics, p values and 99% CIs are reported in Table S60.

	PriorInteraction		Verified		Political leaning		Misinformation exposure	
	(1)	(2)	(3)	(4)	(5)	(6)	(7)	(8)
	Yes	No	Yes	No	Left	Right	High	Low
Display	0.347**	1.209***	0.644***	0.917***	0.855***	0.977***	0.933***	0.913***
	(0.108)	(0.104)	(0.130)	(0.092)	(0.115)	(0.113)	(0.104)	(0.096)
After	−0.066	0.061	−0.269**	0.002	−0.031	−0.006	−0.024	0.005
	(0.041)	(0.040)	(0.083)	(0.033)	(0.051)	(0.050)	(0.043)	(0.038)
Display × After	−1.061***	−1.337***	−1.071***	−1.229***	−1.231***	−1.225***	−1.222***	−1.225***
	(0.045)	(0.042)	(0.091)	(0.035)	(0.054)	(0.052)	(0.045)	(0.040)
PostAge	−0.591***	−0.497***	−0.320***	−0.544***	−0.544***	−0.447***	−0.476***	−0.527***
	(0.027)	(0.024)	(0.048)	(0.020)	(0.032)	(0.030)	(0.026)	(0.023)
Intercept	−1.936***	−1.484***	−3.636***	−0.734***	−2.541***	−2.517***	−1.888***	−1.217***
	(0.084)	(0.080)	(0.119)	(0.069)	(0.093)	(0.091)	(0.081)	(0.073)
Post-level RE	✓	✓	✓	✓	✓	✓	✓	✓
#Observations	25,312	25,312	25,312	25,312	25,312	25,312	25,312	25,312

Table S60: Details of z statistics, p values and 99% CIs (in brackets) for the coefficient estimates reported in Table S59.

	PriorInteraction		Verified		Political leaning		Misinformation exposure	
	(1)	(2)	(3)	(4)	(5)	(6)	(7)	(8)
	Yes	No	Yes	No	Left	Right	High	Low
Display	z =3.20, p =0.001	z =11.60, p < 0.001	z =4.97, p < 0.001	z =9.94, p < 0.001	z =7.44, p < 0.001	z =8.67, p < 0.001	z =8.97, p < 0.001	z =9.51, p < 0.001
	[0.068, 0.626]	[0.941, 1.478]	[0.310, 0.978]	[0.679, 1.154]	[0.559, 1.151]	[0.687, 1.268]	[0.665, 1.201]	[0.666, 1.161]
After	z =−1.60, p =0.109	z =1.53, p =0.125	z =−3.25, p =0.001	z =0.05, p =0.961	z =−0.60, p =0.547	z =−0.13, p =0.899	z =−0.56, p =0.576	z =0.12, p =0.904
	[−0.173, 0.040]	[−0.042, 0.164]	[−0.482, −0.056]	[−0.085, 0.088]	[−0.163, 0.101]	[−0.135, 0.122]	[−0.135, 0.087]	[−0.092, 0.101]
Display×After	z =−23.49, p < 0.001	z =−32.03, p < 0.001	z =−11.79, p < 0.001	z =−34.75, p < 0.001	z =−22.60, p < 0.001	z =−23.40, p < 0.001	z =−26.94, p < 0.001	z =−30.85, p < 0.001
	[−1.177, −0.944]	[−1.445, −1.230]	[−1.305, −0.837]	[−1.321, −1.138]	[−1.371, −1.090]	[−1.360, −1.090]	[−1.339, −1.105]	[−1.327, −1.123]
PostAge	z =−22.16, p < 0.001	z =−21.14, p < 0.001	z =−6.67, p < 0.001	z =−26.98, p < 0.001	z =−17.18, p < 0.001	z =−15.07, p < 0.001	z =−18.29, p < 0.001	z =−23.24, p < 0.001
	[−0.659, −0.522]	[−0.557, −0.436]	[−0.443, −0.196]	[−0.596, −0.492]	[−0.625, −0.462]	[−0.524, −0.371]	[−0.543, −0.409]	[−0.586, −0.469]
Intercept	z =−23.08, p < 0.001	z =−18.62, p < 0.001	z =−30.48, p < 0.001	z =−10.57, p < 0.001	z =−27.32, p < 0.001	z =−27.65, p < 0.001	z =−23.21, p < 0.001	z =−16.61, p < 0.001
	[−2.152, −1.720]	[−1.690, −1.279]	[−3.943, −3.329]	[−0.913, −0.555]	[−2.781, −2.301]	[−2.751, −2.282]	[−2.098, −1.679]	[−1.406, −1.029]
Post-level RE	✓	✓	✓	✓	✓	✓	✓	✓
#Observations	25,312	25,312	25,312	25,312	25,312	25,312	25,312	25,312

Table S61: Regression results for two-period ATT estimations, based on posts from accounts with higher followee counts and across reposter characteristics. Post-specific random effects are included. Reported are coefficient estimates with standard errors in parentheses. * $p < 0.01$, ** $p < 0.005$, *** $p < 0.001$. Exact z statistics, p values and 99% CIs are reported in Table S62.

	PriorInteraction		Verified		Political leaning		Misinformation exposure	
	(1)	(2)	(3)	(4)	(5)	(6)	(7)	(8)
	Yes	No	Yes	No	Left	Right	High	Low
Display	0.380***	0.792***	0.489***	0.632***	0.397***	0.804***	0.744***	0.584***
	(0.103)	(0.106)	(0.112)	(0.092)	(0.110)	(0.111)	(0.101)	(0.095)
After	−0.047	−0.002	−0.112	−0.038	−0.081	−0.046	−0.050	−0.032
	(0.030)	(0.037)	(0.060)	(0.028)	(0.043)	(0.038)	(0.034)	(0.031)
Display × After	−0.951***	−1.128***	−1.024***	−1.026***	−0.946***	−1.059***	−1.050***	−1.008***
	(0.032)	(0.038)	(0.065)	(0.029)	(0.045)	(0.039)	(0.035)	(0.032)
PostAge	−0.643***	−0.498***	−0.585***	−0.588***	−0.555***	−0.578***	−0.581***	−0.547***
	(0.019)	(0.022)	(0.037)	(0.017)	(0.027)	(0.023)	(0.021)	(0.019)
Intercept	−0.871***	−1.215***	−2.859***	−0.144	−1.921***	−1.716***	−1.149***	−0.709***
	(0.078)	(0.082)	(0.097)	(0.070)	(0.088)	(0.087)	(0.079)	(0.073)
Post-level RE	✓	✓	✓	✓	✓	✓	✓	✓
#Observations	25,296	25,296	25,296	25,296	25,296	25,296	25,296	25,296

Table S62: Details of z statistics, p values and 99% CIs (in brackets) for the coefficient estimates reported in Table S61.

	PriorInteraction		Verified		Political leaning		Misinformation exposure	
	(1)	(2)	(3)	(4)	(5)	(6)	(7)	(8)
	Yes	No	Yes	No	Left	Right	High	Low
Display	z =3.71, $p < 0.001$	z =7.50, $p < 0.001$	z =4.35, $p < 0.001$	z =6.83, $p < 0.001$	z =3.60, $p < 0.001$	z =7.25, $p < 0.001$	z =7.33, $p < 0.001$	z =6.14, $p < 0.001$
	[0.116, 0.645]	[0.520, 1.064]	[0.199, 0.778]	[0.394, 0.870]	[0.113, 0.681]	[0.519, 1.090]	[0.483, 1.006]	[0.339, 0.829]
After	z =−1.56, p =0.118	z =−0.06, p =0.953	z =−1.86, p =0.063	z =−1.36, p =0.173	z =−1.87, p =0.061	z =−1.21, p =0.226	z =−1.47, p =0.141	z =−1.01, p =0.313
	[−0.125, 0.031]	[−0.097, 0.092]	[−0.268, 0.043]	[−0.109, 0.033]	[−0.192, 0.030]	[−0.145, 0.052]	[−0.137, 0.038]	[−0.112, 0.049]
Display×After	z =−30.04, $p < 0.001$	z =−29.86, $p < 0.001$	z =−15.81, $p < 0.001$	z =−35.86, $p < 0.001$	z =−20.95, $p < 0.001$	z =−26.88, $p < 0.001$	z =−29.98, $p < 0.001$	z =−31.02, $p < 0.001$
	[−1.033, −0.870]	[−1.225, −1.030]	[−1.191, −0.858]	[−1.100, −0.952]	[−1.062, −0.830]	[−1.160, −0.957]	[−1.140, −0.960]	[−1.092, −0.924]
PostAge	z =−33.96, $p < 0.001$	z =−22.96, $p < 0.001$	z =−15.68, $p < 0.001$	z =−35.51, $p < 0.001$	z =−20.75, $p < 0.001$	z =−24.83, $p < 0.001$	z =−28.04, $p < 0.001$	z =−28.90, $p < 0.001$
	[−0.692, −0.595]	[−0.554, −0.442]	[−0.681, −0.489]	[−0.630, −0.545]	[−0.624, −0.486]	[−0.638, −0.518]	[−0.634, −0.528]	[−0.596, −0.498]
Intercept	z =−11.11, $p < 0.001$	z =−14.85, $p < 0.001$	z =−29.41, $p < 0.001$	z =−2.04, p =0.041	z =−21.90, $p < 0.001$	z =−19.69, $p < 0.001$	z =−14.59, $p < 0.001$	z =−9.70, $p < 0.001$
	[−1.073, −0.669]	[−1.426, −1.005]	[−3.109, −2.609]	[−0.325, 0.037]	[−2.147, −1.695]	[−1.941, −1.492]	[−1.352, −0.946]	[−0.897, −0.521]
Post-level RE	✓	✓	✓	✓	✓	✓	✓	✓
#Observations	25,296	25,296	25,296	25,296	25,296	25,296	25,296	25,296

Table S63: Regression results for two-period ATT estimations, based on posts from accounts with lower followee counts and across reposter characteristics. Post-specific random effects are included. Reported are coefficient estimates with standard errors in parentheses. * $p < 0.01$, ** $p < 0.005$, *** $p < 0.001$. Exact z statistics, p values and 99% CIs are reported in Table S64.

	PriorInteraction		Verified		Political leaning		Misinformation exposure	
	(1)	(2)	(3)	(4)	(5)	(6)	(7)	(8)
	Yes	No	Yes	No	Left	Right	High	Low
Display	0.267	0.935***	0.414***	0.636***	0.570***	0.651***	0.606***	0.629***
	(0.117)	(0.107)	(0.120)	(0.098)	(0.111)	(0.114)	(0.106)	(0.101)
After	−0.038	0.140***	−0.112	0.034	0.037	0.038	0.040	0.016
	(0.028)	(0.034)	(0.058)	(0.026)	(0.038)	(0.037)	(0.033)	(0.029)
Display × After	−0.805***	−1.153***	−0.763***	−0.978***	−1.003***	−0.887***	−0.936***	−0.967***
	(0.030)	(0.036)	(0.063)	(0.028)	(0.041)	(0.039)	(0.034)	(0.031)
PostAge	−0.620***	−0.506***	−0.553***	−0.576***	−0.559***	−0.580***	−0.572***	−0.547***
	(0.018)	(0.020)	(0.037)	(0.016)	(0.024)	(0.023)	(0.020)	(0.018)
Intercept	−1.117***	−1.331***	−3.173***	−0.216**	−2.002***	−1.953***	−1.351***	−0.682***
	(0.086)	(0.080)	(0.103)	(0.072)	(0.085)	(0.087)	(0.080)	(0.074)
Post-level RE	✓	✓	✓	✓	✓	✓	✓	✓
#Observations	25,312	25,312	25,312	25,312	25,312	25,312	25,312	25,312

Table S64: Details of z statistics, p values and 99% CIs (in brackets) for the coefficient estimates reported in Table S63.

	PriorInteraction		Verified		Political leaning		Misinformation exposure	
	(1)	(2)	(3)	(4)	(5)	(6)	(7)	(8)
	Yes	No	Yes	No	Left	Right	High	Low
Display	z =2.29, p =0.022	z =8.74, $p < 0.001$	z =3.45, $p < 0.001$	z =6.48, $p < 0.001$	z =5.16, $p < 0.001$	z =5.74, $p < 0.001$	z =5.70, $p < 0.001$	z =6.25, $p < 0.001$
	[−0.033, 0.568]	[0.659, 1.211]	[0.104, 0.723]	[0.383, 0.889]	[0.285, 0.855]	[0.359, 0.944]	[0.332, 0.880]	[0.370, 0.888]
After	z =−1.35, p =0.178	z =4.06, $p < 0.001$	z =−1.94, p =0.052	z =1.28, p =0.199	z =0.97, p =0.331	z =1.03, p =0.305	z =1.22, p =0.222	z =0.55, p =0.580
	[−0.112, 0.035]	[0.051, 0.229]	[−0.260, 0.037]	[−0.034, 0.102]	[−0.062, 0.137]	[−0.057, 0.133]	[−0.044, 0.124]	[−0.059, 0.091]
Display×After	z =−26.46, $p < 0.001$	z =−31.72, $p < 0.001$	z =−12.14, $p < 0.001$	z =−34.96, $p < 0.001$	z =−24.30, $p < 0.001$	z =−22.74, $p < 0.001$	z =−27.14, $p < 0.001$	z =−31.36, $p < 0.001$
	[−0.883, −0.726]	[−1.247, −1.059]	[−0.925, −0.601]	[−1.050, −0.906]	[−1.109, −0.897]	[−0.988, −0.787]	[−1.025, −0.847]	[−1.046, −0.887]
PostAge	z =−34.46, $p < 0.001$	z =−24.69, $p < 0.001$	z =−15.07, $p < 0.001$	z =−36.09, $p < 0.001$	z =−23.00, $p < 0.001$	z =−25.41, $p < 0.001$	z =−28.35, $p < 0.001$	z =−30.83, $p < 0.001$
	[−0.666, −0.573]	[−0.559, −0.453]	[−0.648, −0.459]	[−0.617, −0.535]	[−0.621, −0.496]	[−0.639, −0.521]	[−0.624, −0.520]	[−0.592, −0.501]
Intercept	z =−13.01, $p < 0.001$	z =−16.61, $p < 0.001$	z =−30.81, $p < 0.001$	z =−3.00, p =0.003	z =−23.51, $p < 0.001$	z =−22.52, $p < 0.001$	z =−16.98, $p < 0.001$	z =−9.17, $p < 0.001$
	[−1.338, −0.896]	[−1.538, −1.125]	[−3.438, −2.907]	[−0.401, −0.030]	[−2.221, −1.783]	[−2.177, −1.730]	[−1.555, −1.146]	[−0.873, −0.490]
Post-level RE	✓	✓	✓	✓	✓	✓	✓	✓
#Observations	25,312	25,312	25,312	25,312	25,312	25,312	25,312	25,312

Table S65: Regression results for two-period ATT estimations, based on longer posts and across reposter characteristics. Post-specific random effects are included. Reported are coefficient estimates with standard errors in parentheses. * $p < 0.01$, ** $p < 0.005$, *** $p < 0.001$. Exact z statistics, p values and 99% CIs are reported in Table S66.

	PriorInteraction		Verified		Political leaning		Misinformation exposure	
	(1)	(2)	(3)	(4)	(5)	(6)	(7)	(8)
	Yes	No	Yes	No	Left	Right	High	Low
Display	0.081	0.648***	0.245	0.396***	0.255	0.637***	0.519***	0.332***
	(0.109)	(0.107)	(0.117)	(0.095)	(0.111)	(0.114)	(0.104)	(0.098)
After	−0.092**	0.047	−0.062	−0.046	−0.084	−0.028	−0.037	−0.058
	(0.028)	(0.035)	(0.054)	(0.026)	(0.040)	(0.036)	(0.032)	(0.030)
Display × After	−0.776***	−0.996***	−0.808***	−0.869***	−0.821***	−0.854***	−0.860***	−0.857***
	(0.030)	(0.037)	(0.060)	(0.028)	(0.043)	(0.038)	(0.033)	(0.032)
PostAge	−0.656***	−0.576***	−0.682***	−0.624***	−0.608***	−0.624***	−0.627***	−0.598***
	(0.018)	(0.022)	(0.036)	(0.016)	(0.026)	(0.023)	(0.020)	(0.019)
Intercept	−0.839***	−1.344***	−2.933***	−0.153	−1.926***	−1.792***	−1.158***	−0.711***
	(0.080)	(0.080)	(0.098)	(0.070)	(0.086)	(0.087)	(0.078)	(0.072)
Post-level RE	✓	✓	✓	✓	✓	✓	✓	✓
#Observations	24,416	24,416	24,416	24,416	24,416	24,416	24,416	24,416

Table S66: Details of z statistics, p values and 99% CIs (in brackets) for the coefficient estimates reported in Table S65.

	PriorInteraction		Verified		Political leaning		Misinformation exposure	
	(1)	(2)	(3)	(4)	(5)	(6)	(7)	(8)
	Yes	No	Yes	No	Left	Right	High	Low
Display	z =0.75, p =0.456	z =6.09, $p < 0.001$	z =2.08, p =0.037	z =4.17, $p < 0.001$	z =2.30, p =0.022	z =5.58, $p < 0.001$	z =5.01, $p < 0.001$	z =3.40, $p < 0.001$
	[−0.199, 0.362]	[0.374, 0.923]	[−0.058, 0.547]	[0.151, 0.640]	[−0.031, 0.541]	[0.343, 0.931]	[0.252, 0.786]	[0.081, 0.584]
After	z =−3.28, p =0.001	z =1.32, p =0.186	z =−1.13, p =0.257	z =−1.78, p =0.075	z =−2.09, p =0.037	z =−0.79, p =0.432	z =−1.17, p =0.242	z =−1.96, p =0.050
	[−0.165, −0.020]	[−0.044, 0.138]	[−0.202, 0.078]	[−0.113, 0.021]	[−0.187, 0.020]	[−0.121, 0.064]	[−0.118, 0.044]	[−0.135, 0.018]
Display×After	z =−25.63, $p < 0.001$	z =−26.77, $p < 0.001$	z =−13.55, $p < 0.001$	z =−31.49, $p < 0.001$	z =−18.92, $p < 0.001$	z =−22.61, $p < 0.001$	z =−25.74, $p < 0.001$	z =−27.04, $p < 0.001$
	[−0.854, −0.698]	[−1.092, −0.900]	[−0.961, −0.654]	[−0.940, −0.798]	[−0.932, −0.709]	[−0.951, −0.757]	[−0.946, −0.774]	[−0.938, −0.775]
PostAge	z =−35.95, $p < 0.001$	z =−26.46, $p < 0.001$	z =−18.97, $p < 0.001$	z =−38.43, $p < 0.001$	z =−23.35, $p < 0.001$	z =−27.68, $p < 0.001$	z =−31.35, $p < 0.001$	z =−31.70, $p < 0.001$
	[−0.703, −0.609]	[−0.632, −0.520]	[−0.775, −0.590]	[−0.666, −0.582]	[−0.675, −0.541]	[−0.682, −0.566]	[−0.679, −0.576]	[−0.647, −0.550]
Intercept	z =−10.49, $p < 0.001$	z =−16.75, $p < 0.001$	z =−29.87, $p < 0.001$	z =−2.19, p =0.028	z =−22.47, $p < 0.001$	z =−20.62, $p < 0.001$	z =−14.92, $p < 0.001$	z =−9.82, $p < 0.001$
	[−1.045, −0.633]	[−1.550, −1.137]	[−3.185, −2.680]	[−0.332, 0.027]	[−2.146, −1.705]	[−2.015, −1.568]	[−1.357, −0.958]	[−0.897, −0.525]
Post-level RE	✓	✓	✓	✓	✓	✓	✓	✓
#Observations	24,416	24,416	24,416	24,416	24,416	24,416	24,416	24,416

Table S67: Regression results for two-period ATT estimations, based on shorter posts and across reposter characteristics. Post-specific random effects are included. Reported are coefficient estimates with standard errors in parentheses. * $p < 0.01$, ** $p < 0.005$, *** $p < 0.001$. Exact z statistics, p values and 99% CIs are reported in Table S68.

	PriorInteraction		Verified		Political leaning		Misinformation exposure	
	(1)	(2)	(3)	(4)	(5)	(6)	(7)	(8)
	Yes	No	Yes	No	Left	Right	High	Low
Display	0.585***	1.048***	0.697***	0.856***	0.694***	0.852***	0.860***	0.840***
	(0.110)	(0.106)	(0.115)	(0.096)	(0.109)	(0.111)	(0.104)	(0.098)
After	0.016	0.110**	−0.149	0.055	0.059	0.027	0.039	0.056
	(0.030)	(0.036)	(0.065)	(0.028)	(0.041)	(0.039)	(0.035)	(0.030)
Display × After	−0.986***	−1.284***	−1.024***	−1.136***	−1.134***	−1.117***	−1.146***	−1.113***
	(0.032)	(0.037)	(0.069)	(0.029)	(0.043)	(0.041)	(0.036)	(0.031)
PostAge	−0.612***	−0.444***	−0.457***	−0.547***	−0.513***	−0.538***	−0.531***	−0.508***
	(0.019)	(0.020)	(0.038)	(0.016)	(0.025)	(0.024)	(0.021)	(0.018)
Intercept	−1.149***	−1.218***	−3.103***	−0.218**	−2.011***	−1.889***	−1.356***	−0.688***
	(0.084)	(0.081)	(0.102)	(0.072)	(0.087)	(0.087)	(0.081)	(0.075)
Post-level RE	✓	✓	✓	✓	✓	✓	✓	✓
#Observations	26,192	26,192	26,192	26,192	26,192	26,192	26,192	26,192

Table S68: Details of z statistics, p values and 99% CIs (in brackets) for the coefficient estimates reported in Table S67.

	PriorInteraction		Verified		Political leaning		Misinformation exposure	
	(1)	(2)	(3)	(4)	(5)	(6)	(7)	(8)
	Yes	No	Yes	No	Left	Right	High	Low
Display	z =5.29, $p < 0.001$	z =9.93, $p < 0.001$	z =6.06, $p < 0.001$	z =8.96, $p < 0.001$	z =6.34, $p < 0.001$	z =7.71, $p < 0.001$	z =8.24, $p < 0.001$	z =8.59, $p < 0.001$
	[0.300, 0.869]	[0.776, 1.320]	[0.401, 0.994]	[0.610, 1.102]	[0.412, 0.976]	[0.567, 1.137]	[0.591, 1.128]	[0.588, 1.092]
After	z =0.52, p =0.602	z =3.10, p =0.002	z =−2.30, p =0.021	z =1.98, p =0.047	z =1.44, p =0.151	z =0.69, p =0.488	z =1.10, p =0.270	z =1.86, p =0.064
	[−0.063, 0.094]	[0.018, 0.201]	[−0.315, 0.018]	[−0.016, 0.126]	[−0.047, 0.165]	[−0.074, 0.129]	[−0.051, 0.128]	[−0.022, 0.134]
Display×After	z =−31.11, $p < 0.001$	z =−35.00, $p < 0.001$	z =−14.92, $p < 0.001$	z =−39.56, $p < 0.001$	z =−26.51, $p < 0.001$	z =−27.41, $p < 0.001$	z =−31.76, $p < 0.001$	z =−35.56, $p < 0.001$
	[−1.068, −0.904]	[−1.378, −1.189]	[−1.201, −0.848]	[−1.210, −1.062]	[−1.244, −1.024]	[−1.222, −1.012]	[−1.239, −1.053]	[−1.194, −1.033]
PostAge	z =−32.88, $p < 0.001$	z =−21.81, $p < 0.001$	z =−11.95, $p < 0.001$	z =−33.90, $p < 0.001$	z =−20.69, $p < 0.001$	z =−22.79, $p < 0.001$	z =−25.46, $p < 0.001$	z =−28.72, $p < 0.001$
	[−0.660, −0.564]	[−0.496, −0.391]	[−0.555, −0.358]	[−0.589, −0.506]	[−0.577, −0.450]	[−0.599, −0.477]	[−0.584, −0.477]	[−0.554, −0.463]
Intercept	z =−13.63, $p < 0.001$	z =−14.96, $p < 0.001$	z =−30.46, $p < 0.001$	z =−3.01, p =0.003	z =−23.14, $p < 0.001$	z =−21.65, $p < 0.001$	z =−16.79, $p < 0.001$	z =−9.23, $p < 0.001$
	[−1.366, −0.932]	[−1.427, −1.008]	[−3.365, −2.840]	[−0.404, −0.031]	[−2.235, −1.788]	[−2.114, −1.664]	[−1.564, −1.148]	[−0.880, −0.496]
Post-level RE	✓	✓	✓	✓	✓	✓	✓	✓
#Observations	26,192	26,192	26,192	26,192	26,192	26,192	26,192	26,192

Table S69: Regression results for two-period ATT estimations, based on media posts and across reposter characteristics. Post-specific random effects are included. Reported are coefficient estimates with standard errors in parentheses. * $p < 0.01$, ** $p < 0.005$, *** $p < 0.001$. Exact z statistics, p values and 99% CIs are reported in Table S70.

	PriorInteraction		Verified		Political leaning		Misinformation exposure	
	(1)	(2)	(3)	(4)	(5)	(6)	(7)	(8)
	Yes	No	Yes	No	Left	Right	High	Low
Display	0.373***	0.947***	0.530***	0.693***	0.574***	0.754***	0.730***	0.673***
	(0.091)	(0.087)	(0.095)	(0.078)	(0.090)	(0.092)	(0.086)	(0.080)
After	−0.041	0.090**	−0.074	0.005	0.014	0.003	0.005	0.005
	(0.024)	(0.029)	(0.049)	(0.022)	(0.034)	(0.031)	(0.027)	(0.024)
Display × After	−0.917***	−1.207***	−0.978***	−1.053***	−1.049***	−1.029***	−1.048***	−1.038***
	(0.025)	(0.030)	(0.053)	(0.023)	(0.035)	(0.032)	(0.028)	(0.025)
PostAge	−0.613***	−0.491***	−0.572***	−0.569***	−0.554***	−0.559***	−0.564***	−0.538***
	(0.015)	(0.017)	(0.031)	(0.013)	(0.021)	(0.019)	(0.017)	(0.015)
Intercept	−0.891***	−1.203***	−2.980***	−0.096	−1.931***	−1.725***	−1.190***	−0.584***
	(0.068)	(0.067)	(0.082)	(0.059)	(0.071)	(0.071)	(0.066)	(0.060)
Post-level RE	✓	✓	✓	✓	✓	✓	✓	✓
#Observations	36,880	36,880	36,880	36,880	36,880	36,880	36,880	36,880

Table S70: Details of z statistics, p values and 99% CIs (in brackets) for the coefficient estimates reported in Table S69.

	PriorInteraction		Verified		Political leaning		Misinformation exposure	
	(1)	(2)	(3)	(4)	(5)	(6)	(7)	(8)
	Yes	No	Yes	No	Left	Right	High	Low
Display	z =4.11, $p < 0.001$	z =10.89, $p < 0.001$	z =5.58, $p < 0.001$	z =8.83, $p < 0.001$	z =6.36, $p < 0.001$	z =8.22, $p < 0.001$	z =8.50, $p < 0.001$	z =8.43, $p < 0.001$
	[0.140, 0.607]	[0.723, 1.171]	[0.285, 0.774]	[0.491, 0.896]	[0.342, 0.807]	[0.518, 0.991]	[0.509, 0.951]	[0.467, 0.879]
After	z =−1.70, p =0.088	z =3.12, p =0.002	z =−1.51, p =0.132	z =0.23, p =0.816	z =0.43, p =0.669	z =0.09, p =0.929	z =0.17, p =0.863	z =0.19, p =0.845
	[−0.102, 0.021]	[0.016, 0.165]	[−0.201, 0.053]	[−0.051, 0.062]	[−0.072, 0.101]	[−0.076, 0.082]	[−0.065, 0.075]	[−0.058, 0.067]
Display×After	z =−36.65, $p < 0.001$	z =−40.19, $p < 0.001$	z =−18.52, $p < 0.001$	z =−45.94, $p < 0.001$	z =−29.69, $p < 0.001$	z =−32.29, $p < 0.001$	z =−37.05, $p < 0.001$	z =−41.01, $p < 0.001$
	[−0.981, −0.853]	[−1.285, −1.130]	[−1.114, −0.842]	[−1.112, −0.994]	[−1.140, −0.958]	[−1.111, −0.947]	[−1.121, −0.975]	[−1.104, −0.973]
PostAge	z =−41.26, $p < 0.001$	z =−28.90, $p < 0.001$	z =−18.71, $p < 0.001$	z =−43.37, $p < 0.001$	z =−26.59, $p < 0.001$	z =−29.84, $p < 0.001$	z =−33.90, $p < 0.001$	z =−36.79, $p < 0.001$
	[−0.651, −0.575]	[−0.535, −0.447]	[−0.651, −0.494]	[−0.602, −0.535]	[−0.608, −0.500]	[−0.607, −0.510]	[−0.606, −0.521]	[−0.575, −0.500]
Intercept	z =−13.03, $p < 0.001$	z =−18.06, $p < 0.001$	z =−36.29, $p < 0.001$	z =−1.63, p =0.104	z =−27.14, $p < 0.001$	z =−24.17, $p < 0.001$	z =−18.11, $p < 0.001$	z =−9.66, $p < 0.001$
	[−1.067, −0.715]	[−1.375, −1.032]	[−3.191, −2.768]	[−0.248, 0.056]	[−2.114, −1.747]	[−1.909, −1.541]	[−1.359, −1.021]	[−0.739, −0.428]
Post-level RE	✓	✓	✓	✓	✓	✓	✓	✓
#Observations	36,880	36,880	36,880	36,880	36,880	36,880	36,880	36,880

Table S71: Regression results for two-period ATT estimations, based on non-media posts and across reposter characteristics. Post-specific random effects are included. Reported are coefficient estimates with standard errors in parentheses. * $p < 0.01$, ** $p < 0.005$, *** $p < 0.001$. Exact z statistics, p values and 99% CIs are reported in Table S72.

	PriorInteraction		Verified		Political leaning		Misinformation exposure	
	(1)	(2)	(3)	(4)	(5)	(6)	(7)	(8)
	Yes	No	Yes	No	Left	Right	High	Low
Display	0.184	0.591***	0.250	0.439***	0.198	0.673***	0.544***	0.347
	(0.149)	(0.148)	(0.164)	(0.130)	(0.155)	(0.157)	(0.142)	(0.137)
After	−0.042	0.025	−0.210*	−0.013	−0.100	−0.026	−0.030	−0.033
	(0.043)	(0.051)	(0.079)	(0.039)	(0.056)	(0.054)	(0.047)	(0.045)
Display × After	−0.766***	−0.951***	−0.683***	−0.855***	−0.782***	−0.844***	−0.857***	−0.819***
	(0.046)	(0.054)	(0.088)	(0.041)	(0.060)	(0.057)	(0.050)	(0.048)
PostAge	−0.692***	−0.536***	−0.563***	−0.623***	−0.564***	−0.642***	−0.616***	−0.579***
	(0.027)	(0.031)	(0.051)	(0.024)	(0.036)	(0.034)	(0.030)	(0.028)
Intercept	−1.238***	−1.462***	−3.105***	−0.399***	−2.051***	−2.131***	−1.408***	−0.984***
	(0.110)	(0.112)	(0.139)	(0.096)	(0.119)	(0.121)	(0.107)	(0.102)
Post-level RE	✓	✓	✓	✓	✓	✓	✓	✓
#Observations	13,728	13,728	13,728	13,728	13,728	13,728	13,728	13,728

Table S72: Details of z statistics, p values and 99% CIs (in brackets) for the coefficient estimates reported in Table S71.

	PriorInteraction		Verified		Political leaning		Misinformation exposure	
	(1)	(2)	(3)	(4)	(5)	(6)	(7)	(8)
	Yes	No	Yes	No	Left	Right	High	Low
Display	z =1.24, p =0.215	z =3.99, p < 0.001	z =1.52, p =0.128	z =3.37, p < 0.001	z =1.28, p =0.199	z =4.28, p < 0.001	z =3.82, p < 0.001	z =2.53, p =0.011
	[−0.199, 0.567]	[0.210, 0.972]	[−0.173, 0.672]	[0.103, 0.775]	[−0.200, 0.597]	[0.268, 1.079]	[0.177, 0.910]	[−0.006, 0.700]
After	z =−0.99, p =0.322	z =0.50, p =0.618	z =−2.64, p =0.008	z =−0.32, p =0.746	z =−1.78, p =0.076	z =−0.48, p =0.630	z =−0.63, p =0.531	z =−0.73, p =0.463
	[−0.152, 0.068]	[−0.106, 0.156]	[−0.415, −0.005]	[−0.112, 0.087]	[−0.245, 0.045]	[−0.165, 0.113]	[−0.152, 0.092]	[−0.148, 0.082]
Display×After	z =−16.71, p < 0.001	z =−17.68, p < 0.001	z =−7.78, p < 0.001	z =−20.78, p < 0.001	z =−12.94, p < 0.001	z =−14.80, p < 0.001	z =−17.09, p < 0.001	z =−17.16, p < 0.001
	[−0.884, −0.648]	[−1.089, −0.812]	[−0.910, −0.457]	[−0.961, −0.749]	[−0.938, −0.626]	[−0.990, −0.697]	[−0.986, −0.728]	[−0.942, −0.696]
PostAge	z =−25.25, p < 0.001	z =−17.29, p < 0.001	z =−10.99, p < 0.001	z =−26.09, p < 0.001	z =−15.76, p < 0.001	z =−19.13, p < 0.001	z =−20.79, p < 0.001	z =−20.81, p < 0.001
	[−0.763, −0.622]	[−0.616, −0.457]	[−0.694, −0.431]	[−0.684, −0.561]	[−0.657, −0.472]	[−0.729, −0.556]	[−0.692, −0.540]	[−0.650, −0.507]
Intercept	z =−11.27, p < 0.001	z =−13.10, p < 0.001	z =−22.30, p < 0.001	z =−4.16, p < 0.001	z =−17.22, p < 0.001	z =−17.57, p < 0.001	z =−13.16, p < 0.001	z =−9.64, p < 0.001
	[−1.521, −0.955]	[−1.750, −1.175]	[−3.464, −2.747]	[−0.646, −0.152]	[−2.358, −1.744]	[−2.444, −1.819]	[−1.684, −1.132]	[−1.247, −0.721]
Post-level RE	✓	✓	✓	✓	✓	✓	✓	✓
#Observations	13,728	13,728	13,728	13,728	13,728	13,728	13,728	13,728

Table S73: Regression results for two-period ATT estimations, based on high-positive posts and across reposter characteristics. Post-specific random effects are included. Reported are coefficient estimates with standard errors in parentheses. * $p < 0.01$, ** $p < 0.005$, *** $p < 0.001$. Exact z statistics, p values and 99% CIs are reported in Table S74.

	PriorInteraction		Verified		Political leaning		Misinformation exposure	
	(1)	(2)	(3)	(4)	(5)	(6)	(7)	(8)
	Yes	No	Yes	No	Left	Right	High	Low
Display	0.493***	1.007***	0.689***	0.822***	0.674***	0.904***	0.857***	0.804***
	(0.110)	(0.105)	(0.121)	(0.095)	(0.112)	(0.114)	(0.105)	(0.097)
After	−0.057	0.068	−0.132	−0.008	−0.013	−0.025	−0.009	−0.003
	(0.032)	(0.036)	(0.065)	(0.028)	(0.042)	(0.041)	(0.036)	(0.031)
Display × After	−0.893***	−1.154***	−0.953***	−1.016***	−0.979***	−1.023***	−1.047***	−0.991***
	(0.033)	(0.038)	(0.069)	(0.030)	(0.044)	(0.043)	(0.038)	(0.033)
PostAge	−0.569***	−0.461***	−0.515***	−0.531***	−0.537***	−0.503***	−0.511***	−0.511***
	(0.020)	(0.021)	(0.039)	(0.017)	(0.026)	(0.025)	(0.022)	(0.019)
Intercept	−1.240***	−1.368***	−3.314***	−0.373***	−2.167***	−2.127***	−1.518***	−0.851***
	(0.083)	(0.080)	(0.106)	(0.071)	(0.088)	(0.089)	(0.080)	(0.073)
Post-level RE	✓	✓	✓	✓	✓	✓	✓	✓
#Observations	25,296	25,296	25,296	25,296	25,296	25,296	25,296	25,296

Table S74: Details of z statistics, p values and 99% CIs (in brackets) for the coefficient estimates reported in Table S73.

	PriorInteraction		Verified		Political leaning		Misinformation exposure	
	(1)	(2)	(3)	(4)	(5)	(6)	(7)	(8)
	Yes	No	Yes	No	Left	Right	High	Low
Display	z =4.49, p < 0.001	z =9.55, p < 0.001	z =5.71, p < 0.001	z =8.69, p < 0.001	z =6.04, p < 0.001	z =7.96, p < 0.001	z =8.20, p < 0.001	z =8.29, p < 0.001
	[0.210, 0.776]	[0.735, 1.278]	[0.378, 0.999]	[0.578, 1.065]	[0.387, 0.962]	[0.612, 1.197]	[0.588, 1.126]	[0.555, 1.054]
After	z =−1.80, p =0.071	z =1.88, p =0.060	z =−2.04, p =0.041	z =−0.30, p =0.767	z =−0.31, p =0.760	z =−0.61, p =0.540	z =−0.26, p =0.795	z =−0.10, p =0.919
	[−0.139, 0.024]	[−0.025, 0.162]	[−0.299, 0.034]	[−0.081, 0.064]	[−0.121, 0.095]	[−0.130, 0.080]	[−0.102, 0.084]	[−0.083, 0.077]
Display×After	z =−26.81, p < 0.001	z =−30.67, p < 0.001	z =−13.80, p < 0.001	z =−34.42, p < 0.001	z =−22.25, p < 0.001	z =−24.05, p < 0.001	z =−27.91, p < 0.001	z =−30.49, p < 0.001
	[−0.979, −0.808]	[−1.251, −1.057]	[−1.131, −0.775]	[−1.092, −0.940]	[−1.092, −0.866]	[−1.132, −0.913]	[−1.143, −0.950]	[−1.075, −0.907]
PostAge	z =−28.96, p < 0.001	z =−21.74, p < 0.001	z =−13.04, p < 0.001	z =−31.64, p < 0.001	z =−20.71, p < 0.001	z =−20.43, p < 0.001	z =−23.49, p < 0.001	z =−27.45, p < 0.001
	[−0.619, −0.518]	[−0.516, −0.406]	[−0.617, −0.413]	[−0.575, −0.488]	[−0.604, −0.470]	[−0.566, −0.439]	[−0.567, −0.455]	[−0.559, −0.463]
Intercept	z =−15.03, p < 0.001	z =−17.06, p < 0.001	z =−31.15, p < 0.001	z =−5.29, p < 0.001	z =−24.65, p < 0.001	z =−23.90, p < 0.001	z =−18.97, p < 0.001	z =−11.66, p < 0.001
	[−1.453, −1.028]	[−1.574, −1.161]	[−3.588, −3.040]	[−0.555, −0.191]	[−2.394, −1.941]	[−2.356, −1.898]	[−1.725, −1.312]	[−1.039, −0.663]
Post-level RE	✓	✓	✓	✓	✓	✓	✓	✓
#Observations	25,296	25,296	25,296	25,296	25,296	25,296	25,296	25,296

Table S75: Regression results for two-period ATT estimations, based on low-positive posts and across reposter characteristics. Post-specific random effects are included. Reported are coefficient estimates with standard errors in parentheses. $^{*}\ p < 0.01$, $^{**}\ p < 0.005$, $^{***}\ p < 0.001$. Exact z statistics, p values and 99% CIs are reported in Table S76.

	PriorInteraction		Verified		Political leaning		Misinformation exposure	
	(1) Yes	(2) No	(3) Yes	(4) No	(5) Left	(6) Right	(7) High	(8) Low
Display	0.172	0.722***	0.254	0.448***	0.297*	0.581***	0.519***	0.402***
	(0.109)	(0.107)	(0.112)	(0.096)	(0.109)	(0.110)	(0.103)	(0.099)
After	−0.028	0.079	−0.093	0.010	−0.019	0.014	0.002	−0.004
	(0.027)	(0.035)	(0.055)	(0.026)	(0.040)	(0.035)	(0.031)	(0.029)
Display × After	−0.873***	−1.141***	−0.867***	−1.001***	−0.986***	−0.956***	−0.968***	−0.993***
	(0.029)	(0.037)	(0.060)	(0.027)	(0.042)	(0.037)	(0.033)	(0.031)
PostAge	−0.685***	−0.543***	−0.614***	−0.628***	−0.577***	−0.640***	−0.630***	−0.582***
	(0.017)	(0.021)	(0.035)	(0.016)	(0.025)	(0.022)	(0.019)	(0.018)
Intercept	−0.728***	−1.182***	−2.718***	0.017	−1.763***	−1.538***	−0.978***	−0.539***
	(0.082)	(0.082)	(0.094)	(0.072)	(0.085)	(0.085)	(0.078)	(0.074)
Post-level RE	✓	✓	✓	✓	✓	✓	✓	✓
#Observations	25,312	25,312	25,312	25,312	25,312	25,312	25,312	25,312

Table S76: Details of z statistics, p values and 99% CIs (in brackets) for the coefficient estimates reported in Table S75.

	PriorInteraction		Verified		Political leaning		Misinformation exposure	
	(1) Yes	(2) No	(3) Yes	(4) No	(5) Left	(6) Right	(7) High	(8) Low
Display	z =1.58, p =0.114	z =6.73, $p < 0.001$	z =2.27, p =0.023	z =4.68, $p < 0.001$	z =2.73, p =0.006	z =5.26, $p < 0.001$	z =5.05, $p < 0.001$	z =4.06, $p < 0.001$
	[−0.109, 0.453]	[0.446, 0.998]	[−0.034, 0.542]	[0.201, 0.695]	[0.016, 0.578]	[0.297, 0.865]	[0.255, 0.784]	[0.147, 0.656]
After	z =−1.01, p =0.314	z =2.26, p =0.024	z =−1.69, p =0.092	z =0.38, p =0.703	z =−0.47, p =0.638	z =0.40, p =0.691	z =0.07, p =0.943	z =−0.14, p =0.892
	[−0.098, 0.043]	[−0.011, 0.168]	[−0.234, 0.049]	[−0.057, 0.076]	[−0.121, 0.084]	[−0.077, 0.105]	[−0.078, 0.082]	[−0.079, 0.071]
Display×After	z =−29.98, $p < 0.001$	z =−31.26, $p < 0.001$	z =−14.50, $p < 0.001$	z =−36.80, $p < 0.001$	z =−23.32, $p < 0.001$	z =−26.00, $p < 0.001$	z =−29.65, $p < 0.001$	z =−32.25, $p < 0.001$
	[−0.948, −0.798]	[−1.235, −1.047]	[−1.021, −0.713]	[−1.071, −0.931]	[−1.095, −0.877]	[−1.051, −0.861]	[−1.052, −0.884]	[−1.072, −0.913]
PostAge	z =−39.18, $p < 0.001$	z =−25.93, $p < 0.001$	z =−17.48, $p < 0.001$	z =−39.90, $p < 0.001$	z =−23.02, $p < 0.001$	z =−29.28, $p < 0.001$	z =−32.47, $p < 0.001$	z =−32.39, $p < 0.001$
	[−0.730, −0.640]	[−0.597, −0.489]	[−0.704, −0.523]	[−0.669, −0.588]	[−0.641, −0.512]	[−0.697, −0.584]	[−0.680, −0.580]	[−0.629, −0.536]
Intercept	z =−8.91, $p < 0.001$	z =−14.43, $p < 0.001$	z =−28.86, $p < 0.001$	z =0.23, p =0.815	z =−20.76, $p < 0.001$	z =−18.10, $p < 0.001$	z =−12.52, $p < 0.001$	z =−7.23, $p < 0.001$
	[−0.938, −0.517]	[−1.392, −0.971]	[−2.960, −2.475]	[−0.168, 0.201]	[−1.982, −1.545]	[−1.757, −1.319]	[−1.179, −0.776]	[−0.730, −0.347]
Post-level RE	✓	✓	✓	✓	✓	✓	✓	✓
#Observations	25,312	25,312	25,312	25,312	25,312	25,312	25,312	25,312

Table S77: Regression results for two-period ATT estimations, based on high-negative posts and across reposter characteristics. Post-specific random effects are included. Reported are coefficient estimates with standard errors in parentheses. * $p < 0.01$, ** $p < 0.005$, *** $p < 0.001$. Exact z statistics, p values and 99% CIs are reported in Table S78.

	PriorInteraction		Verified		Political leaning		Misinformation exposure	
	(1)	(2)	(3)	(4)	(5)	(6)	(7)	(8)
	Yes	No	Yes	No	Left	Right	High	Low
Display	0.098	0.624***	0.238	0.400***	0.280	0.506***	0.450***	0.370***
	(0.111)	(0.106)	(0.112)	(0.096)	(0.110)	(0.110)	(0.102)	(0.100)
After	−0.060	0.014	−0.054	−0.036	−0.059	−0.038	−0.042	−0.038
	(0.027)	(0.034)	(0.054)	(0.026)	(0.039)	(0.035)	(0.031)	(0.029)
Display × After	−0.866***	−1.098***	−0.905***	−0.985***	−0.966***	−0.970***	−0.976***	−0.968***
	(0.029)	(0.036)	(0.060)	(0.027)	(0.042)	(0.037)	(0.033)	(0.031)
PostAge	−0.657***	−0.502***	−0.612***	−0.593***	−0.559***	−0.586***	−0.584***	−0.562***
	(0.017)	(0.021)	(0.035)	(0.016)	(0.025)	(0.022)	(0.019)	(0.018)
Intercept	−0.692***	−1.045***	−2.726***	0.091	−1.699***	−1.470***	−0.899***	−0.478***
	(0.082)	(0.080)	(0.093)	(0.071)	(0.085)	(0.084)	(0.077)	(0.075)
Post-level RE	✓	✓	✓	✓	✓	✓	✓	✓
#Observations	25,296	25,296	25,296	25,296	25,296	25,296	25,296	25,296

Table S78: Details of z statistics, p values and 99% CIs (in brackets) for the coefficient estimates reported in Table S77.

	PriorInteraction		Verified		Political leaning		Misinformation exposure	
	(1)	(2)	(3)	(4)	(5)	(6)	(7)	(8)
	Yes	No	Yes	No	Left	Right	High	Low
Display	z =0.88, p =0.377	z =5.87, $p < 0.001$	z =2.13, p =0.033	z =4.16, $p < 0.001$	z =2.55, p =0.011	z =4.59, $p < 0.001$	z =4.40, $p < 0.001$	z =3.71, $p < 0.001$
	[−0.188, 0.383]	[0.350, 0.897]	[−0.049, 0.526]	[0.153, 0.648]	[−0.003, 0.564]	[0.222, 0.790]	[0.186, 0.714]	[0.113, 0.627]
After	z =−2.20, p =0.028	z =0.42, p =0.675	z =−1.00, p =0.319	z =−1.41, p =0.158	z =−1.52, p =0.129	z =−1.07, p =0.285	z =−1.36, p =0.174	z =−1.33, p =0.184
	[−0.131, 0.010]	[−0.074, 0.103]	[−0.195, 0.086]	[−0.103, 0.030]	[−0.159, 0.041]	[−0.128, 0.053]	[−0.122, 0.038]	[−0.113, 0.036]
Display×After	z =−29.44, $p < 0.001$	z =−30.37, $p < 0.001$	z =−15.02, $p < 0.001$	z =−36.02, $p < 0.001$	z =−23.21, $p < 0.001$	z =−26.05, $p < 0.001$	z =−29.61, $p < 0.001$	z =−31.44, $p < 0.001$
	[−0.942, −0.790]	[−1.191, −1.004]	[−1.060, −0.750]	[−1.055, −0.915]	[−1.073, −0.859]	[−1.066, −0.874]	[−1.060, −0.891]	[−1.047, −0.888]
PostAge	z =−37.64, $p < 0.001$	z =−24.31, $p < 0.001$	z =−17.55, $p < 0.001$	z =−37.71, $p < 0.001$	z =−22.71, $p < 0.001$	z =−26.64, $p < 0.001$	z =−30.00, $p < 0.001$	z =−31.51, $p < 0.001$
	[−0.702, −0.612]	[−0.556, −0.449]	[−0.702, −0.522]	[−0.633, −0.552]	[−0.622, −0.495]	[−0.643, −0.529]	[−0.634, −0.534]	[−0.608, −0.516]
Intercept	z =−8.41, $p < 0.001$	z =−13.00, $p < 0.001$	z =−29.23, $p < 0.001$	z =1.27, p =0.204	z =−20.02, $p < 0.001$	z =−17.45, $p < 0.001$	z =−11.65, $p < 0.001$	z =−6.41, $p < 0.001$
	[−0.904, −0.480]	[−1.251, −0.838]	[−2.966, −2.486]	[−0.093, 0.274]	[−1.918, −1.481]	[−1.687, −1.253]	[−1.098, −0.700]	[−0.670, −0.286]
Post-level RE	✓	✓	✓	✓	✓	✓	✓	✓
#Observations	25,296	25,296	25,296	25,296	25,296	25,296	25,296	25,296

Table S79: Regression results for two-period ATT estimations, based on low-negative posts and across reposter characteristics. Post-specific random effects are included. Reported are coefficient estimates with standard errors in parentheses. * $p < 0.01$, ** $p < 0.005$, *** $p < 0.001$. Exact z statistics, p values and 99% CIs are reported in Table S80.

	PriorInteraction		Verified		Political leaning		Misinformation exposure	
	(1)	(2)	(3)	(4)	(5)	(6)	(7)	(8)
	Yes	No	Yes	No	Left	Right	High	Low
Display	0.588***	1.121***	0.713***	0.886***	0.706***	1.006***	0.951***	0.848***
	(0.108)	(0.106)	(0.121)	(0.094)	(0.110)	(0.114)	(0.105)	(0.096)
After	−0.015	0.144***	−0.188**	0.047	0.039	0.044	0.050	0.038
	(0.032)	(0.037)	(0.066)	(0.028)	(0.043)	(0.041)	(0.036)	(0.032)
Display × After	−0.910***	−1.208***	−0.898***	−1.039***	−1.006***	−1.013***	−1.045***	−1.024***
	(0.033)	(0.038)	(0.069)	(0.029)	(0.045)	(0.042)	(0.037)	(0.033)
PostAge	−0.600***	−0.502***	−0.515***	−0.569***	−0.556***	−0.571***	−0.567***	−0.531***
	(0.020)	(0.021)	(0.040)	(0.017)	(0.026)	(0.024)	(0.022)	(0.019)
Intercept	−1.290***	−1.520***	−3.304***	−0.462***	−2.244***	−2.223***	−1.622***	−0.922***
	(0.082)	(0.082)	(0.107)	(0.071)	(0.088)	(0.090)	(0.081)	(0.073)
Post-level RE	✓	✓	✓	✓	✓	✓	✓	✓
#Observations	25,312	25,312	25,312	25,312	25,312	25,312	25,312	25,312

Table S80: Details of z statistics, p values and 99% CIs (in brackets) for the coefficient estimates reported in Table S79.

	PriorInteraction		Verified		Political leaning		Misinformation exposure	
	(1)	(2)	(3)	(4)	(5)	(6)	(7)	(8)
	Yes	No	Yes	No	Left	Right	High	Low
Display	z =5.45, $p < 0.001$	z =10.57, $p < 0.001$	z =5.91, $p < 0.001$	z =9.44, $p < 0.001$	z =6.41, $p < 0.001$	z =8.86, $p < 0.001$	z =9.09, $p < 0.001$	z =8.86, $p < 0.001$
	[0.310, 0.866]	[0.848, 1.394]	[0.402, 1.023]	[0.645, 1.128]	[0.422, 0.990]	[0.714, 1.299]	[0.682, 1.221]	[0.602, 1.095]
After	z =−0.46, p =0.648	z =3.88, $p < 0.001$	z =−2.87, p =0.004	z =1.64, p =0.100	z =0.92, p =0.359	z =1.06, p =0.288	z =1.37, p =0.171	z =1.21, p =0.227
	[−0.097, 0.068]	[0.048, 0.240]	[−0.357, −0.019]	[−0.027, 0.120]	[−0.071, 0.150]	[−0.062, 0.149]	[−0.044, 0.143]	[−0.043, 0.119]
Display×After	z =−27.43, $p < 0.001$	z =−31.62, $p < 0.001$	z =−13.02, $p < 0.001$	z =−35.27, $p < 0.001$	z =−22.45, $p < 0.001$	z =−24.04, $p < 0.001$	z =−28.00, $p < 0.001$	z =−31.36, $p < 0.001$
	[−0.995, −0.824]	[−1.307, −1.110]	[−1.075, −0.720]	[−1.115, −0.963]	[−1.122, −0.891]	[−1.121, −0.904]	[−1.141, −0.949]	[−1.108, −0.940]
PostAge	z =−30.48, $p < 0.001$	z =−23.35, $p < 0.001$	z =−12.94, $p < 0.001$	z =−33.81, $p < 0.001$	z =−21.06, $p < 0.001$	z =−23.35, $p < 0.001$	z =−26.15, $p < 0.001$	z =−28.24, $p < 0.001$
	[−0.651, −0.549]	[−0.557, −0.447]	[−0.618, −0.413]	[−0.612, −0.526]	[−0.624, −0.488]	[−0.634, −0.508]	[−0.623, −0.511]	[−0.579, −0.482]
Intercept	z =−15.76, $p < 0.001$	z =−18.62, $p < 0.001$	z =−30.77, $p < 0.001$	z =−6.54, $p < 0.001$	z =−25.51, $p < 0.001$	z =−24.77, $p < 0.001$	z =−20.06, $p < 0.001$	z =−12.66, $p < 0.001$
	[−1.501, −1.079]	[−1.730, −1.309]	[−3.581, −3.028]	[−0.644, −0.280]	[−2.471, −2.018]	[−2.454, −1.992]	[−1.831, −1.414]	[−1.109, −0.734]
Post-level RE	✓	✓	✓	✓	✓	✓	✓	✓
#Observations	25,312	25,312	25,312	25,312	25,312	25,312	25,312	25,312

Table S81: Regression results for two-period ATT estimations, based on economy-related posts and across reposter characteristics. Post-specific random effects are included. Reported are coefficient estimates with standard errors in parentheses. * $p < 0.01$, ** $p < 0.005$, *** $p < 0.001$. Exact z statistics, p values and 99% CIs are reported in Table S82.

	PriorInteraction		Verified		Political leaning		Misinformation exposure	
	(1)	(2)	(3)	(4)	(5)	(6)	(7)	(8)
	Yes	No	Yes	No	Left	Right	High	Low
Display	0.586**	1.139***	0.454	0.900***	0.851***	0.963***	0.909***	0.862***
	(0.207)	(0.193)	(0.221)	(0.179)	(0.212)	(0.216)	(0.194)	(0.185)
After	−0.156	−0.092	−0.255	−0.112	−0.127	−0.290**	−0.226**	−0.060
	(0.065)	(0.076)	(0.126)	(0.058)	(0.081)	(0.089)	(0.076)	(0.064)
Display × After	−0.860***	−0.997***	−0.631***	−0.928***	−0.788***	−0.853***	−0.818***	−0.961***
	(0.067)	(0.078)	(0.136)	(0.059)	(0.083)	(0.092)	(0.078)	(0.065)
PostAge	−0.626***	−0.502***	−0.552***	−0.589***	−0.624***	−0.469***	−0.523***	−0.600***
	(0.040)	(0.045)	(0.078)	(0.035)	(0.050)	(0.053)	(0.046)	(0.039)
Intercept	−1.224***	−1.539***	−3.021***	−0.526***	−2.125***	−2.002***	−1.454***	−1.064***
	(0.154)	(0.148)	(0.192)	(0.133)	(0.165)	(0.170)	(0.149)	(0.139)
Post-level RE	✓	✓	✓	✓	✓	✓	✓	✓
#Observations	6,320	6,320	6,320	6,320	6,320	6,320	6,320	6,320

Table S82: Details of z statistics, p values and 99% CIs (in brackets) for the coefficient estimates reported in Table S81.

	PriorInteraction		Verified		Political leaning		Misinformation exposure	
	(1)	(2)	(3)	(4)	(5)	(6)	(7)	(8)
	Yes	No	Yes	No	Left	Right	High	Low
Display	z =2.83, p =0.005	z =5.91, $p < 0.001$	z =2.06, p =0.040	z =5.04, $p < 0.001$	z =4.01, $p < 0.001$	z =4.45, $p < 0.001$	z =4.68, $p < 0.001$	z =4.65, $p < 0.001$
	[0.053, 1.119]	[0.642, 1.636]	[−0.115, 1.023]	[0.440, 1.360]	[0.305, 1.397]	[0.406, 1.520]	[0.409, 1.409]	[0.384, 1.340]
After	z =−2.40, p =0.016	z =−1.21, p =0.225	z =−2.02, p =0.043	z =−1.95, p =0.051	z =−1.56, p =0.120	z =−3.27, p =0.001	z =−2.99, p =0.003	z =−0.95, p =0.344
	[−0.323, 0.011]	[−0.288, 0.104]	[−0.581, 0.070]	[−0.261, 0.036]	[−0.336, 0.083]	[−0.518, −0.062]	[−0.421, −0.031]	[−0.224, 0.104]
Display×After	z =−12.80, $p < 0.001$	z =−12.84, $p < 0.001$	z =−4.63, $p < 0.001$	z =−15.62, $p < 0.001$	z =−9.47, $p < 0.001$	z =−9.24, $p < 0.001$	z =−10.47, $p < 0.001$	z =−14.68, $p < 0.001$
	[−1.033, −0.687]	[−1.197, −0.797]	[−0.981, −0.280]	[−1.081, −0.775]	[−1.003, −0.574]	[−1.091, −0.616]	[−1.019, −0.617]	[−1.130, −0.793]
PostAge	z =−15.47, $p < 0.001$	z =−11.14, $p < 0.001$	z =−7.09, $p < 0.001$	z =−16.96, $p < 0.001$	z =−12.45, $p < 0.001$	z =−8.80, $p < 0.001$	z =−11.45, $p < 0.001$	z =−15.38, $p < 0.001$
	[−0.730, −0.522]	[−0.617, −0.386]	[−0.752, −0.352]	[−0.679, −0.500]	[−0.753, −0.495]	[−0.606, −0.332]	[−0.640, −0.405]	[−0.700, −0.499]
Intercept	z =−7.93, $p < 0.001$	z =−10.37, $p < 0.001$	z =−15.75, $p < 0.001$	z =−3.97, $p < 0.001$	z =−12.85, $p < 0.001$	z =−11.75, $p < 0.001$	z =−9.74, $p < 0.001$	z =−7.65, $p < 0.001$
	[−1.622, −0.827]	[−1.921, −1.157]	[−3.515, −2.527]	[−0.868, −0.185]	[−2.551, −1.699]	[−2.441, −1.563]	[−1.839, −1.070]	[−1.422, −0.705]
Post-level RE	✓	✓	✓	✓	✓	✓	✓	✓
#Observations	6,320	6,320	6,320	6,320	6,320	6,320	6,320	6,320

Table S83: Regression results for two-period ATT estimations, based on non-economy-related posts and across reposter characteristics. Post-specific random effects are included. Reported are coefficient estimates with standard errors in parentheses. * $p < 0.01$, ** $p < 0.005$, *** $p < 0.001$. Exact z statistics, p values and 99% CIs are reported in Table S84.

	PriorInteraction		Verified		Political leaning		Misinformation exposure	
	(1)	(2)	(3)	(4)	(5)	(6)	(7)	(8)
	Yes	No	Yes	No	Left	Right	High	Low
Display	0.295***	0.818***	0.459***	0.594***	0.428***	0.706***	0.651***	0.562***
	(0.084)	(0.081)	(0.088)	(0.073)	(0.084)	(0.085)	(0.079)	(0.075)
After	−0.030	0.090***	−0.095	0.012	−0.004	0.026	0.019	0.000
	(0.022)	(0.027)	(0.044)	(0.020)	(0.031)	(0.028)	(0.025)	(0.023)
Display × After	−0.882***	−1.162***	−0.937***	−1.014***	−1.007***	−0.998***	−1.022***	−0.991***
	(0.023)	(0.028)	(0.048)	(0.021)	(0.033)	(0.029)	(0.026)	(0.024)
PostAge	−0.632***	−0.502***	−0.571***	−0.581***	−0.548***	−0.591***	−0.583***	−0.541***
	(0.014)	(0.016)	(0.028)	(0.012)	(0.019)	(0.017)	(0.015)	(0.014)
Intercept	−0.950***	−1.231***	−3.007***	−0.127	−1.939***	−1.803***	−1.215***	−0.639***
	(0.063)	(0.062)	(0.076)	(0.054)	(0.066)	(0.066)	(0.060)	(0.056)
Post-level RE	✓	✓	✓	✓	✓	✓	✓	✓
#Observations	44,288	44,288	44,288	44,288	44,288	44,288	44,288	44,288

Table S84: Details of z statistics, p values and 99% CIs (in brackets) for the coefficient estimates reported in Table S83.

	PriorInteraction		Verified		Political leaning		Misinformation exposure	
	(1)	(2)	(3)	(4)	(5)	(6)	(7)	(8)
	Yes	No	Yes	No	Left	Right	High	Low
Display	z =3.53, $p < 0.001$	z =10.07, $p < 0.001$	z =5.20, $p < 0.001$	z =8.19, $p < 0.001$	z =5.11, $p < 0.001$	z =8.29, $p < 0.001$	z =8.22, $p < 0.001$	z =7.54, $p < 0.001$
	[0.080, 0.510]	[0.609, 1.027]	[0.232, 0.687]	[0.407, 0.781]	[0.212, 0.644]	[0.487, 0.926]	[0.447, 0.855]	[0.370, 0.754]
After	z =−1.39, p =0.165	z =3.36, $p < 0.001$	z =−2.15, p =0.032	z =0.60, p =0.552	z =−0.14, p =0.891	z =0.93, p =0.354	z =0.78, p =0.438	z =0.00, p =0.999
	[−0.087, 0.026]	[0.021, 0.158]	[−0.210, 0.019]	[−0.040, 0.064]	[−0.084, 0.075]	[−0.046, 0.098]	[−0.045, 0.083]	[−0.058, 0.058]
Display×After	z =−37.93, $p < 0.001$	z =−41.77, $p < 0.001$	z =−19.54, $p < 0.001$	z =−47.73, $p < 0.001$	z =−30.74, $p < 0.001$	z =−34.23, $p < 0.001$	z =−39.36, $p < 0.001$	z =−41.71, $p < 0.001$
	[−0.941, −0.822]	[−1.234, −1.091]	[−1.060, −0.813]	[−1.069, −0.959]	[−1.091, −0.922]	[−1.073, −0.923]	[−1.089, −0.955]	[−1.052, −0.930]
PostAge	z =−45.75, $p < 0.001$	z =−31.76, $p < 0.001$	z =−20.48, $p < 0.001$	z =−47.66, $p < 0.001$	z =−28.37, $p < 0.001$	z =−34.43, $p < 0.001$	z =−38.13, $p < 0.001$	z =−39.41, $p < 0.001$
	[−0.668, −0.596]	[−0.542, −0.461]	[−0.643, −0.499]	[−0.612, −0.549]	[−0.597, −0.498]	[−0.636, −0.547]	[−0.622, −0.543]	[−0.576, −0.506]
Intercept	z =−15.15, $p < 0.001$	z =−19.87, $p < 0.001$	z =−39.56, $p < 0.001$	z =−2.33, p =0.020	z =−29.49, $p < 0.001$	z =−27.32, $p < 0.001$	z =−20.13, $p < 0.001$	z =−11.38, $p < 0.001$
	[−1.111, −0.788]	[−1.390, −1.071]	[−3.202, −2.811]	[−0.266, 0.013]	[−2.108, −1.769]	[−1.973, −1.633]	[−1.370, −1.059]	[−0.784, −0.495]
Post-level RE	✓	✓	✓	✓	✓	✓	✓	✓
#Observations	44,288	44,288	44,288	44,288	44,288	44,288	44,288	44,288

Table S85: Regression results for two-period ATT estimations, based on health-related posts and across reposter characteristics. Post-specific random effects are included. Reported are coefficient estimates with standard errors in parentheses. $^{*}\ p < 0.01$, $^{**}\ p < 0.005$, $^{***}\ p < 0.001$. Exact z statistics, p values and 99% CIs are reported in Table S86.

	PriorInteraction		Verified		Political leaning		Misinformation exposure	
	(1)	(2)	(3)	(4)	(5)	(6)	(7)	(8)
	Yes	No	Yes	No	Left	Right	High	Low
Display	−0.046	0.465	−0.130	0.235	0.074	0.222	0.174	0.316
	(0.213)	(0.239)	(0.221)	(0.197)	(0.246)	(0.223)	(0.212)	(0.203)
After	−0.052	0.148	−0.124	0.014	−0.011	0.066	0.035	−0.036
	(0.052)	(0.069)	(0.095)	(0.050)	(0.093)	(0.060)	(0.057)	(0.061)
Display × After	-0.726^{***}	-1.043^{***}	-0.722^{***}	-0.856^{***}	-0.921^{***}	-0.872^{***}	-0.862^{***}	-0.822^{***}
	(0.057)	(0.076)	(0.110)	(0.055)	(0.106)	(0.066)	(0.062)	(0.067)
PostAge	-0.594^{***}	-0.499^{***}	-0.507^{***}	-0.554^{***}	-0.565^{***}	-0.598^{***}	-0.598^{***}	-0.467^{***}
	(0.034)	(0.043)	(0.063)	(0.032)	(0.061)	(0.039)	(0.036)	(0.039)
Intercept	-0.451^{**}	-1.182^{***}	-2.106^{***}	0.168	-2.197^{***}	-0.802^{***}	-0.459^{**}	-0.679^{***}
	(0.152)	(0.174)	(0.171)	(0.141)	(0.188)	(0.161)	(0.152)	(0.148)
Post-level RE	✓	✓	✓	✓	✓	✓	✓	✓
#Observations	5,616	5,616	5,616	5,616	5,616	5,616	5,616	5,616

Table S86: Details of z statistics, p values and 99% CIs (in brackets) for the coefficient estimates reported in Table S85.

	PriorInteraction		Verified		Political leaning		Misinformation exposure	
	(1)	(2)	(3)	(4)	(5)	(6)	(7)	(8)
	Yes	No	Yes	No	Left	Right	High	Low
Display	z =−0.21, p =0.831	z =1.95, p =0.051	z =−0.59, p =0.557	z =1.19, p =0.233	z =0.30, p =0.762	z =0.99, p =0.320	z =0.82, p =0.411	z =1.55, p =0.121
	[−0.594, 0.503]	[−0.150, 1.079]	[−0.699, 0.439]	[−0.272, 0.742]	[−0.559, 0.707]	[−0.353, 0.797]	[−0.371, 0.719]	[−0.209, 0.840]
After	z =−1.00, p =0.317	z =2.13, p =0.033	z =−1.30, p =0.193	z =0.27, p =0.786	z =−0.12, p =0.904	z =1.10, p =0.272	z =0.63, p =0.532	z =−0.58, p =0.561
	[−0.185, 0.081]	[−0.031, 0.327]	[−0.368, 0.121]	[−0.116, 0.143]	[−0.251, 0.228]	[−0.089, 0.222]	[−0.110, 0.181]	[−0.194, 0.122]
Display×After	z =−12.70, $p < 0.001$	z =−13.70, $p < 0.001$	z =−6.56, $p < 0.001$	z =−15.63, $p < 0.001$	z =−8.67, $p < 0.001$	z =−13.28, $p < 0.001$	z =−13.88, $p < 0.001$	z =−12.29, $p < 0.001$
	[−0.874, −0.579]	[−1.239, −0.847]	[−1.006, −0.439]	[−0.997, −0.715]	[−1.194, −0.647]	[−1.042, −0.703]	[−1.022, −0.702]	[−0.994, −0.649]
PostAge	z =−17.51, $p < 0.001$	z =−11.49, $p < 0.001$	z =−7.99, $p < 0.001$	z =−17.55, $p < 0.001$	z =−9.22, $p < 0.001$	z =−15.46, $p < 0.001$	z =−16.44, $p < 0.001$	z =−12.00, $p < 0.001$
	[−0.681, −0.507]	[−0.611, −0.387]	[−0.670, −0.343]	[−0.635, −0.472]	[−0.722, −0.407]	[−0.697, −0.498]	[−0.692, −0.505]	[−0.567, −0.367]
Intercept	z =−2.97, p =0.003	z =−6.81, $p < 0.001$	z =−12.34, $p < 0.001$	z =1.19, p =0.232	z =−11.69, $p < 0.001$	z =−4.98, $p < 0.001$	z =−3.02, p =0.003	z =−4.59, $p < 0.001$
	[−0.843, −0.059]	[−1.629, −0.734]	[−2.545, −1.666]	[−0.194, 0.530]	[−2.681, −1.713]	[−1.216, −0.387]	[−0.850, −0.067]	[−1.060, −0.298]
Post-level RE	✓	✓	✓	✓	✓	✓	✓	✓
#Observations	5,616	5,616	5,616	5,616	5,616	5,616	5,616	5,616

Table S87: Regression results for two-period ATT estimations, based on non-health-related posts and across reposter characteristics. Post-specific random effects are included. Reported are coefficient estimates with standard errors in parentheses. $^{*}\ p < 0.01$, $^{**}\ p < 0.005$, $^{***}\ p < 0.001$. Exact z statistics, p values and 99% CIs are reported in Table S88.

	PriorInteraction		Verified		Political leaning		Misinformation exposure	
	(1)	(2)	(3)	(4)	(5)	(6)	(7)	(8)
	Yes	No	Yes	No	Left	Right	High	Low
Display	0.392***	0.911***	0.565***	0.690***	0.522***	0.835***	0.771***	0.633***
	(0.083)	(0.079)	(0.088)	(0.072)	(0.082)	(0.084)	(0.078)	(0.074)
After	−0.042	0.059	−0.113	−0.004	−0.017	−0.023	−0.014	−0.003
	(0.023)	(0.027)	(0.047)	(0.021)	(0.030)	(0.030)	(0.026)	(0.023)
Display × After	−0.900***	−1.151***	−0.928***	−1.022***	−0.985***	−0.999***	−1.020***	−1.004***
	(0.024)	(0.028)	(0.050)	(0.022)	(0.032)	(0.031)	(0.027)	(0.024)
PostAge	−0.638***	−0.503***	−0.579***	−0.586***	−0.557***	−0.575***	−0.572***	−0.557***
	(0.014)	(0.016)	(0.029)	(0.012)	(0.019)	(0.018)	(0.016)	(0.014)
Intercept	−1.066***	−1.288***	−3.146***	−0.228***	−1.933***	−1.979***	−1.361***	−0.696***
	(0.063)	(0.061)	(0.077)	(0.054)	(0.065)	(0.066)	(0.060)	(0.056)
Post-level RE	✓	✓	✓	✓	✓	✓	✓	✓
#Observations	44,992	44,992	44,992	44,992	44,992	44,992	44,992	44,992

Table S88: Details of z statistics, p values and 99% CIs (in brackets) for the coefficient estimates reported in Table S87.

	PriorInteraction		Verified		Political leaning		Misinformation exposure	
	(1)	(2)	(3)	(4)	(5)	(6)	(7)	(8)
	Yes	No	Yes	No	Left	Right	High	Low
Display	z =4.71, $p < 0.001$	z =11.51, $p < 0.001$	z =6.40, $p < 0.001$	z =9.61, $p < 0.001$	z =6.34, $p < 0.001$	z =9.89, $p < 0.001$	z =9.87, $p < 0.001$	z =8.59, $p < 0.001$
	[0.178, 0.606]	[0.707, 1.115]	[0.338, 0.793]	[0.505, 0.875]	[0.310, 0.734]	[0.617, 1.053]	[0.570, 0.972]	[0.443, 0.823]
After	z =−1.84, p =0.066	z =2.20, p =0.028	z =−2.43, p =0.015	z =−0.19, p =0.853	z =−0.56, p =0.574	z =−0.77, p =0.444	z =−0.53, p =0.599	z =−0.15, p =0.879
	[−0.100, 0.017]	[−0.010, 0.129]	[−0.233, 0.007]	[−0.057, 0.049]	[−0.095, 0.061]	[−0.099, 0.054]	[−0.080, 0.053]	[−0.062, 0.055]
Display×After	z =−37.85, $p < 0.001$	z =−41.15, $p < 0.001$	z =−18.64, $p < 0.001$	z =−47.47, $p < 0.001$	z =−30.88, $p < 0.001$	z =−32.47, $p < 0.001$	z =−37.93, $p < 0.001$	z =−42.27, $p < 0.001$
	[−0.962, −0.839]	[−1.223, −1.079]	[−1.056, −0.800]	[−1.077, −0.966]	[−1.068, −0.903]	[−1.078, −0.919]	[−1.090, −0.951]	[−1.066, −0.943]
PostAge	z =−45.10, $p < 0.001$	z =−31.71, $p < 0.001$	z =−20.12, $p < 0.001$	z =−47.49, $p < 0.001$	z =−29.53, $p < 0.001$	z =−31.87, $p < 0.001$	z =−36.23, $p < 0.001$	z =−40.55, $p < 0.001$
	[−0.674, −0.601]	[−0.544, −0.462]	[−0.653, −0.505]	[−0.618, −0.554]	[−0.605, −0.508]	[−0.621, −0.528]	[−0.613, −0.532]	[−0.592, −0.521]
Intercept	z =−16.99, $p < 0.001$	z =−21.23, $p < 0.001$	z =−40.72, $p < 0.001$	z =−4.24, $p < 0.001$	z =−29.92, $p < 0.001$	z =−29.85, $p < 0.001$	z =−22.67, $p < 0.001$	z =−12.49, $p < 0.001$
	[−1.227, −0.904]	[−1.444, −1.132]	[−3.345, −2.947]	[−0.367, −0.090]	[−2.099, −1.767]	[−2.150, −1.808]	[−1.516, −1.206]	[−0.840, −0.553]
Post-level RE	✓	✓	✓	✓	✓	✓	✓	✓
#Observations	44,992	44,992	44,992	44,992	44,992	44,992	44,992	44,992

Table S89: Regression results for two-period ATT estimations, based on political posts and across reposter characteristics. Post-specific random effects are included. Reported are coefficient estimates with standard errors in parentheses. $^{*}\ p < 0.01$, $^{**}\ p < 0.005$, $^{***}\ p < 0.001$. Exact z statistics, p values and 99% CIs are reported in Table S90.

	PriorInteraction		Verified		Political leaning		Misinformation exposure	
	(1)	(2)	(3)	(4)	(5)	(6)	(7)	(8)
	Yes	No	Yes	No	Left	Right	High	Low
Display	0.334	0.773***	0.327	0.572***	0.380	0.735***	0.651***	0.556***
	(0.134)	(0.139)	(0.145)	(0.123)	(0.151)	(0.147)	(0.131)	(0.129)
After	−0.078	−0.012	−0.108	−0.059	−0.051	−0.085	−0.049	−0.061
	(0.037)	(0.048)	(0.070)	(0.035)	(0.053)	(0.047)	(0.042)	(0.041)
Display × After	−0.870***	−1.015***	−0.920***	−0.926***	−0.868***	−0.939***	−0.949***	−0.909***
	(0.039)	(0.049)	(0.075)	(0.036)	(0.055)	(0.049)	(0.043)	(0.042)
PostAge	−0.659***	−0.536***	−0.636***	−0.615***	−0.625***	−0.601***	−0.616***	−0.585***
	(0.023)	(0.028)	(0.045)	(0.021)	(0.033)	(0.029)	(0.026)	(0.025)
Intercept	−0.587***	−1.363***	−2.574***	−0.100	−2.026***	−1.500***	−0.931***	−0.800***
	(0.102)	(0.108)	(0.122)	(0.093)	(0.119)	(0.114)	(0.101)	(0.098)
Post-level RE	✓	✓	✓	✓	✓	✓	✓	✓
#Observations	14,288	14,288	14,288	14,288	14,288	14,288	14,288	14,288

Table S90: Details of z statistics, p values and 99% CIs (in brackets) for the coefficient estimates reported in Table S89.

	PriorInteraction		Verified		Political leaning		Misinformation exposure	
	(1)	(2)	(3)	(4)	(5)	(6)	(7)	(8)
	Yes	No	Yes	No	Left	Right	High	Low
Display	z =2.49, p =0.013	z =5.57, $p < 0.001$	z =2.25, p =0.024	z =4.65, $p < 0.001$	z =2.52, p =0.012	z =5.00, $p < 0.001$	z =4.96, $p < 0.001$	z =4.32, $p < 0.001$
	[−0.012, 0.680]	[0.415, 1.131]	[−0.047, 0.700]	[0.255, 0.889]	[−0.009, 0.769]	[0.357, 1.114]	[0.312, 0.989]	[0.225, 0.887]
After	z =−2.07, p =0.038	z =−0.25, p =0.804	z =−1.55, p =0.121	z =−1.69, p =0.092	z =−0.96, p =0.337	z =−1.81, p =0.070	z =−1.17, p =0.243	z =−1.51, p =0.130
	[−0.174, 0.019]	[−0.135, 0.111]	[−0.288, 0.072]	[−0.149, 0.031]	[−0.189, 0.086]	[−0.207, 0.036]	[−0.156, 0.059]	[−0.166, 0.043]
Display×After	z =−22.21, $p < 0.001$	z =−20.90, $p < 0.001$	z =−12.27, $p < 0.001$	z =−25.60, $p < 0.001$	z =−15.74, $p < 0.001$	z =−19.25, $p < 0.001$	z =−21.97, $p < 0.001$	z =−21.81, $p < 0.001$
	[−0.971, −0.769]	[−1.141, −0.890]	[−1.113, −0.726]	[−1.019, −0.832]	[−1.010, −0.726]	[−1.065, −0.813]	[−1.060, −0.838]	[−1.016, −0.801]
PostAge	z =−28.12, $p < 0.001$	z =−18.80, $p < 0.001$	z =−14.26, $p < 0.001$	z =−29.13, $p < 0.001$	z =−18.67, $p < 0.001$	z =−20.92, $p < 0.001$	z =−24.09, $p < 0.001$	z =−23.50, $p < 0.001$
	[−0.719, −0.598]	[−0.609, −0.462]	[−0.751, −0.521]	[−0.670, −0.561]	[−0.711, −0.539]	[−0.675, −0.527]	[−0.682, −0.550]	[−0.649, −0.521]
Intercept	z =−5.77, $p < 0.001$	z =−12.62, $p < 0.001$	z =−21.16, $p < 0.001$	z =−1.07, p =0.283	z =−16.99, $p < 0.001$	z =−13.16, $p < 0.001$	z =−9.24, $p < 0.001$	z =−8.13, $p < 0.001$
	[−0.849, −0.325]	[−1.641, −1.085]	[−2.887, −2.261]	[−0.340, 0.140]	[−2.333, −1.719]	[−1.794, −1.206]	[−1.191, −0.671]	[−1.054, −0.547]
Post-level RE	✓	✓	✓	✓	✓	✓	✓	✓
#Observations	14,288	14,288	14,288	14,288	14,288	14,288	14,288	14,288

Table S91: Regression results for two-period ATT estimations, based on non-political posts and across reposter characteristics. Post-specific random effects are included. Reported are coefficient estimates with standard errors in parentheses. * $p < 0.01$, ** $p < 0.005$, *** $p < 0.001$. Exact z statistics, p values and 99% CIs are reported in Table S92.

	PriorInteraction		Verified		Political leaning		Misinformation exposure	
	(1)	(2)	(3)	(4)	(5)	(6)	(7)	(8)
	Yes	No	Yes	No	Left	Right	High	Low
Display	0.322***	0.902***	0.512***	0.661***	0.525***	0.737***	0.696***	0.624***
	(0.094)	(0.089)	(0.099)	(0.081)	(0.091)	(0.094)	(0.088)	(0.082)
After	−0.028	0.103***	−0.116	0.023	−0.004	0.033	0.014	0.015
	(0.025)	(0.030)	(0.052)	(0.023)	(0.034)	(0.032)	(0.029)	(0.025)
Display × After	−0.884***	−1.194***	−0.896***	−1.038***	−1.028***	−1.005***	−1.025***	−1.019***
	(0.026)	(0.031)	(0.057)	(0.024)	(0.037)	(0.034)	(0.030)	(0.026)
PostAge	−0.617***	−0.492***	−0.529***	−0.568***	−0.530***	−0.565***	−0.557***	−0.535***
	(0.016)	(0.017)	(0.032)	(0.014)	(0.021)	(0.020)	(0.018)	(0.015)
Intercept	−1.154***	−1.243***	−3.183***	−0.214***	−1.942***	−1.968***	−1.379***	−0.658***
	(0.070)	(0.068)	(0.087)	(0.060)	(0.071)	(0.073)	(0.067)	(0.061)
Post-level RE	✓	✓	✓	✓	✓	✓	✓	✓
#Observations	36,320	36,320	36,320	36,320	36,320	36,320	36,320	36,320

Table S92: Details of z statistics, p values and 99% CIs (in brackets) for the coefficient estimates reported in Table S91.

	PriorInteraction		Verified		Political leaning		Misinformation exposure	
	(1)	(2)	(3)	(4)	(5)	(6)	(7)	(8)
	Yes	No	Yes	No	Left	Right	High	Low
Display	z =3.43, $p < 0.001$	z =10.12, $p < 0.001$	z =5.15, $p < 0.001$	z =8.21, $p < 0.001$	z =5.76, $p < 0.001$	z =7.84, $p < 0.001$	z =7.87, $p < 0.001$	z =7.59, $p < 0.001$
	[0.080, 0.565]	[0.672, 1.132]	[0.256, 0.767]	[0.454, 0.869]	[0.290, 0.759]	[0.495, 0.980]	[0.468, 0.923]	[0.412, 0.836]
After	z =−1.12, p =0.263	z =3.50, $p < 0.001$	z =−2.21, p =0.027	z =1.03, p =0.304	z =−0.12, p =0.908	z =1.00, p =0.315	z =0.48, p =0.629	z =0.62, p =0.536
	[−0.092, 0.036]	[0.027, 0.180]	[−0.251, 0.019]	[−0.035, 0.082]	[−0.092, 0.084]	[−0.051, 0.116]	[−0.060, 0.087]	[−0.049, 0.080]
Display×After	z =−33.38, $p < 0.001$	z =−38.49, $p < 0.001$	z =−15.81, $p < 0.001$	z =−43.27, $p < 0.001$	z =−28.12, $p < 0.001$	z =−29.69, $p < 0.001$	z =−34.17, $p < 0.001$	z =−38.53, $p < 0.001$
	[−0.952, −0.816]	[−1.274, −1.114]	[−1.042, −0.750]	[−1.100, −0.976]	[−1.122, −0.933]	[−1.092, −0.918]	[−1.102, −0.948]	[−1.087, −0.951]
PostAge	z =−39.23, $p < 0.001$	z =−28.12, $p < 0.001$	z =−16.28, $p < 0.001$	z =−41.48, $p < 0.001$	z =−24.83, $p < 0.001$	z =−28.45, $p < 0.001$	z =−31.65, $p < 0.001$	z =−35.26, $p < 0.001$
	[−0.657, −0.576]	[−0.537, −0.447]	[−0.612, −0.445]	[−0.603, −0.533]	[−0.585, −0.475]	[−0.617, −0.514]	[−0.602, −0.511]	[−0.574, −0.496]
Intercept	z =−16.40, $p < 0.001$	z =−18.42, $p < 0.001$	z =−36.72, $p < 0.001$	z =−3.57, $p < 0.001$	z =−27.33, $p < 0.001$	z =−26.95, $p < 0.001$	z =−20.53, $p < 0.001$	z =−10.70, $p < 0.001$
	[−1.335, −0.973]	[−1.417, −1.069]	[−3.407, −2.960]	[−0.368, −0.060]	[−2.125, −1.759]	[−2.156, −1.780]	[−1.552, −1.206]	[−0.816, −0.499]
Post-level RE	✓	✓	✓	✓	✓	✓	✓	✓
#Observations	36,320	36,320	36,320	36,320	36,320	36,320	36,320	36,320

Table S93: Regression results for two-period ATT estimations, based on science-related posts and across reposter characteristics. Post-specific random effects are included. Reported are coefficient estimates with standard errors in parentheses. * $p < 0.01$, ** $p < 0.005$, *** $p < 0.001$. Exact z statistics, p values and 99% CIs are reported in Table S94.

	PriorInteraction		Verified		Political leaning		Misinformation exposure	
	(1)	(2)	(3)	(4)	(5)	(6)	(7)	(8)
	Yes	No	Yes	No	Left	Right	High	Low
Display	0.424	0.899***	0.407	0.739***	0.619**	0.791***	0.795***	0.698***
	(0.211)	(0.207)	(0.218)	(0.186)	(0.217)	(0.213)	(0.204)	(0.188)
After	0.085	0.228**	−0.182	0.196***	0.038	0.230**	0.182*	0.154
	(0.063)	(0.072)	(0.126)	(0.055)	(0.092)	(0.076)	(0.069)	(0.063)
Display × After	−0.973***	−1.246***	−1.029***	−1.099***	−1.076***	−1.154***	−1.113***	−1.077***
	(0.066)	(0.076)	(0.139)	(0.058)	(0.099)	(0.079)	(0.072)	(0.067)
PostAge	−0.639***	−0.516***	−0.473***	−0.607***	−0.532***	−0.587***	−0.592***	−0.563***
	(0.040)	(0.043)	(0.079)	(0.034)	(0.058)	(0.047)	(0.043)	(0.039)
Intercept	−1.616***	−1.638***	−3.020***	−0.740***	−2.429***	−2.190***	−1.781***	−1.183***
	(0.157)	(0.155)	(0.186)	(0.136)	(0.171)	(0.164)	(0.154)	(0.140)
Post-level RE	✓	✓	✓	✓	✓	✓	✓	✓
#Observations	6,480	6,480	6,480	6,480	6,480	6,480	6,480	6,480

Table S94: Details of z statistics, p values and 99% CIs (in brackets) for the coefficient estimates reported in Table S93.

	PriorInteraction		Verified		Political leaning		Misinformation exposure	
	(1)	(2)	(3)	(4)	(5)	(6)	(7)	(8)
	Yes	No	Yes	No	Left	Right	High	Low
Display	z =2.01, p =0.045	z =4.34, $p < 0.001$	z =1.87, p =0.062	z =3.99, $p < 0.001$	z =2.85, p =0.004	z =3.72, $p < 0.001$	z =3.90, $p < 0.001$	z =3.71, $p < 0.001$
	[−0.120, 0.969]	[0.366, 1.432]	[−0.154, 0.968]	[0.262, 1.217]	[0.060, 1.177]	[0.244, 1.339]	[0.270, 1.320]	[0.213, 1.183]
After	z =1.36, p =0.174	z =3.18, p =0.001	z =−1.45, p =0.148	z =3.54, $p < 0.001$	z =0.41, p =0.680	z =3.05, p =0.002	z =2.65, p =0.008	z =2.45, p =0.014
	[−0.076, 0.247]	[0.043, 0.412]	[−0.507, 0.142]	[0.053, 0.338]	[−0.199, 0.275]	[0.036, 0.425]	[0.005, 0.359]	[−0.008, 0.317]
Display×After	z =−14.82, $p < 0.001$	z =−16.45, $p < 0.001$	z =−7.40, $p < 0.001$	z =−18.86, $p < 0.001$	z =−10.90, $p < 0.001$	z =−14.55, $p < 0.001$	z =−15.45, $p < 0.001$	z =−16.16, $p < 0.001$
	[−1.142, −0.804]	[−1.442, −1.051]	[−1.388, −0.671]	[−1.249, −0.949]	[−1.330, −0.822]	[−1.359, −0.950]	[−1.299, −0.927]	[−1.249, −0.906]
PostAge	z =−15.82, $p < 0.001$	z =−11.96, $p < 0.001$	z =−6.02, $p < 0.001$	z =−17.91, $p < 0.001$	z =−9.22, $p < 0.001$	z =−12.51, $p < 0.001$	z =−13.85, $p < 0.001$	z =−14.52, $p < 0.001$
	[−0.743, −0.535]	[−0.627, −0.405]	[−0.676, −0.271]	[−0.695, −0.520]	[−0.681, −0.384]	[−0.708, −0.466]	[−0.703, −0.482]	[−0.663, −0.463]
Intercept	z =−10.27, $p < 0.001$	z =−10.58, $p < 0.001$	z =−16.26, $p < 0.001$	z =−5.45, $p < 0.001$	z =−14.17, $p < 0.001$	z =−13.39, $p < 0.001$	z =−11.55, $p < 0.001$	z =−8.48, $p < 0.001$
	[−2.021, −1.211]	[−2.036, −1.239]	[−3.499, −2.542]	[−1.090, −0.390]	[−2.870, −1.987]	[−2.612, −1.769]	[−2.178, −1.384]	[−1.542, −0.824]
Post-level RE	✓	✓	✓	✓	✓	✓	✓	✓
#Observations	6,480	6,480	6,480	6,480	6,480	6,480	6,480	6,480

Table S95: Regression results for two-period ATT estimations, based on non-science-related posts and across reposter characteristics. Post-specific random effects are included. Reported are coefficient estimates with standard errors in parentheses. * $p < 0.01$, ** $p < 0.005$, *** $p < 0.001$. Exact z statistics, p values and 99% CIs are reported in Table S96.

	PriorInteraction		Verified		Political leaning		Misinformation exposure	
	(1)	(2)	(3)	(4)	(5)	(6)	(7)	(8)
	Yes	No	Yes	No	Left	Right	High	Low
Display	0.314***	0.856***	0.469***	0.616***	0.460***	0.733***	0.668***	0.585***
	(0.083)	(0.081)	(0.088)	(0.072)	(0.083)	(0.085)	(0.079)	(0.074)
After	−0.057*	0.052	−0.104	−0.025	−0.024	−0.034	−0.027	−0.025
	(0.022)	(0.027)	(0.044)	(0.020)	(0.030)	(0.028)	(0.025)	(0.023)
Display × After	−0.871***	−1.131***	−0.891***	−0.993***	−0.971***	−0.962***	−0.988***	−0.978***
	(0.023)	(0.028)	(0.048)	(0.021)	(0.032)	(0.030)	(0.026)	(0.024)
PostAge	−0.630***	−0.500***	−0.580***	−0.578***	−0.559***	−0.578***	−0.574***	−0.545***
	(0.014)	(0.016)	(0.028)	(0.012)	(0.019)	(0.017)	(0.015)	(0.014)
Intercept	−0.892***	−1.221***	−3.005***	−0.098	−1.891***	−1.785***	−1.174***	−0.623***
	(0.062)	(0.062)	(0.076)	(0.054)	(0.065)	(0.066)	(0.060)	(0.056)
Post-level RE	✓	✓	✓	✓	✓	✓	✓	✓
#Observations	44,128	44,128	44,128	44,128	44,128	44,128	44,128	44,128

Table S96: Details of z statistics, p values and 99% CIs (in brackets) for the coefficient estimates reported in Table S95.

	PriorInteraction		Verified		Political leaning		Misinformation exposure	
	(1)	(2)	(3)	(4)	(5)	(6)	(7)	(8)
	Yes	No	Yes	No	Left	Right	High	Low
Display	z =3.78, p < 0.001	z =10.62, p < 0.001	z =5.30, p < 0.001	z =8.54, p < 0.001	z =5.51, p < 0.001	z =8.59, p < 0.001	z =8.50, p < 0.001	z =7.87, p < 0.001
	[0.100, 0.528]	[0.648, 1.063]	[0.241, 0.697]	[0.430, 0.802]	[0.245, 0.675]	[0.513, 0.953]	[0.466, 0.871]	[0.393, 0.777]
After	z =−2.61, p =0.009	z =1.92, p =0.055	z =−2.34, p =0.019	z =−1.24, p =0.216	z =−0.78, p =0.435	z =−1.19, p =0.233	z =−1.06, p =0.289	z =−1.13, p =0.260
	[−0.114, −0.001]	[−0.018, 0.121]	[−0.218, 0.011]	[−0.077, 0.027]	[−0.102, 0.054]	[−0.107, 0.039]	[−0.091, 0.038]	[−0.084, 0.033]
Display×After	z =−37.45, p < 0.001	z =−40.53, p < 0.001	z =−18.64, p < 0.001	z =−46.67, p < 0.001	z =−30.30, p < 0.001	z =−32.46, p < 0.001	z =−37.77, p < 0.001	z =−41.22, p < 0.001
	[−0.931, −0.811]	[−1.203, −1.059]	[−1.014, −0.767]	[−1.048, −0.939]	[−1.054, −0.889]	[−1.038, −0.886]	[−1.056, −0.921]	[−1.039, −0.917]
PostAge	z =−45.67, p < 0.001	z =−31.52, p < 0.001	z =−20.81, p < 0.001	z =−47.36, p < 0.001	z =−29.46, p < 0.001	z =−33.16, p < 0.001	z =−37.32, p < 0.001	z =−39.68, p < 0.001
	[−0.666, −0.595]	[−0.541, −0.459]	[−0.651, −0.508]	[−0.610, −0.547]	[−0.607, −0.510]	[−0.622, −0.533]	[−0.614, −0.535]	[−0.580, −0.509]
Intercept	z =−14.31, p < 0.001	z =−19.83, p < 0.001	z =−39.38, p < 0.001	z =−1.81, p =0.071	z =−28.93, p < 0.001	z =−26.91, p < 0.001	z =−19.55, p < 0.001	z =−11.10, p < 0.001
	[−1.053, −0.732]	[−1.380, −1.063]	[−3.202, −2.809]	[−0.237, 0.042]	[−2.060, −1.723]	[−1.956, −1.614]	[−1.329, −1.019]	[−0.767, −0.478]
Post-level RE	✓	✓	✓	✓	✓	✓	✓	✓
#Observations	44,128	44,128	44,128	44,128	44,128	44,128	44,128	44,128

Supplementary Note 10: Robustness Checks

10.1 ATT Estimation With Post-Level Fixed Effects

Table S97 reports regression results for two-period ATT estimations based on the dataset for main analysis (Column (1)) and the dataset for reposter analysis (Column (2)), respectively. Using data for main analysis, the ATT estimate was –0.567 (99% CI: [–0.571, –0.563]; z = –228.97, $p <$ 0.001), slightly smaller than the ATT estimate with post-level random effects (ATT: –0.612; 99% CI: [–0.617, –0.608]; z = –211.71, $p <$ 0.001). However, the ATT estimation with post-level fixed effects dropped 25,200 repost count observations across 1,575 posts due to all zero outcomes in the dataset for main analysis. Based on the dataset for reposer analysis where 464 repost observations across 29 posts were dropped due to all zero outcomes during fixed-effects model estimation, the ATT estimate was –0.613 (99% CI: [–0.629, –0.595]; z = –55.53, $p <$ 0.001), which was similar to the ATT estimates with post-level random effects based on the datasets for both main analysis and reposter analysis.

10.2 ATT Estimation With Zero-Inflated Negative Binomial Regression

We used zero-inflated negative binomial regression models to account for potential excess zeros with the increase of post age. Table S97 reports regression results for two-period ATT estimations based on the dataset for main analysis (Column (3)) and the dataset for reposter analysis (Column (4)), respectively. Based on the posts for main analysis, the ATT estimate was –0.584 (99% CI: [–0.604, –0.562]; z = –44.37, $p <$ 0.001), closely aligning with the main ATT estimate in the mixed negative binomial regression model (ATT: –0.612; 99% CI: [–0.617, –0.608]; z = –211.71, $p <$ 0.001). Based on the posts for reposter analysis, the ATT estimate was –0.556 (99% CI: [–0.622, –0.480]; z = –13.13, $p <$ 0.001), consistent with the ATT estimates in the mixed negative binomial regression models based on the datasets for both main analysis and reposter analysis.

In summary, our results are consistent and robust across different modeling approaches and datasets.

Table S97: Regression results for two-period ATT estimation with post-level fixed effects based on the dataset for main analysis (Column (1)) and the subset for reposter analysis (Column (2)). Regression results for two-period ATT estimation with zero-inflated negative binomial regressions based on the dataset for main analysis (Column (3)) and the subset for reposter analysis (Column (4)). Reported are coefficient estimates with standard errors in parentheses. * $p < 0.01$, ** $p < 0.005$, *** $p < 0.001$. Exact z statistics, p values and 99% CIs are reported in Table S98.

	Post-level fixed effects		Zero-inflated negative binomial	
	(1) Data for main analysis	(2) Data for reposter analysis	(3) Data for main analysis	(4) Data for reposter analysis
Display	0.303***	0.191***	0.373***	0.249***
	(0.006)	(0.035)	(0.028)	(0.072)
After	–0.158***	–0.101***	–0.517***	–0.465***
	(0.003)	(0.016)	(0.014)	(0.035)
Display$\times$After	–0.836***	–0.948***	–0.876***	–0.813***
	(0.004)	(0.017)	(0.020)	(0.062)
PostAge	–0.401***	–0.413***		
	(0.002)	(0.010)		
Intercept	1.106***	1.348***	4.291***	1.949***
	(0.005)	(0.027)	(0.023)	(0.058)
Inflate				
PostAge			2.121***	1.600***
			(0.023)	(0.091)
Intercept			–5.207***	–3.961***
			(0.059)	(0.226)
Post-level FE	✓	✓		
#Observations	629,200	50,144	654,400	50,608
#Posts	39,325	3,134	40,900	3,163

Table S98: Details of z statistics, p values and 99% CIs (in brackets) for the coefficient estimates reported in Table S97.

	Post-level fixed effects		Zero-inflated negative binomial	
	(1) Data for main analysis	(2) Data for reposter analysis	(3) Data for main analysis	(4) Data for reposter analysis
Display	z =51.40, $p < 0.001$ [0.288, 0.318]	z =5.50, $p < 0.001$ [0.101, 0.280]	z =13.29, $p < 0.001$ [0.301, 0.445]	z =3.44, $p < 0.001$ [0.063, 0.436]
After	z =−46.91, $p < 0.001$ [−0.166, −0.149]	z =−6.20, $p < 0.001$ [−0.143, −0.059]	z =−35.83, $p < 0.001$ [−0.554, −0.480]	z =−13.33, $p < 0.001$ [−0.555, −0.375]
Display×After	z =−228.97, $p < 0.001$ [−0.846, −0.827]	z =−55.53, $p < 0.001$ [−0.992, −0.904]	z =−44.37, $p < 0.001$ [−0.927, −0.825]	z =−13.13, $p < 0.001$ [−0.972, −0.653]
PostAge	z =−207.29, $p < 0.001$ [−0.406, −0.396]	z =−40.70, $p < 0.001$ [−0.440, −0.387]		
Intercept	z =236.96, $p < 0.001$ [1.094, 1.118]	z =50.22, $p < 0.001$ [1.279, 1.418]	z =190.31, $p < 0.001$ [4.233, 4.349]	z =33.65, $p < 0.001$ [1.800, 2.098]
Inflate				
PostAge			z =92.12, $p < 0.001$ [2.061, 2.180]	z =17.59, $p < 0.001$ [1.366, 1.834]
Intercept			z =−87.94, $p < 0.001$ [−5.359, −5.054]	z =−17.52, $p < 0.001$ [−4.544, −3.379]
Post-level FE	✓	✓		
#Observations	629,200	50,144	654,400	50,608
#Posts	39,325	3,134	40,900	3,163

10.3 Analysis With Restrictions of Note Scores and Pre-Display Engagement

To further ensure the robustness of our estimates, we conducted an additional robustness check restricting the sample based on note scores and pre-display engagement. Specifically, we applied two restrictions. First, we restricted our analysis to posts with Community Notes that fall within a narrow window around the display threshold. This restriction can help mitigate unobserved confounding factors related to note scores, though it significantly reduces the number of posts during estimation. For our analysis, we selected the window of note helpfulness scores between 0.35 and 0.45, where 0.40 served as the display threshold. Within this selection window, we found 5,352 posts in the treatment group, while only 971 posts were in the control group. Second, to balance the treatment and control groups and ensure that variations in post engagement following the treatment are not influenced by pre-existing disparities prior to the intervention, we matched each post within the control group with a corresponding post from the treatment group based on the pre-display repost activity. We retained matched pairs where the number of reposts during the pre-display period did not differ by more than 0.1%, which resulted in 813 pairs.

Within this restricted sample, we assessed the observed diffusion of treated posts in comparison to the counterfactual diffusion of their matched counterparts. Specifically, we estimated the treatment effect using two complementary approaches: a Difference-in-Differences (DiD) design, analogous to our main analysis, and a Regression Discontinuity Design (RDD) based on note helpfulness scores.

DiD estimation: The DiD estimation results based on the matched pairs are reported in Column (1) of Table S99. Figure S14a shows the estimates for the multi-period and two-period ATTs. The two-period ATT estimate was –0.595 (99% CI: [–0.617, –0.572]; $z = -42.14$, $p < 0.001$), which is very close to the two-period ATT estimate in the main analysis, reinforcing the robustness of our findings.

Regression Discontinuity Design (RDD): As an additional check, we used the note scores as the running variable to estimate the treatment effect of community notes based on a Regression Discontinuity Design (RDD). Specifically, we evaluated the changes in repost count within the paired posts over the note helpfulness scores between and after display. As shown in Figure S14b, the data shows a clear drop in after-display reposts (the total number of reposts during the 12-hour after-display period) after the cutoff point of 0.40, while the before-display reposts (the total number of reposts during the 4-hour before-display period) remained relatively stable.

We specified the following RDD model to estimate the reduction in reposts after the cutoff relative to before:

$$\begin{aligned} log(E(RepostCount|\boldsymbol{x_{it}})) =& \beta_0 + \beta_1 After_t + \beta_2 Helpful_i + \beta_3 After_t \times Helpful_i + \\ & \beta_4 NoteScore_i + \beta_5 PostAge_{it} + \mu_{post}, \end{aligned} \tag{2}$$

where $After_t$ is a binary variable indicating whether the reposts were created after the display of community notes ($= 1$) or not ($= 0$). Additionally, the binary variable $Helpful_i$ indicates whether the notes associated with the source posts were eligible to earn helpful status based on the cutoff point. Therefore, the coefficient estimate for the interaction term $After_t \times Helpful_i$, i.e., β_3 represents the estimated treatment effect of community notes using RDD.

Note that the helpfulness scores in our dataset were computed from publicly available log data [10] and may differ slightly from those in the production environment, as ratings are updated dynamically. Consequently, a small number of posts with scores below 0.40 appear in the treatment group, and some with scores above 0.40 appear in the control group. We excluded these posts (188 below-threshold treatment posts and 247 above-threshold control posts) before estimating the model.

The estimation results are reported in Column (2) of Table S99. The RDD estimate for the treatment effect was –0.616 (99% CI: [–0.640, –0.591]; $z = $ –38.13, $p < 0.001$), which is nearly identical to the estimated effect using DiD in the main analysis.

Together, these findings provide strong confirmatory evidence that our estimates are not driven by systematic differences in post characteristics. Instead, the observed decline in engagement is attributable to the presence of Community Notes rather than underlying differences between noted and non-noted posts.

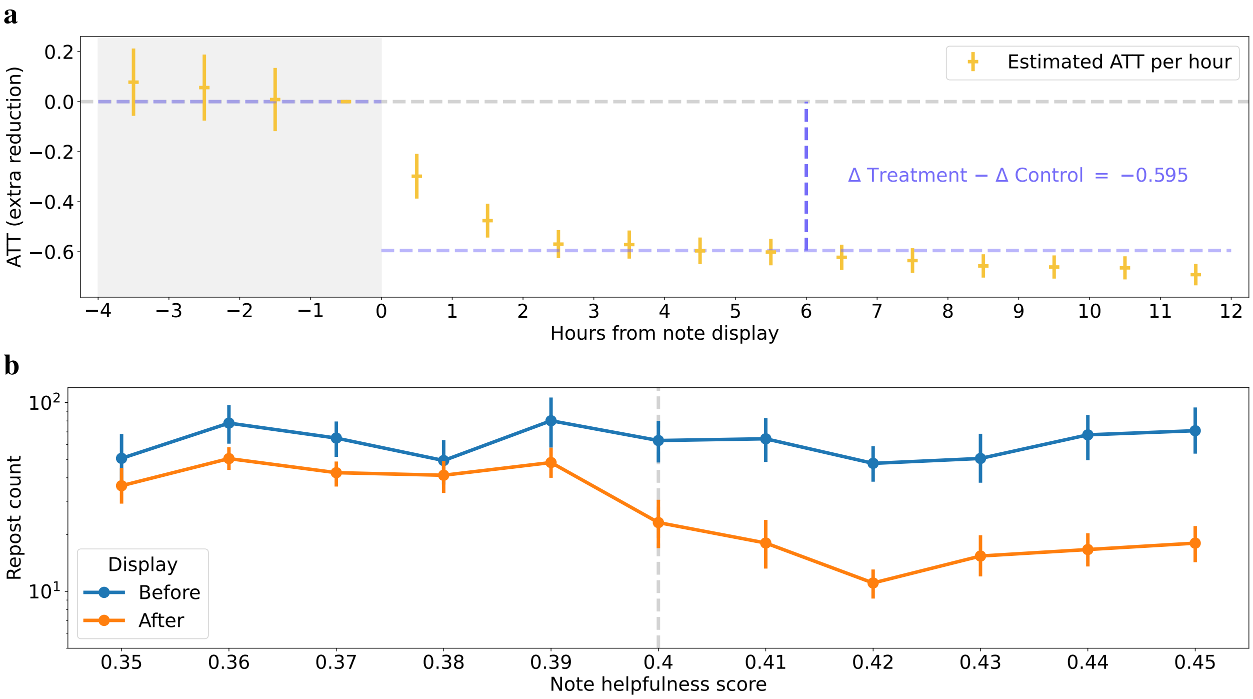

Figure S14: **Analysis with restrictions of note scores and pre-display engagement.** **(a)** Two-period (purple) and multi-period (yellow) ATTs estimated using DiD design. **(b)** Changes in repost counts before and after the display of Community Notes across note helpfulness scores. The before-display reposts represent the total number of reposts during the 4-hour before-display period, and the after-display reposts represent the total number of reposts during the 12-hour after-display period. The error bars represent 99% CIs.

10.4 Comparison to Previous Work Analyzing Aggregated Repost Counts

A previous study examined cumulative repost counts (i.e., aggregated engagement data rather than time-series data) for source posts created from the roll-out of Community Notes program until April 2023 and found no evidence showing that community notes reduced overall engagement with misleading posts [7]. One possible explanation is that the display of community notes might have been too slow to during the early stages of the program to produce a measurable effect on overall engagement. In contrast, our analysis – based on a longer observation timeframe until June 2024 – implies that community notes reduced the overall number of reposts for misleading posts on X (formerly Twitter) by 14.9%.

To reconcile these findings and assess consistency between the two studies, we repeated our analysis based on posts created at the same timeframe as in [7]. The DiD estimation results are reported in Column (3) of Table S99. The two-period ATT estimate was −0.491 (99% CI: [−0.510, −0.471]; $z = -45.95$, $p < 0.001$), indicating a weaker treatment effect during the early stages of the program. This result is consistent with our previous sensitivity analyses (see Supplementary Note 7), which showed that the treatment effect of community notes increased over time.

Next, we compared the ratio of the predicted cumulative repost count in the absence of displayed notes to the actual cumulative number of reposts. For posts created up to April 2023 (the period analyzed in [7]), the average reduction in reposts was only 7.2%, with half of the posts showing no more than a 1.5% decrease. This is substantially smaller than the reduction in total reposts observed during our full study period (mean of 14.9%; median of 10.1%). This can primarily be explained by two factors: (i) the post-level effect was smaller at this stage (49.1% vs. 61.2%), and (ii) the time-to-display was substantially longer (median of 27.8 hours vs. 18.1 hours), reducing the system-wide effect. The particularly small median reduction during the early stages of Community Notes program further indicates that the observed reduction was primarily driven by a few posts, rather than a broad reduction across all posts.

According to a two-sided KS-test, during the timeframe analyzed in [7], the observed repost

count for source posts with displayed community notes (mean = 2,792) was not significantly different from the predicted repost count in the absence of notes (mean = 3,146; $KS = 0.022, p = 0.310$; see Figure S15). Note that the lack of statistical significance is likely partially due to the smaller sample size (only 3,880 posts during this timeframe) and limited statistical power. However, the estimate aligns very closely with the 7.6% reduction reported in Chuai et al. (2025), which also did not reach statistical significance at common statistical significance thresholds.

Taken together, these findings demonstrate that both studies are consistent despite differences in methodology and timeframe: (i) in both timeframes, community notes had a substantial treatment effect at the individual post level, though the effect was smaller in the program's early stages. (ii) However, because time-to-display was significantly longer, the system-level impact was much smaller and not statistically significant in the early stage of the program. Thus, rather than being contradictory, the findings from [7] and this study offer complementary perspectives on how the system-level effect of community notes evolved as the feature improved.

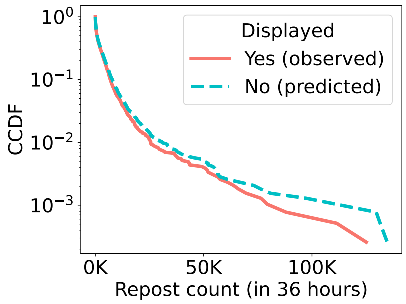

Figure S15: CCDFs showing the actually observed repost count for source posts with displayed community notes and the predicted repost count that the source posts would have received in the absence of community notes display. Only posts created from the roll-out of Community Notes program until April 2023 are included.

Table S99: Regression results for the treatment effect estimation with the restrictions on note scores and pre-display engagement (Column (1)–(2)) and the early stage of Community Notes program (Column (3)). Post-specific random effects are included. Reported are coefficient estimates with standard errors in parentheses. * $p < 0.01$, ** $p < 0.005$, *** $p < 0.001$. Exact z statistics, p values and 99% CIs are reported in Table S100.

	Restriction on note scores and pre-display engagement		Early stage of the program
	(1)	(2)	(3)
	DiD	RDD	DiD
Display	0.106		1.342***
	(0.102)		(0.089)
Helpful		–0.006	
		(0.257)	
After	0.225***	0.267***	0.162***
	(0.020)	(0.023)	(0.014)
Display×After	–0.905***		–0.675***
	(0.021)		(0.015)
Helpful×After		–0.958***	
		(0.025)	
NoteScore		2.372	
		(4.081)	
PostAge	–0.793***	–0.833***	–0.811***
	(0.012)	(0.014)	(0.008)
Intercept	2.213***	1.133	1.295***
	(0.073)	(1.510)	(0.063)
Post-level RE	✓	✓	✓
#Observations	26,016	19,056	49,184
#Posts	1,626	1,191	3,074

Table S100: Details of z statistics, p values and 99% CIs (in brackets) for the coefficient estimates reported in Table S99.

	Restriction on note scores and pre-display engagement		Early stage of the program
	(1)	(2)	(3)
	DiD	RDD	DiD
Display	z =1.04, p =0.296<br>[−0.156, 0.368]		z =15.00, $p < 0.001$<br>[1.111, 1.572]
Helpful		z =−0.02, p =0.982<br>[−0.669, 0.657]	
After	z =11.54, $p < 0.001$<br>[0.175, 0.275]	z =11.48, $p < 0.001$<br>[0.207, 0.327]	z =11.63, $p < 0.001$<br>[0.126, 0.198]
Display×After	z =−42.14, $p < 0.001$<br>[−0.960, −0.849]		z =−45.95, $p < 0.001$<br>[−0.713, −0.637]
Helpful×After		z =−38.13, $p < 0.001$<br>[−1.023, −0.893]	
NoteScore		z =0.58, p =0.561<br>[−8.141, 12.884]	
PostAge	z =−67.08, $p < 0.001$<br>[−0.823, −0.762]	z =−59.82, $p < 0.001$<br>[−0.869, −0.798]	z =−103.00, $p < 0.001$<br>[−0.832, −0.791]
Intercept	z =30.50, $p < 0.001$<br>[2.026, 2.400]	z =0.75, p =0.453<br>[−2.755, 5.022]	z =20.50, $p < 0.001$<br>[1.132, 1.457]
Post-level RE	✓	✓	✓
#Observations	26,016	19,056	49,184
#Posts	1,626	1,191	3,074

10.5 Alternative Before-Display Periods

In the main analysis, we omitted a small subset of community notes (5.2%) that were displayed within the initial four hours after post creation and used a four-hour before-display period for DiD estimation. This approach allowed us to incorporate sufficient pre-trend periods while minimizing the exclusion of early-displayed notes. However, we also found that community notes were more effective when displayed earlier. Given this, we conducted additional analyses based on shorter before-display periods to assess the extent to which this exclusion affected our estimates.

Table S101 reports the two-period DiD estimation results using a one-hour before-display period (Column (1)) and a two-hour before-display period (Column (2)). The ATT estimates were –0.629 (99% CI: [–0.636, –0.623]; $z = -143.43$, $p < 0.001$) based on the one-hour before-display period and –0.628 (99% CI: [–0.633, –0.623]; $z = -185.33$, $p < 0.001$) based on the two-hour before-display period. Compared to our main ATT estimate based on the four-hour pre-display period (ATT of –0.612), these values differed by only 0.017 and 0.016, respectively. These minimal differences imply that the exclusion of early-displayed notes had no meaningful effect on our estimates.

Table S101: Regression results for two-period ATT estimation with a before-display period of one hour (Column (1)) and a before-display period of two hours (Column (2)). Post-specific random effects are included. Reported are coefficient estimates with standard errors in parentheses. * $p < 0.01$, ** $p < 0.005$, *** $p < 0.001$. Exact z statistics, p values and 99% CIs are reported in Table S102.

	(1)<br>One-hour before-display period	(2)<br>Two-hour before-display period
Display	1.258***	1.246***
	(0.023)	(0.023)
After	0.066***	0.070***
	(0.005)	(0.004)
Display×After	–0.992***	–0.988***
	(0.007)	(0.005)
PostAge	–0.787***	–0.790***
	(0.002)	(0.002)
Intercept	1.193***	1.255***
	(0.017)	(0.016)
Post-level RE	✓	✓
#Observations	580,112	623,000
#Posts	44,624	44,500

Table S102: Details of z statistics, p values and 99% CIs (in brackets) for the coefficient estimates reported in Table S101.

	(1) One-hour before-display period	(2) Two-hour before-display period
Display	z =54.14, $p < 0.001$	z =54.78, $p < 0.001$
	[1.198, 1.317]	[1.188, 1.305]
After	z =11.96, $p < 0.001$	z =15.69, $p < 0.001$
	[0.052, 0.080]	[0.059, 0.082]
Display×After	z =−143.43, $p < 0.001$	z =−185.33, $p < 0.001$
	[−1.010, −0.974]	[−1.002, −0.974]
PostAge	z =−362.64, $p < 0.001$	z =−347.65, $p < 0.001$
	[−0.793, −0.782]	[−0.796, −0.784]
Intercept	z =72.09, $p < 0.001$	z =77.58, $p < 0.001$
	[1.150, 1.236]	[1.213, 1.296]
Post-level RE	✓	✓
#Observations	580,112	623,000
#Posts	44,624	44,500

10.6 Analysis With HonestDiD

To further validate the robustness of our findings, we quantified the aggregated average treatment effect on the treated (ATT) after the display of Community Notes using the HonestDiD method. Specifically, we performed a multi-period ATT estimation, treating the entire post-display period as a single interval, while the four hourly periods preceding the display were used to assess pre-trends within the HonestDiD framework.

The full estimation results are reported in Table S103, where $After$ indicates the whole after-display period from 1 to 12 hours, and the hourly after-display period $After$: 12 is omitted due to collinearity. The aggregated ATT estimate was –0.600 (99% CI: [–0.639, –0.557]) when allowing after-display deviation to be twice as large as the maximum before-display deviation ($\overline{M} = 2$, as shown in Figure S16), which is consistent with our main two-period ATT estimate. Furthermore, based on the robust HonestDiD estimate, the predicted reduction in cumulative repost count is, on average, 409, indicating that Community Notes reduced the overall number of reposts on X (formerly Twitter) by 14.4% (median of 9.7%). This is very close to the estimated effect on cumulative repost count in the main analysis (14.9%).

Table S103: Regression results for multi-period ATTs estimation, treating the entire after-display period as a single interval. Post-specific random effects are included. Reported are coefficient estimates with standard errors in parentheses. * $p < 0.01$, ** $p < 0.005$, *** $p < 0.001$. Exact z statistics, p values and 99% CIs are reported in Table S104.

	Multi-period		Continued
Display	1.172***	Display×Before:4	0.043***
	(0.024)		(0.010)
Before:4	0.160***	Display×Before:3	0.044***
	(0.010)		(0.010)
Before:3	0.054***	Display×Before:2	0.027*
	(0.009)		(0.010)
Before:2	0.014	Display×After	–0.927***
	(0.008)		(0.008)
After	–0.100***	PostAge	–0.604***
	(0.025)		(0.015)
After:1	0.367***	Intercept	1.397***
	(0.022)		(0.019)
After:2	0.163***	Post-level RE	✓
	(0.021)		
After:3	0.075***	#Observations	654,400
	(0.019)	#Posts	40,900
After:4	0.044*		
	(0.017)		
After:5	0.016		
	(0.015)		
After:6	0.007		
	(0.013)		
After:7	–0.009		
	(0.011)		
After:8	–0.007		
	(0.010)		
After:9	–0.010		
	(0.008)		
After:10	–0.012		
	(0.007)		
After:11	–0.009		
	(0.006)		
After:12	Omitted		

Table S104: Details of z statistics, p values and 99% CIs (in brackets) for the coefficient estimates reported in Table S103.

	Multi-period
Display	z =48.53, $p < 0.001$ [1.110, 1.234]
Before:4	z =16.55, $p < 0.001$ [0.135, 0.185]
Before:3	z =6.28, $p < 0.001$ [0.032, 0.076]
Before:2	z =1.77, p =0.077 [−0.006, 0.034]
After	z =−3.97, $p < 0.001$ [−0.165, −0.035]
After:1	z =16.32, $p < 0.001$ [0.309, 0.425]
After:2	z =7.94, $p < 0.001$ [0.110, 0.216]
After:3	z =4.03, $p < 0.001$ [0.027, 0.124]
After:4	z =2.60, p =0.009 [0.000, 0.087]
After:5	z =1.04, p =0.301 [−0.023, 0.054]
After:6	z =0.52, p =0.603 [−0.027, 0.041]
After:7	z =−0.81, p =0.420 [−0.039, 0.020]
After:8	z =−0.76, p =0.448 [−0.033, 0.018]
After:9	z =−1.21, p =0.227 [−0.032, 0.011]
After:10	z =−1.74, p =0.081 [−0.031, 0.006]
After:11	z =−1.39, p =0.165 [−0.025, 0.007]
After:12	Omitted

	Continued
Display×Before:4	z =4.21, $p < 0.001$ [0.017, 0.069]
Display×Before:3	z =4.38, $p < 0.001$ [0.018, 0.071]
Display×Before:2	z =2.61, p =0.009 [0.000, 0.053]
Display×After	z =−120.70, $p < 0.001$ [−0.947, −0.907]
PostAge	z =−40.77, $p < 0.001$ [−0.642, −0.566]
Intercept	z =71.91, $p < 0.001$ [1.347, 1.447]
Post-level RE	✓
#Observations	654,400
#Posts	40,900

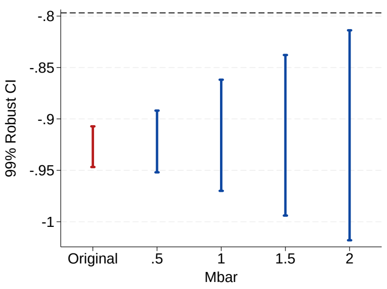

Figure S16: **Robust ATT with HonestDiD.** The original DiD coefficient estimate for the aggregated ATT after the display of community notes and its robust confidence intervals under different Mbars ($\overline{M}$) from 0.5 to 2 with an increment of 0.5.

Supplementary Note 11: Analysis of Deleted and Suspended/Protected Posts

When using X (formerly Twitter) API to retrieve posts fact-checked via community notes, we received return errors for 88,046 posts. Specifically, 64,792 posts were missing due to "not found error", and 23,254 posts were missing due to "authorization error." As detailed in the X (formerly Twitter) API documentation (https://developer.x.com/en/support/x-api/error-troubleshooting), a "not found error" indicates that the corresponding post was deleted by the author of the post. An "authorization error" indicates that the corresponding post was protected by its poster (i.e., private posts) or suspended by the X (formerly Twitter) platform. Since the X (formerly Twitter) API does not distinguish between these two scenarios, we conducted a manual review of 1.500 of these posts. Here, we found that almost all (94.4%) of these inaccessible posts resulted from account suspensions.

11.1 Analysis of Deleted Posts

Regression Discontinuity Design (RDD) is a quasi-experimental research design that aims to estimate the effect of a treatment by exploiting a discontinuity in the relationship between a running variable and an outcome variable at a specific threshold or cutoff point [7, 22]. We used note helpfulness score ($NoteScore$) as a running variable to examine the changes in the probability of post deletion within a narrow window based on a RDD model. The "Community Notes" program adopted several models to determine the helpfulness of community notes, while the Core model was always authoritative [7]. To maintain consistency, we only considered note scores that were decided by the Core Model. The cut-off point of note helpfulness score was 0.40, and community notes that got score of 0.40 or above were displayed on the corresponding misleading posts. We considered the window of note helpfulness scores between 0.35 and 0.45. Additionally, due to the dynamic of note writing and rating, the recalculated note helpfulness scores may be fluctuate

around the cut-off point and not exactly the same with that in the production. Given this, we excluded posts that have community notes with helpfulness score between 0.39 and 0.40 (i.e. [0.39, 0.40)).

Subsequently, we specified our RDD model based on a logistic regression:

$$logit(Deletion_i) = \beta_0 + \beta_1 Display_i + \beta_2 NoteScore_i, \tag{3}$$

where $Display$ indicate whether the note helpfulness score was $\geq$0.40 (=1) or not (=0), and $NoteScore$ represented the running variable of the note helpfulness score. $NoteScore$ was recentered around the cut-off point. The estimation results were reported in Column (1) of Table S105. The coefficient estimate for $Display$ was 0.664 (99% CI: [0.477, 0.851]; z = 9.15, $p < 0.001$). We further examined the treatment effect of community notes display on the post deletion based on the odds ratio estimated for $Display$: $e^{\beta} - 1$, where β denoted the coefficient estimate of $Display$. Specifically, the estimated treatment effect was 0.943 (99% CI: [0.611, 1.342]; z = 9.15, $p < 0.001$). This indicates that posts with displayed community notes were 94.3% more likely to be deleted compared to posts without displayed community notes.

Additionally, we incorporated the interaction between $Display$ and $NoteScore$ in the RDD model to consider the potential different slope over note helpfulness scores after the cut-off point compared to that before the cut-off point. The estimation results are reported in Column (2) of Table S105. The coefficient estimate of $NoteScore$ was 1.164 (99% CI: [−5.080, 7.409]; z = 0.48, $p = 0.631$) and not statistically significant. This indicates that the probability of post deletion remained relatively stable around the cut-off point in the absence of community notes. Moreover, the coefficient estimate for $Display \times NoteScore$ was 4.496 (99% CI: [−2.587, 11.579]; z = 1.64, $p = 0.102$) and not statistically significant. This suggests that community notes had a sharp effect on post deletion once they were displayed on the misleading posts.

Table S105: Estimation results for RDD logistic regression models predicting deletion of posts. Reported are coefficient estimates with standard errors in parentheses. $^{*}\ p < 0.01$, $^{**}\ p < 0.005$, $^{***}\ p < 0.001$. Exact z statistics, p values and 99% CIs are reported in Table S106.

	(1) Main	(2) Interaction
Display	0.664***	0.750***
	(0.073)	(0.090)
NoteScore	4.681***	1.164
	(1.143)	(2.424)
Display×NoteScore		4.496
		(2.750)
Intercept	−2.268***	−2.379***
	(0.045)	(0.082)
#Posts	36,135	36,135

Table S106: Details of z statistics, p values and 99% CIs (in brackets) for the coefficient estimates reported in Table S105.

	(1) Main	(2) Interaction
Display	z =9.15, $p < 0.001$	z =8.31, $p < 0.001$
	[0.477, 0.851]	[0.517, 0.982]
NoteScore	z =4.10, $p < 0.001$	z =0.48, p =0.631
	[1.738, 7.624]	[−5.080, 7.409]
Display×NoteScore		z =1.64, p =0.102
		[−2.587, 11.579]
Intercept	z =−50.29, $p < 0.001$	z =−29.10, $p < 0.001$
	[−2.384, −2.152]	[−2.589, −2.168]
#Posts	36,135	36,135

11.2 Analysis of Protected/Suspended Posts

We applied the same RDD approach used in the analysis of deleted posts to examine the potential effect of community notes on post suspension/protection. The full estimation results are reported in Table S107. The coefficient estimates for $Display$ were not statistically significant in both the Main model ($coef. = 0.072$; 99% CI: [−0.258, 0.401]; $z = 0.56$, $p = 0.575$) and the Interaction model ($coef. = 0.238$; 99% CI: [−0.158, 0.634]; $z = 1.55$, $p = 0.121$). This indicates that the display of community notes had no statistically significant treatment effect on post suspension/protection.

Table S107: Estimation results for RDD logistic regression models predicting suspension/protection of posts. Reported are coefficient estimates with standard errors in parentheses. * $p < 0.01$, ** $p < 0.005$, *** $p < 0.001$. Exact z statistics, p values and 99% CIs are reported in Table S108.

	(1) Main	(2) Interaction
Display	0.072	0.238
	(0.128)	(0.154)
NoteScore	5.646*	−2.130
	(2.064)	(3.976)
Display×NoteScore		10.792
		(4.662)
Intercept	−3.279***	−3.524***
	(0.077)	(0.135)
#Posts	36,135	36,135

Table S108: Details of z statistics, p values and 99% CIs (in brackets) for the coefficient estimates reported in Table S107.

	(1)<br>Main	(2)<br>Interaction
Display	z =0.56, p =0.575<br>[−0.258, 0.401]	z =1.55, p =0.121<br>[−0.158, 0.634]
NoteScore	z =2.74, p =0.006<br>[0.330, 10.963]	z =−0.54, p =0.592<br>[−12.372, 8.112]
Display×NoteScore		z =2.32, p =0.021<br>[−1.216, 22.801]
Intercept	z =−42.80, $p < 0.001$<br>[−3.476, −3.081]	z =−26.09, $p < 0.001$<br>[−3.872, −3.176]
#Posts	36,135	36,135

Supplementary Note 12: Changes in X (formerly Twitter)'s Algorithms and Policies on Misinformation

Our dataset covers all community fact-checked posts written in English between the roll-out of "Community Notes" to the general public on October 6, 2022, and June 11, 2024 (i.e., more than 20 months). During our observation period, X (formerly Twitter) has made several updates to its algorithms and its policies on misinformation. In the following, we provide an overview of the most relevant changes (Supplementary Note 12.1); and detail the steps we took to ensure that our empirical analysis accounts for these changes (Supplementary Note 12.2).

12.1 Timeline of Changes

October 2022: Roll-out of the community notes program to the general public in the U. S. Users who are part of the program can add notes to tweets to add context and users can then vote if they determine the context to be helpful.

December 2022:

- Community notes become visible around the world. Prior to this global expansion, Community Notes were only visible to users in the U. S..
- Twitter disperses the Trust & Safety Council.
- Twitter updated its private information and media policy to prohibit sharing live location information.
- According to posts on Twitter from a former public policy employee, Twitter cut half of its public policy team.

April 2023:

- Twitter label tweets that get downranked for violating its hate speech policy.

- Twitter now shows labels on tweets with reduced visibility.

- Twitter updated it content moderation guidelines regarding hateful content, removing a policy that prohibited the targeted deadnaming or misgendering of transgender people.

- EU warns Twitter over disinformation

May 2023: Twitter launched Community Notes for images in posts.

August 2023: X (formerly Twitter) changes the presentation format of Community Notes. The feature historically provided information to users about why they were seeing a fact check appended to a tweet. After three years of development, the company says this extra context will now be removed for those who are already experienced with Community Notes.

September 2023: Community Notes is now available for videos on X (formerly Twitter). X (formerly Twitter) announced that notes by contributors attached to a video will show up in all posts with that video.

October 2023: A series of updates were made to Community Notes after the fact-checking system took multiple days to correct misinformation related to the Israel-Hamas war. The changes included:

- X (formerly Twitter)'s CEO Linda Yaccarino promised Community Notes would "appear more quickly on X"

- Note previews are now supported on both Android and the web, with support "coming soon" to iOS

- Improved media matching

- Notifications will send to users who Liked, Reposted or Replied to a post that later received a note

January 2024: X (formerly Twitter) announces plans to hire 100 moderators in Austin.

June 2024: Adult content and violent content policies replace the former sensitive media and violent speech policies.

12.2 Accounting for Algorithmic Effects in Our Study

We carefully accounted for potential algorithmic effects across all aspects of our study. This included (i) reviewing X (formerly Twitter)'s recommendation algorithm, (ii) employing robust model specifications, (iii) conducting comprehensive sensitivity analyses, and (iv) differentiating between user-initiated and platform-enforced actions. Together, these measures ensure that the observed changes in resharing behavior after a Community Note becomes visible are driven by shifts in user behavior rather than platform interventions.

- **Review of X (formerly Twitter)'s recommendation algorithm**: X (formerly Twitter)'s FAQs state that "*Notes that have been rated helpful by enough contributors from different points of view will appear directly on posts. Beyond that, notes do not affect display of posts or enforcement of X's rules*" (https://communitynotes.x.com/guide/en/about/faq). Furthermore, X (formerly Twitter)'s recommendation algorithm is available as open source (https://github.com/twitter/the-algorithm). Consistent with X (formerly Twitter)'s FAQs, the algorithm does not impose any penalties (e.g., visibility reduction) or trigger enforcement of X (formerly Twitter)'s rules (e.g., account suspensions) on posts flagged with community notes. Consequently, any observed changes in resharing behavior after a community note becomes visible must be attributed to shifts in user behavior, rather than algorithmic intervention.

- **Model specification:** Our model explicitly accounts for algorithmic changes by allowing the treatment effect to vary over time. To achieve this, all our models incorporate a comprehensive set of control variables as well as post-specific random effects (see Methods). Ad-

ditionally, our propensity score matching method ensures that we compare only posts with and without displayed notes that were published within a similar timeframe (i.e., during the same month).

- **Analysis of treatment effect over time:** As part of our sensitivity analyses, we specifically examined how the efficacy of Community Notes has evolved since the feature's rollout in October 2022 (see Results). Our analysis revealed a trend of increasing efficacy over time, which may be partly attributed to improvements within the Community Notes program, such as faster note generation.

- **Analysis of suspensions:** We investigated the potential impact of Community Notes on account suspensions (see Results). Unlike post deletions (i.e., user-initiated actions), we observed no significant change in the ratio of suspended posts (i.e., platform-enforced actions).

References

[1] Wojcik, S. *et al.* Birdwatch: Crowd wisdom and bridging algorithms can inform understanding and reduce the spread of misinformation. *arXiv* (2022).

[2] X. Note ranking algorithm. https://communitynotes.twitter.com/guide/en/under-the-hood/ranking-notes (2024).

[3] X. Twitter's recommendation algorithm. https://blog.x.com/engineering/en_us/topics/open-source/2023/twitter-recommendation-algorithm (2023).

[4] X. Faqs for Community Notes program. https://communitynotes.x.com/guide/en/about/faq (2024).

[5] Chuai, Y. & Zhao, J. Anger can make fake news viral online. *Frontiers in Physics* **10**, 970174 (2022).

[6] Pfeffer, J., Matter, D. & Sargsyan, A. The half-life of a tweet. In *ICWSM* (2023).

[7] Chuai, Y., Tian, H., Pröllochs, N. & Lenzini, G. Did the roll-out of Community Notes reduce engagement with misinformation on X/Twitter? *Proceedings of the ACM on Human-Computer Interaction* **CSCW2** (2024).

[8] International Components for Unicode. *ICU Documentation*. Unicode, Inc. (2024). https://unicode-org.github.io/icu/.

[9] Loureiro, D., Barbieri, F., Neves, L., Anke, L. E. & Camacho-Collados, J. TimeLMs: Diachronic language models from Twitter. *arXiv* (2022).

[10] X. Downloading data. https://communitynotes.twitter.com/guide/en/under-the-hood/download-data (2024).

[11] Vosoughi, S., Roy, D. & Aral, S. The spread of true and false news online. *Science* **359**, 1146–1151 (2018).

[12] Zhang, X. *et al.* TwHIN-BERT: A socially-enriched pre-trained language model for multilingual tweet representations at Twitter. *arXiv* (2022).

[13] Wolf, T. *et al.* Transformers: State-of-the-art natural language processing. *ACL Anthology* 38–45 (2020).

[14] Guo, S. & Fraser, M. W. *Propensity score analysis: Statistical methods and applications*, 11 (SAGE Publications, 2014).

[15] Bilinski, A. & Hatfield, L. A. Nothing to see here? non-inferiority approaches to parallel trends and other model assumptions. *arXiv* (2018).

[16] Roth, J., Sant'Anna, P. H. C., Bilinski, A. & Poe, J. What's trending in difference-in-differences? A synthesis of the recent econometrics literature. *Journal of Econometrics* **235**, 2218–2244 (2023).

[17] Lakens, D., Scheel, A. M. & Isager, P. M. Equivalence testing for psychological research: A tutorial. *Advances in Methods and Practices in Psychological Science* **1**, 259–269 (2018).

[18] Dette, H. & Schumann, M. Testing for equivalence of pre-trends in difference-in-differences estimation. *Journal of Business & Economic Statistics* **42**, 1289–1301 (2024).

[19] Lakens, D. Equivalence tests: A practical primer for t tests, correlations, and meta-analyses. *Social Psychological and Personality Science* **8**, 355–362 (2017).

[20] Rambachan, A. & Roth, J. A more credible approach to parallel trends. *Review of Economic Studies* **90**, 2555–2591 (2023).

[21] Mosleh, M. & Rand, D. G. Measuring exposure to misinformation from political elites on Twitter. *Nature Communications* **13**, 7144 (2022).

[22] Angrist, J. D. & Pischke, J.-S. *Mostly harmless econometrics: An empiricist's companion* (Princeton University Press, 2009).
