# Supplementary material for: Community-based fact-checking reduces the spread of misleading posts on X (formerly Twitter)
Source: Nat Commun. 2026 May 5;17:4070. doi: 10.1038/s41467-026-72597-0 (PMC13144318; doi:10.1038/s41467-026-72597-0)
Supplement: Supplementary file 2 — Reporting Summary [file 41467_2026_72597_MOESM2_ESM.pdf]

nature portfolio

Corresponding author(s): Nicolas Pröllochs

Last updated by author(s): Feb 6, 2026

Reporting Summary

Nature Portfolio wishes to improve the reproducibility of the work that we publish. This form provides structure for consistency and transparency in reporting. For further information on Nature Portfolio policies, see our Editorial Policies and the Editorial Policy Checklist.

Statistics

For all statistical analyses, confirm that the following items are present in the figure legend, table legend, main text, or Methods section.

n/a	Confirmed	
☐	☒	The exact sample size (*n*) for each experimental group/condition, given as a discrete number and unit of measurement
☐	☒	A statement on whether measurements were taken from distinct samples or whether the same sample was measured repeatedly
☐	☒	The statistical test(s) used AND whether they are one- or two-sided<br>*Only common tests should be described solely by name; describe more complex techniques in the Methods section.*
☐	☒	A description of all covariates tested
☐	☒	A description of any assumptions or corrections, such as tests of normality and adjustment for multiple comparisons
☐	☒	A full description of the statistical parameters including central tendency (e.g. means) or other basic estimates (e.g. regression coefficient) AND variation (e.g. standard deviation) or associated estimates of uncertainty (e.g. confidence intervals)
☐	☒	For null hypothesis testing, the test statistic (e.g. *F*, *t*, *r*) with confidence intervals, effect sizes, degrees of freedom and *P* value noted<br>*Give P values as exact values whenever suitable.*
☒	☐	For Bayesian analysis, information on the choice of priors and Markov chain Monte Carlo settings
☒	☐	For hierarchical and complex designs, identification of the appropriate level for tests and full reporting of outcomes
☐	☒	Estimates of effect sizes (e.g. Cohen's *d*, Pearson's *r*), indicating how they were calculated

Our web collection on statistics for biologists contains articles on many of the points above.

Software and code

Policy information about availability of computer code

Data collection	The data was collected via the X/Twitter Pro API.
Data analysis	We used Python 3.11.3 to conduct our empirical analyses. Our regression models were implemented using the pystata Python package with Stata 19.5 MP-parallel edition (2-core network). Code to replicate the findings of our study is available at https://doi.org/10.17605/OSF.IO/M642D.

For manuscripts utilizing custom algorithms or software that are central to the research but not yet described in published literature, software must be made available to editors and reviewers. We strongly encourage code deposition in a community repository (e.g. GitHub). See the Nature Portfolio guidelines for submitting code & software for further information.

Data

Policy information about availability of data

All manuscripts must include a data availability statement. This statement should provide the following information, where applicable:

- Accession codes, unique identifiers, or web links for publicly available datasets
- A description of any restrictions on data availability
- For clinical datasets or third party data, please ensure that the statement adheres to our policy

Upon publication of this work, all data and materials needed to recreate the analysis will be made available via OSF.

Research involving human participants, their data, or biological material

Policy information about studies with human participants or human data. See also policy information about sex, gender (identity/presentation), and sexual orientation and race, ethnicity and racism.

Reporting on sex and gender	We did not collect sex/gender of the participants/users in this study.
Reporting on race, ethnicity, or other socially relevant groupings	We did not collect race, ethnicity or other socially relevant information of the participants/users in this study.
Population characteristics	We controlled for users' public account characteristics on X/Twitter. We did not estimate or collect biographical information of users.
Recruitment	We collected the account information of users whose posts were fact-checked by community notes on X/Twitter between October 6, 2022 and June 11, 2024. The number of user accounts in our study is 60,815.
Ethics oversight	This research has received ethical approval from the Ethics Review Panel of the University of Luxembourg (ref. ERP 23-053 REMEDIS). All analyses are based on publicly available data.

Note that full information on the approval of the study protocol must also be provided in the manuscript.

Field-specific reporting

Please select the one below that is the best fit for your research. If you are not sure, read the appropriate sections before making your selection.

☐ Life sciences ☒ Behavioural & social sciences ☐ Ecological, evolutionary & environmental sciences

For a reference copy of the document with all sections, see nature.com/documents/nr-reporting-summary-flat.pdf

Behavioural & social sciences study design

All studies must disclose on these points even when the disclosure is negative.

Study description	We performed a large-scale quasi-experimental study to assess the real-world efficacy of community-based fact-checking on the social media platform X/Twitter.
Research sample	Our analysis is based on repost time series data for N=237,180 community fact-checked cascades (posts) from 60,815 users that have been reposted more than 431 million times.
Sampling strategy	We collected *all* community fact-checked posts and their repost time series within a period of over 20 months from the roll-out of "Community Notes" on October 6, 2022 to June 11, 2024.
Data collection	We used the X/Twitter Pro API to collect community fact-checked posts and their associated user information.
Timing	We collected the data in July 2024.
Data exclusions	We did not exclude any publicly available posts. Posts that were not publicly available at the time of data collection (e.g., deleted by the authors, suspensions) were analyzed separately. For our Difference-in-Differences (DiD) analysis, we performed one-to-one propensity score matching to construct a control group that was balanced with the treatment group across all poster and post characteristics. This step was necessary to reduce confounding and ensure parallel trends between treatment and control groups before the intervention.
Non-participation	Our study is quasi-experimental and based on social media data. We included all publicly available posts that were community fact-checked between October 6, 2022 and June 11, 2024.
Randomization	We conducted propensity score matching and controlled for a series of post and poster characteristics to reduce confounding and ensure parallel trends in the treatment and control groups before the display of community notes on X/Twitter.

Reporting for specific materials, systems and methods

We require information from authors about some types of materials, experimental systems and methods used in many studies. Here, indicate whether each material, system or method listed is relevant to your study. If you are not sure if a list item applies to your research, read the appropriate section before selecting a response.

Materials & experimental systems

n/a	Involved in the study
☒	☐ Antibodies
☒	☐ Eukaryotic cell lines
☒	☐ Palaeontology and archaeology
☒	☐ Animals and other organisms
☒	☐ Clinical data
☒	☐ Dual use research of concern
☒	☐ Plants

Methods

n/a	Involved in the study
☒	☐ ChIP-seq
☒	☐ Flow cytometry
☒	☐ MRI-based neuroimaging

Plants

Seed stocks	N/A
Novel plant genotypes	N/A
Authentication	N/A
